# Looking for future biological control agents: the comparative function of the deutosternal groove in mesostigmatid mites

**DOI:** 10.1007/s10493-023-00832-0

**Published:** 2023-09-07

**Authors:** Clive E. Bowman

**Affiliations:** Mathematical Institute, University of Oxford, Oxford, OX2 6GG UK

**Keywords:** Acari, Airorhynchid, BCA, Biological control agent, Capillarity, Feasibility, Feeding design, Fluid dynamics, Goldilocks zone, Hypognathal denticles, Microchannel, Pressure-driven flow, Vortex generators, Wavelength

## Abstract

The physics of fluid laminar flow through an idealised deutosternum assembly is used for the first time to review predatory feeding designs over 72 different-sized example species from 16 mesostigmatid families in order to inform the finding of new biological control agents. Gnathosomal data are digitised from published sources. Relevant gnathosomal macro- and micro-features are compared and contrasted in detail which may subtly impact the control of channel- or ‘pipe’-based transport of prey liquids around various gnathosomal locations. Relative deutosternal groove width on the mesostigmatid subcapitulum is important but appears unrelated to the closing velocity ratio of the moveable digit. Big mites are adapted for handling large and watery prey. The repeated regular distance between deutosternal transverse ridges (‘Querleisten’) supports the idea of them enabling a regular fluctuating bulging or pulsing droplet-based fluid wave ‘sticking’ and ‘slipping’ along the groove. Phytoseiids are an outlier functional group with a low deutosternal pipe flow per body size designed for slot-like microchannel transport in low volume fluid threads arising from daintily nibbling nearby prey klinorhynchidly. Deutosternal groove denticles are orientated topographically in order to synergise flow and possible mixing of coxal gland-derived droplets and circumcapitular reservoir fluids across the venter of the gnathosomal base back via the hypostome to the prey being masticated by the chelicerae. As well as working with the tritosternum to mechanically clean the deutosternum, denticles may suppress fluid drag. Shallow grooves may support edge-crawling viscous flow. Lateral features may facilitate handling unusual amounts of fluid arising from opportunistic feeding on atypical prey. Various conjectures for confirmatory follow-up are highlighted. Suggestions as to how to triage non-uropodoid species as candidate plant pest control agents are included.

## Introduction

The search for new biological control agents (BCAs) against world-wide plant pests (e.g., tetranychids, eriophyids, tenuipalpids, scale insects, thrips, etc.) is a constant challenge. Often soil predatory mites are examined for their potential as candidates (Beretta et al. 2022) following the Goldilocks principle in being neither too big, nor too small, but just right. Life style is an important feature in evaluating their potential (Liu et al. 2017). As a general rule, macro-predators consume microbiovores whilst micro-predators eat detritivores and herbivores (Crotty et al. 2014). So, as well as having the appropriate reproductive time-course response and being able to access and pursue prey on a webbed plant, one is seeking a modestly sized suitably trophically designed mesofaunal agent for the task. A mite with long legs would enable it to move long distances between plants with relatively little effort seeking out pests. A mite which does not itself attack the plant like some phytoseiids have been reported to (Adar et al. 2012) would be desirable. A mesostigmatid morphology that encourages voracity (i.e., potential rapid sequential feeding on prey to decimate pest infestations) would be helpful. As Crotty et al. (2014) points out that grasslands are typified by herbivores, microbiovores and small predators whilst forests are typified by omnivores and large predators, biomes like the former should be targeted in any search for new agents.

There are limited practical methods to evaluate candidate mites without detailed biological experiments (Liu et al. 2017). Based upon the morphological design of successful phytoseiid BCAs, using characters from Bowman (2020), a crushing feeding style chela (i.e., with the length of its moveable digit closing adductive input moment arm divided by the length of its output moment arm or velocity ratio $VR\ge 0.4$) and a reasonable ‘gape’ between the fixed and moveable digit surfaces is expected (i.e., $MDL>24\ \upmu$m) especially for specialist oophages (which may grasp eggs whole). A cheliceral *F*2 crushing force not less than that of the weaker predatory phytoseiids ($407.7\ \upmu$m$^{2}$) would also be appropriate for a new BCA.

However, using these key trophic design summary parameters in Bowman (2020) of *VR* and *F*2 reduces the 60 free-living species therein to just 7 potential BCA candidates, some of which are almost certainly fungivores. Having a requirement of slavishly matching the phytoseiid cheliceral design as an effective way of generating new candidate BCAs may not be an efficient way forward. For sure, a reasonable compensatory cheliceral reach (*CLI*; Bowman 2020), in particular relative to body size, could facilitate capturing prey unawares at a distance before they flee, even if the chelal mastication process once prey is captured has a different efficiency (*VR*) than phytoseiids. This is informally validated by the success of using elongate rhodacarids in tetranychid control trials. Looking at the same 60 free-living species in Bowman (2020) with a reach bigger than $108\ \upmu$m ($\equiv$*Typhlodromus setubali*) and a powerful enough crunch force now generates 55 candidates. What other gnathosomal features might impact future potential BCA choice?

**Fig. 1:**
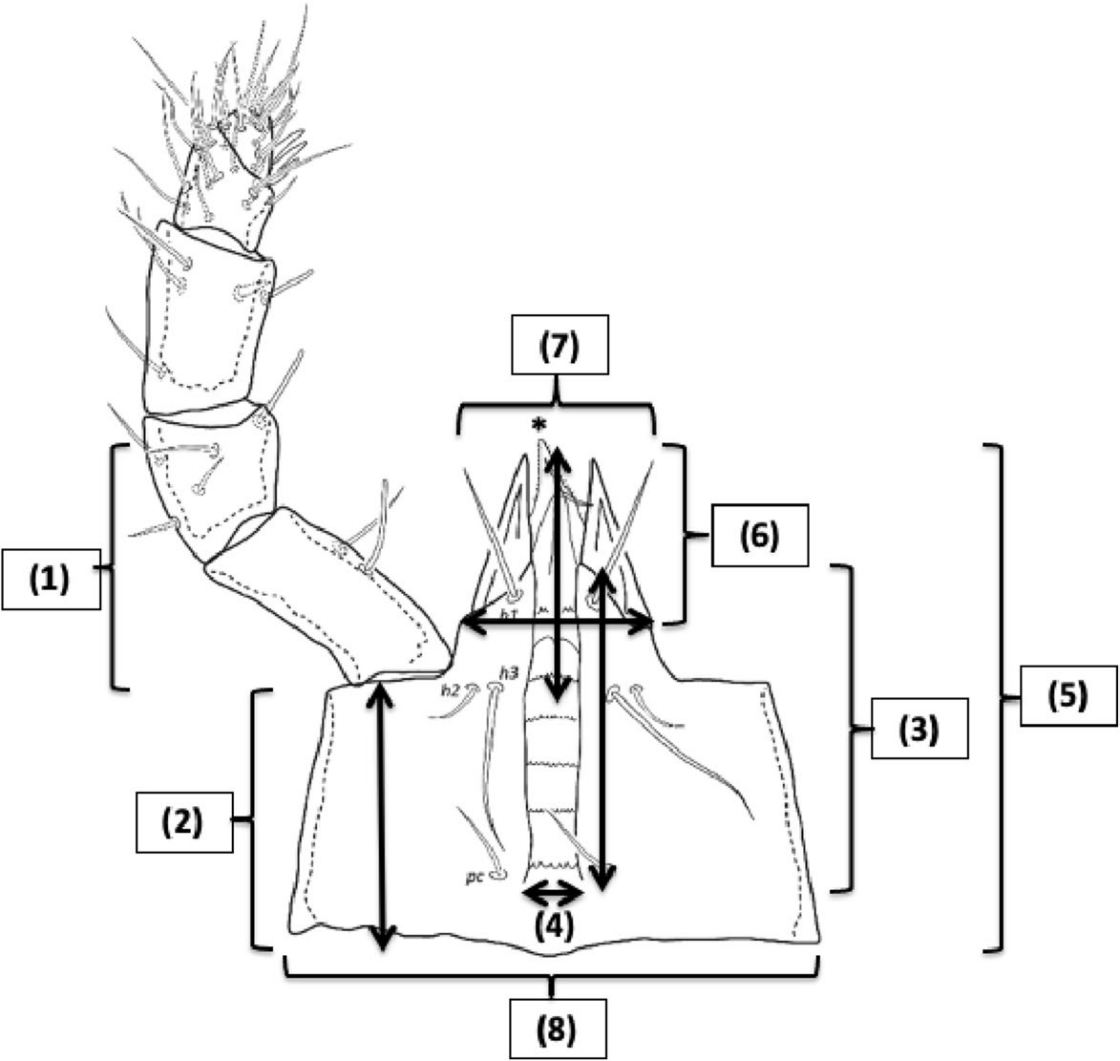
Venter of mesostigmatid gnathosoma sensu Krantz and Walter (2009) ignoring the generally soft, pliable or membraneous internal malae. Measurements made numerically coded in boxes. Scheme of subcapitular module based upon amended drawing of female *Macrocheles willowae* subcapitulum and palp by Knee (2017) https://zookeys.pensoft.net/article/21747/list/2/ under Creative Commons Attribution (CC-BY) licence. *Tip of the labrum. (1) = Length of the hypostome (Evans and Till 1979; Evans 1992) reaching to the end of the corniculi (external malae). The posterior point may be behind the palp trochanter if there are deep folds on either side. Note, herein it always includes the insertions of setae $h_{1}-h_{3}$. (2) = Length of the *basis gnathosomatica* (Evans and Till 1979; Evans 1992) from extreme posterior point reaching forward to pedipalpal junction. It can be referred to as *basis capitulum* or subcapitulum base. (3) = Length of the deutosternal groove herein determined by its longitudinal edges or envelope of central ridges and denticles. Denoted as *GL*. Anterior edge can be at bases of internal malae or even posterior of cornicular bases. Posterior limit can be short of the circumcapitular groove. (4) = Deutosternal (average) width denoted *AGW*, determined herein by its longitudinal edges or if absent by position of the most extreme central denticles. Ancillary lateral grooves, folds and other ridged/denticulated features not included. (5) = Length of the subcapitulum or hypognathum (i.e., herein to tips of corniculi). (6) = Length of corniculi (note these may be highly reduced or internalised). (7) = Maximum width of hypostome at bases of corniculi. (8) = Maximum width of *basis gnathosomatica* or hypognathum, denoted here as *BGW*. Note (1) + (2) $\approx$ length of subcapitulum (5). Salivary styli are omitted

Identification of new BCA species requires expertise that is becoming more and more rare (Diana Rueda-Ramírez and Eric Palevsky *pers. comm.*). There have been attempts before to relate phytoseiid predatory success with adaptations in their mouthparts (Ragusa and Tsolakis 2000). Could the structure of the mite’s subcapitulum as a part of the gnathosoma be important for successful feeding upon plant pests? The maximum extent [front–back, left–right] of the mesostigmatid subcapitulum is broadly square. Embedded within it anteriorly is the almost equilateral triangular hypostome (Fig. 1). Traditionally the projecting internal malae, the corniculi and various setae are all included as a part of the hypostome (Krantz and Walter 2009). Amongst the acarines, Mesostigmata are typified by having a sub-capitular tritosternum socketed close to the circum-gnathosomal groove at the junction of the gnathosoma with the idiosoma. The tritosternum is part of the 3rd segment of the mite, and the subcapitulum is the 2nd segment (comprising of fused palp coxae etc.; Krantz and Walter 2009). So the tritosternum as part of the idiosoma works with the subcapitulum but is not a subsidiary part of it. The venter of the subcapitulum (infracapitulum; van der Hammen 1989) nearly always (Krantz and Walter 2009) has a clearly visible medial longitudinal deutosternal groove (or ‘gutter’) lying parallel but opposed to the tritosternum with its usually pilose lacinae (Wernz 1972) extending right up to the cornicular bases within the ‘beak-like’ hypostome (e.g., in *Pergamasus longicornis*; Bowman 1984). Note that in this review, the morphological terms: mentum, and genae, are not used.

The deutosternum is sometimes denoted as deuterosternum by some acarologists (this study, however, follows Krantz and Walter 2009). It can have chitinous longitudinal edges that permit fluid flow between their margins (Wernz and Krantz 1976) and denticulated transverse ridges or ‘cross-bars’ (Querleisten; Hirschmann 1962) standing proud of its surface. Wernz (1972) found the longitudinal groove itself to have a convex surface with lateral edges in-folded making the edges like tiny (nano?) channels in their own right with which the tritosternal lacinae interact. He also claimed that the position of the anterior-most deutosternal teeth was fairly constant over species being near the level of hypostomal seta $h_{3}$. In phytoseiids, over the three accepted life-style groupings, the tritosternum is on average marginally longer than the deutosternal groove ($\approx 59\ \upmu$m versus $\approx 49\ \upmu$m respectively; Liu et al. 2017) unlike in other mesostigmatids where it is equal or two thirds of the length of the deutosternal groove (Wernz 1972). The phytoseiid deutosternal groove, or infracapitular gutter (Alberti and Coons 1999), is usually relatively narrow (Flechtmann and McMurtry 1992a). Flechtmann and McMurtry (1992b) note that the deutosternal grooves in the generalist predators *Iphiseius degenerans* and *Euseius* spp. are $\approx 7{-}9\ \upmu$m wide, whereas in other specialist species they are usually $4{-}6\ \upmu$m. Is this relative narrowness a key to phytoseiid predatory success? The tritosternum is considerably wider seemingly resting in a concavity in the adjacent area of the gnathosomal base in some species (e.g., *Phytoseiulus longipes*; Flechtmann and McMurtry 1992b). The question arises what is the function of these two structures and might they be of relevance to possible BCA competency?

How things work matters. Outside of taxonomic uses, the deutosternal groove has been a neglected area when comparing mites. Like other arachnids, mesostigmatids employ, in part, pre-oral digestion (Dunlop 1994; Bowman 2019). Plant pest prey like tetranychids and nematodes may be watery when ruptured by the predator’s chelicerae during feeding. The ability to handle this moist tissue well, again and again, may be key in determining the effectivity of repeated attacks by a predator. Are phytoseiids designed just right to handle tetranychid prey fluids? Wernz and Krantz (1976) notes that the volume of liquid ordinarily released at the moment of prey puncturing is far more than the amount that can immediately be taken up by the predatory mites *Parasitus coleoptratorum* and *Glyphtholaspis americanum* mouthparts. They also showed that tritosternumectomy caused a change of predatory feeding efficiency in *P. coleoptratorum*. Acarologists popularly interpret this as the tritosternum being involved with the deutosternum as a longitudinally flowing tube or ‘pipe’ for the control of the circumcapitular prey fluid pool that is believed to overspill from the hypostomal area during feeding (Walter and Proctor 2013). Care with prey-derived fluids is important as Wernz (1972) points out that neither *Parasitus* sp. nor *Glyphtholaspis* sp. drank from water drops. Now Tian et al. (2022) says: “After a long period of biological evolution, natural creatures will inevitably evolve body surfaces suitable for the living environment”. What might this all mean for gnathosomal surfaces in mesostigmatids?

Flechtmann and McMurtry (1992a) describe unidirectional liquid flow from prey to predator. Wernz (1972) confirmed with dyeing experiments that orally connected fluids do accumulate in the circumcapitular groove and observed larger amounts if mites fed successfully on multiple prey. For water-repellent surfaces like many arthropod cuticles (Hensel et al. 2016), when a liquid droplet is placed on the solid surface, it could retain the form of a droplet or alternatively spread out on the surface to form a thin liquid film. Now what might happen to fluids on the subcapitulum surface? The channel form of the sub-capitular deutosternum and its location within the subcapitulum varies in mesostigmatids. Is this of relevance to the handling of fluids by BCAs? The advantage of a water-repellent surface in any such cuticular groove would be the ability in minimising the flow resistance in order to gain higher mass flow rates (in a narrow medium such as a micro-tube) when an external power source is restricted. Pharyngeal pumping is the source of ingestion in mesostigmatids who can have extended feeding times. Could this all be relevant to subcapitular fluid handling? Recently, such nano- and micro-structured water-repellent surfaces have been studied (Hensel et al. 2016) and employed in various practical applications such as ‘lab on a chip’ technology (Tan et al. 2020).

Walter and Proctor (1998) point out that well developed tritosterna are a feature of predatory fluid feeders. Wernz (1972) highlights correlations of tritosternal form with feeding behaviour (his Fig. 16) and considers the volume between the tritosternum and deutosternum as a prey fluid overflow store (or ‘buffer’). Bowman (2014) posed the role of the deutosternum/tritosternum assembly in recycling coxal fluid back into gnathosomal area where the prey is being masticated, illustrating various examples of *P. longicornis* in the field.

So a question arises: what relative deutosternal flow rates (i.e., fluid moving under the tritosternum) might there be between mite species?

Indeed, can a modest feasibility study provide initial insight as to whether a mesostigmatid’s predatory adaptation can be inferred from the size and shape of its deutosternal groove? If so then, how does such integrate: hypostomal-dependent mechanisms, *basis gnathosomatica* design and the form of the chelicerae and so impact feeding efficiency? Could this all inform selection of putative new BCAs from the pool of possible soil mesostigmatids?

Water-active properties of materials are common in the morphologically variable arachnid sister-group Insecta (Schroeder et al. 2018). Whilst diverse gnathobasal/hypostomal forms with various hierarchical (Hensel et al. 2016) macro- and micro-structures (e.g., setae, ridges, grooves, scales, denticles, etc.) have been described by various acarologists, this requires a quantitative numerical framework to investigate such small-scale fluid handling abilities objectively and how they might relate to previously reported cheliceral adaptations (Bowman 2020).

### Quantitative model

**Fig. 2:**
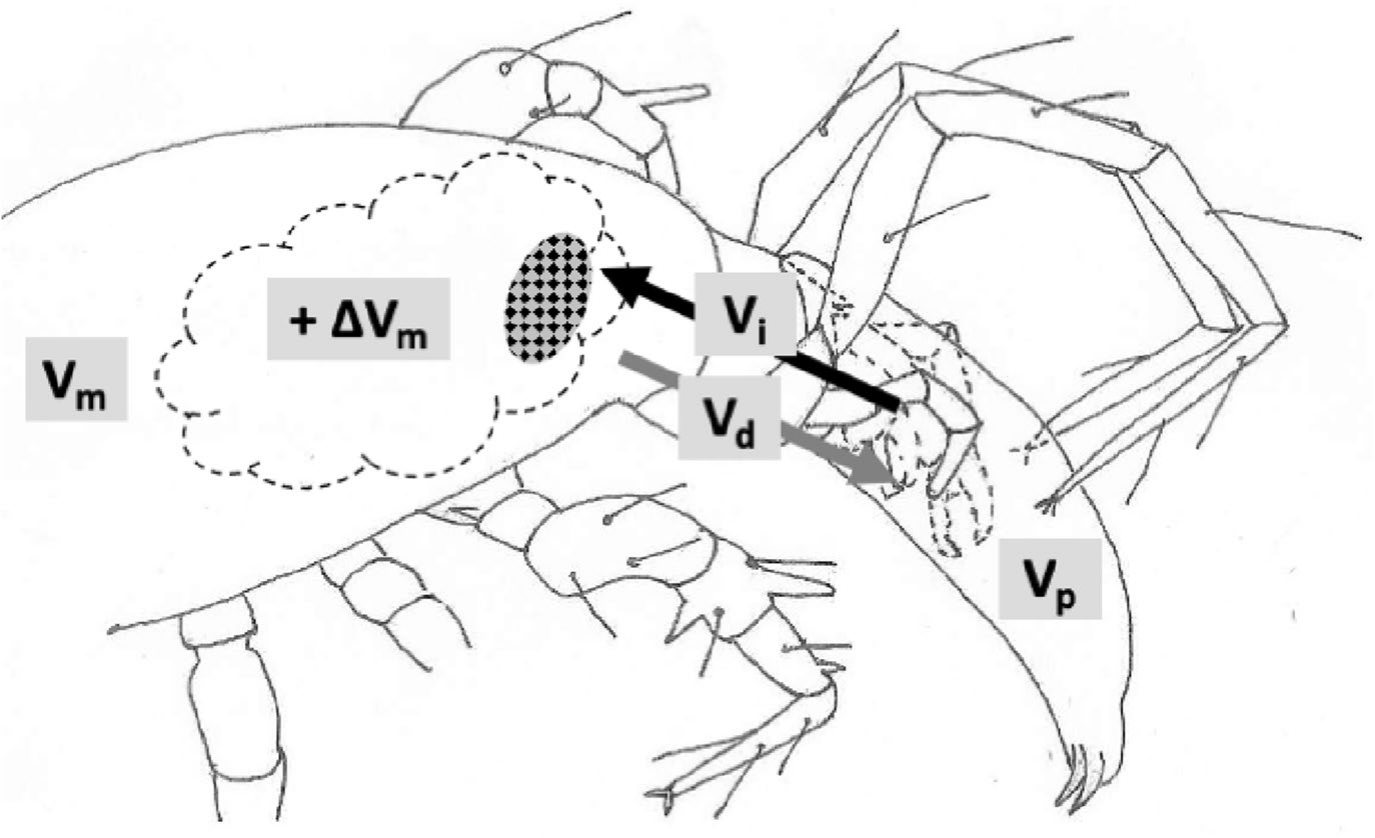
Volumetric model of pergamasid of initial body volume $V_{m}$ feeding on larval dipteran prey of initial body volume $V_{p}$ (adapted from Fig. 8 Bowman 2014$\copyright$ Springer Nature with permission). Hashed area: ingested food and prey fluids in multilobate gut. $V_{p}$ prey volume. $V_{m}$ fasted mite body volume, and change due to feeding ($\varDelta V_{m}$). Black arrow: $V_{i}$ volume of ingestion (prey liquids and fluidised tissue). Grey arrow: $V_{d}$ volume of recycled coxal fluids from haemocoel and circumcapitular overspill of prey liquids back through the deutosternum/tritosternum assembly. Each feeding volume can be considered at a time *t* from the start of feeding e.g., $V_{d(t)}$, and the corresponding rate $\frac{\delta V_{d(t)}}{\delta t}$ at that time t. In this figure the micro-details of structures and their regionalisation are ignored

As a first step of abstraction, Fig. 2 illustrates a simple fluid balance schema for a predatory mesostigmatid where the prey is volumetrically smaller than the predator (it also ignores any salivary processes or transpirational losses). Prey tissues (bar cuticular exoskeleton) are assumed to be enzymically liquidised and to be effectively dissolved into the imbibed prey fluids with no major increase in the effective prey volume ($V_{p}$) during predation. Defining $\varDelta V_{m}$ as the change in predator volume, if no rectal excretion of fluid occurs (and no coxal droplets are discarded by leg adjustments; Bowman 2014) then $\varDelta V_{m} \le V_{p}$ must be true. The instantaneous net imbibition rate (at time *t*) $NIR_{t}$ (over time $\delta t$) equals $\frac{\delta V_{i(t)}}{\delta t}-\frac{\delta V_{d(t)}}{\delta t}$ where the two rates may be linear or exponential functions of time and $\varDelta V_{m}=\int _{t=0}^{end_{feed}} NIR_{t}\ \textrm{d}t$.

Unlike for a slowly feeding parasitic mesostigmatid with its abundant continuous supply of food (like *Varroa destructor*), a rapacious predator episodically attacking prey like plant pest tetranychids and nematodes needs to have a high ingestion rate $\frac{\delta V_{i(t)}}{\delta t}$ at the beginning of feeding in order to rapidly subdue the prey and get the potential nutrients (which flood over and between the chelicerae, e.g., in *Stratiolaelaps scimitus*; Eric Palevsky *pers. comm.*) rapidly into the gut to begin speedy digestive processing (Bowman 2017). However, living within an arthropod’s chitinous ‘suit-of-armour’ has challenges for a mesostigmatid. Predator body volume increase may be difficult, if not severely restricted to just slow idiosomal expansion as the folded cuticle surface of a fasted mite uncrinkles. So $\frac{\delta V_{m(t)}}{\delta t}$ must be modest over the period to that time *t*, even if the final $\varDelta V_{m}$ may be a reasonably large proportion of $V_{m}$ (for instance in a mite with just one long duration single feeding bout). This can only be achieved if $\frac{\delta V_{d(t)}}{\delta t}$ is also substantial. In that way peak deutosternal/tritosternal fluid flow effectively controls maximum imbibition at the start of feeding.

Overflow of prey fluids into the deutosternum (Wernz 1972) or into the circumcapitular groove (postcapitular channel; Wernz and Krantz 1976) does not in itself facilitate rapid imbibition, rather it simply acts as a temporary depot or ‘buffer store’. $\frac{\delta V_{d(t)}}{\delta t}$ needs only be slightly smaller than $\frac{\delta V_{i(t)}}{\delta t}$ or there be a slight delay in phase between its time course and that of ingestion for the coxal mechanism proposed by Bowman (2014) to be effective. Such could be furnished by the distributional delay of liquid moving out of the gut and into the coxal gland tubules before intercoxal filtration or secretion. Later of course when the mite is taking in more structural tissue-derived material from a quiescent moribund carcass (Bowman 2017), then the ingestion rate can be slower. Despite phytoseiids feeding on fluid droplets in vitro (Ghazy and Suzuki 2019), it is worth pointing out that no excess external flow of body fluids from spider mite prey (encompassing the gnathosoma) during feeding episodes in phytoseiids themselves have yet been observed (Flechtmann and McMurtry 1992a). This may mark their feeding out as being different to soil-inhabiting pergamasids.

For the purposes of this review paper, let us define the primary structure of the deutosternum/tritosternum assembly to be the stream-wise longitudinal lateral edges of the deutosternal groove (on which the tritosternal lacinae lie and run along; Wernz 1972). Then the secondary structure to be the grooves’ denticulated transverse interconnecting ridges (span-wise to the postulated flow) to yield a ladder-like form to it, and the tertiary structure to be the (often) plumose or fimbriate tritosternum. An abstraction to a non-hierarchical simplified structure, as a preliminary, helps disentangle and dissect out the various possible functions.

Let us assume that the end of mite feeding ($end_{feed}$) scales with the size of prey, i.e., with their total volume $V_{p}$ and therefore also approximately with $\varDelta V_{m}$ (from the inequality above). This is a reasonable constraint for a poikilotherm and is supported in Wernz (1972) where tritosternectomised mites have the same feeding bout times as normal mites but just feed more often. Further that idiosomal expandability $\left( \frac{V_{m}+\varDelta V_{m}}{V_{m}}\right)$ is a constant proportion over mite original pre-feeding sizes [$V_{m,(t=0)}$], i.e., bigger mites can expand more than smaller predators but only in relation to their original volume, again a reasonable simplification. Then define the total deutosternal volume over feeding scaled by body size for any mite as a key comparative trophic efficiency parameter (denoted here as *tep*), i.e.,

$$\frac{V_{d}}{V_{m}}=tep,$$

where $$V_{d}=\int _{t=0}^{end_{feed}} V_{d(t)}\ \textrm{d}t$$.

The denominator $V_{m}$ equals $\frac{\pi }{2}IL^{3}$ assuming an idiosomal volume of approximately a cylinder where *IL* is the idiosomal index length (Bowman 2023).

For the numerator, imagine that the tritosternum is raised dorsally towards the subcapitular surface and pose the primary-tertiary structure of the assembly acts as a capillary tube, i.e., a long cylindrical ‘pipe’ for the uniaxial transport of fluid. Wernz (1972) describes such capillarity occurring in his feeding observations of macrochelids and parasitids. The fluid in this pipe is prevented from spreading laterally over the subcapitulum by ‘pinning’ (Theodrakis et al. 2021) on the longitudinal ridges, and it is held together along its open liquid–air sides by surface tension to make an effective wall. Its roof is the long tritosternum just as Wernz (1972) posed. This ‘conduit’ then continuously extends from the ventral ridged inter-coxal leg 1 area through to the hypostomal tip (as confirmed with dyeing experiments by Wernz 1972) and onto the labrum (on gnathosomal retraction or ‘telescoping’). It is also assumed that only a low pumping power is available. Denote this as the ‘deutosternal pipe’, then the Hagen–Poiseuille Law for an incompressible Newtonian uniform non-volatile fluid in steady low Reynolds number laminar flow through a long cylindrical pipe of constant cross section states, that

$$\varDelta p = \frac{8 \upmu L Q}{\pi R^{4}},$$

where $\varDelta p$ is pressure difference between the two ends, $\mu$ is dynamic viscosity, *L* is length of pipe, *Q* is volumetric flow rate, and *R* is the pipe radius. So the flow rate

$$Q =\frac{\varDelta p. \pi R^{4}}{8 \upmu L}.$$

Note the inverse relationship of groove lengthening (*L*) and pressure drop ($\varDelta p$). Then, for constant dynamic viscosity ($\mu$) and pressure difference across mite species this can be simplified (by dropping all of the constants without loss of generality and swopping notation) to the ‘deutosternum pipe flow’ (over its whole length, ignoring any regional differentiation) being

$$Q=\frac{\delta V_{d(t)}}{\delta t}\propto \frac{(deutosternal\ groove\ radius)^{4}}{{deutosternal\ groove\ length}}\Rightarrow PFRt,$$

where deutosternal groove length is a surrogate for the total fluid path. Call this ratio on the righthand side *PFRt*. Note that the more viscous the fluid is (e.g., prey tissues) the slower its flow than say any excreted water with dissolved solutes. Viscous forces, defined as the resistance to flow, are dominant in laminar flow.

Indeed, the surface area of the deutosternal groove better approximates a regular trapezoid as one passes from denticled ridge to ridge along the deutosternal groove (Bowman 1984). Ignoring any induced pressure changes, a better estimate of an overall equivalent uniform pipe radius is $\frac{average\ groove\ width}{2}$ in practice. This assumes that the gap under the tritosternum scales with $groove\ width$ (which is probably slightly overestimating the true cross-sectional area of the ‘pipe’). This simplification does not allow for any possible changes in channel cross-section aspect ratio (Wernz 1972) along its length and the impact of that (Kolliopoulos and Kumar 2021) from species to species. Putting aside this limitation for now, then rather than explicit integration, the numerator ($V_{d}$ in *tep* above) can be estimated grossly over the whole feeding period (for different mites) as being approximately proportional to $V_{p}.\frac{(average\ groove\ width)^{4}}{groove\ length}$ since the feeding time above is assumed proportional to prey size above, the groove ratio term is fixed independent of time and the halving can be lost in the proportionality constant.

Ignoring how many times the same fluid may circulate around the whole ‘masticated prey $\rightarrow$ gut $\rightarrow$ haemocoel $\rightarrow$ coxal secretion $\rightarrow$ circumcapitular reservoir $\rightarrow$ deutosternal groove $\rightarrow$ masticated prey’ system (i.e., effectively fixing the number of times through the pipe as a constant between species), and dropping the constants without loss of generality, leads to defining

$${\hat{tep}}^{*}=\frac{(average\ deutosternal\ groove\ width)^{4}}{(deutosternal\ groove\ length)}.\frac{V_{p}}{V_{m}},$$

where $\hat{}$ indicates estimate. This parameter can be determined from microscopy of specimens or from scaled drawings of each species.

For any particular predatory mite species aimed at consuming tetranychids the second ratio term (in ${\hat{tep}}^{*}$) is essentially fixed. For similar-size candidate BCA species, the first ratio term in ${\hat{tep}}^{*}$ (denoted *PFRt*) dominates. For a given trophic design under proportional linear growth of chitinous structures, ${\hat{tep}}^{*}$ should scale with $IL^{3}$ and thus ${\hat{tep}}^{*}\propto V_{p}$ for that mite’s preferred typical prey consiliently in that case. Hypostome length, subcapitular length and cheliceral reach (*CLI*; Bowman 2020) are all expected to correlate well with $deutosternal\ groove\ length$ (given chelicerae must be retracted into the idiosoma at least enough for food to be delivered to the labrum), so the likely key evolutionary parameter amongst mites of similar reach (or similar relative reach—$\frac{CLI}{IL}$ Bowman 2020) is the average deutosternal groove width (*AGW*) and the corresponding *IL* or *basis gnathosomatica* width (*BGW*) scaled relative widths (i.e., $\tfrac{AGW}{IL}$ or $\tfrac{AGW}{BGW}$ respectively).

For repeat feeding on $i=1,\ldots ,n$ prey individuals, the above argued logic applies for each prey individual attacked. Furthermore, one wants $\sum _{i=1}^{n}V_{m[i]}$ (which $=\varDelta V_{m}$) to be high to ensure the largest number of prey consumed before predator satiation. But the latter term under constant proportionality (see above) can be considered as $V_{m}$ which in turn $=idiosomal\ volume$ (and also approximately equals $\sum _{i=1}^{n}V_{p[i]}$—see above). Now, one wants *n* to be big for an effective predatory BCA. Given similar volume prey, then this resolves to the trivial common-sense result that the mesostigmatid mite $\frac{idiosomal\ volume}{V_{p[i]}}$ should be big, i.e., the relative sizes of predator to each prey should favour the BCA as much as practically possible (while still ensuring trophic access on the plant, say by idiosomal elongation as in soil-dwelling rhodacarids). The inverse of this ratio (i.e., prey dilution into the predator) is the second term in the ${\hat{tep}}^{*}$ equation above.

*PFRt* is taken to be a better measure of the hydrodynamic function of the deutosternum versus the static measure of deutosternal volume (i.e., $DV=GL*AGW^{2}$) to reframe the observations by Wernz (1972). Now, the units of the first term in ${\hat{tep}}^{*}$ is $\upmu$m$^{3}$. So, one could consider this trophic parameter to indicate a ‘pipe’ volume relative to how many times one can drop a prey volume into the predator body volume (i.e., divided by the dilution $\frac{V_{m}}{V_{p}}$). If one arbitrarily sets the relative volumes of phytoseiids and tetranychids $=1$ as a benchmark, then to maintain the same ${\hat{tep}}^{*}$ as phytoseiids (with a deutosternal width $\approx 7\ \upmu$m) by a candidate BCA of say $\frac{V_{m}}{V_{p}}=2$, the deutosternal width would need to be multiplied by $\root 3 \of {2}$ etc., i.e., $\approx \ 9\ \upmu$m (note the diminishing return). This factor resolves to the ratio of *IL* between predator species. So, voracity against the same prey would be synergised by relative deutosternal width (*AGW*/*IL*) between species scaling faster than the corresponding ratio of predator body sizes (i.e., positive allometry over species, or marked excessive relative groove widening).

### Micro-structural considerations

The above derivation describes a fluid pipe. However, what causes the deutosternal groove to fill and prey-related liquids to flow? Of course, the pressure difference ($\varDelta p$ above) may be externally driven by forces from pharyngeal pumping, gnathosomal or cheliceral movements, etc., but at the expected $10{-}100\ \upmu$m scale of a deutosternum/tritosternum ‘pipe’, capillary action by spontaneous wicking of liquids in narrow spaces also comes into play. Wernz (1972) describes exactly this. Indeed the primary structure of the deutosternal groove on its own (ignoring any edge ‘nano’-structure) is like a horizontal open rectangular cross-section microchannel used in capillary microfluidic technologies (Berthier et al. 2019, Kolliopoulos and Kumar 2021).

As Wernz and Krantz (1976) say, the deutosternal “... fluid layer is extremely thin”. A microchannel is defined as a narrow channel with dimensions ranging between 1 $\upmu$m and 1 mm (Tan et al. 2020). Microfluidic devices are defined as those in which at least one of the dimensions is smaller than a millimetre (Williams et al. 2008). At small scale, surface forces dominate fluid behaviour—beyond 1 mm, the fluid flow behaves similar to that of macroscopic flow. Note that the larger pergamasids are rapidly moving hunters around 1 mm in size overall (Bowman 2017). Size matters in mites, they are in a hydrodynamic ‘Goldilocks zone’. At the start of microfluidic flow (i.e., channel imbibition) fluid inertial effects dominate, at later stages viscous effects dominate. Passive unidirectional flow without external pumping can occur (Comanns et al. 2015). In these channels, capillary action with a characteristic concave front surface (or ‘meniscus’), occurs when the fluid’s adhesion to the walls is stronger than the cohesive forces between the polar imperfectly wetting liquid’s molecules. This is the same attraction responsible for surface tension which causes the leading molecules of a fluid to pull the neighbouring liquid molecules along behind them and make characteristic symmetric droplets of minimal surface area on hydrophobic surfaces. Complete removal of the tritosternum is assumed by acarologists to switch off the subcapitular pipe by breaking the link to the circumcapitular area. However, even if the tritosternum is removed or just held ventrally away from the deutosternal groove, the primary structure of the ‘pipe’ could still act as an open microchannel. Here the precise convexity (see Wernz 1972) or concavity of the surface may be important.

Gravity will play a negligible role at this mite-sized scale providing the gnathosoma is held broadly horizontal (i.e., an airorhynchid attack design favoured by larger soil predators; Bowman 2020). Sufficient wettability will retain the fluids from falling out of the deutosternal groove as the open channel is upside down with respect to the upper plant surface that a tetranychid prey mite is standing on. Ignoring evaporation, the capillary pressure gradient ($\varDelta p_{c}$) is caused by the mobility of the circular-arc meniscus with its characteristic equilibrium contact angle ($\theta$) dependent upon the fluid’s composition. This mobility parameter can be thought of as a diffusion coefficient driving the spontaneous growth of the liquid interface. Thus fluids may spontaneously flow (as Wernz 1972 describes) in this open microchannel (without the need for pharyngeal pumping as the moving force). A detailed theoretical analysis of flow including evaporation in open rectangular microchannels is available in Kolliopoulos and Kumar (2022).

**Fig. 3:**
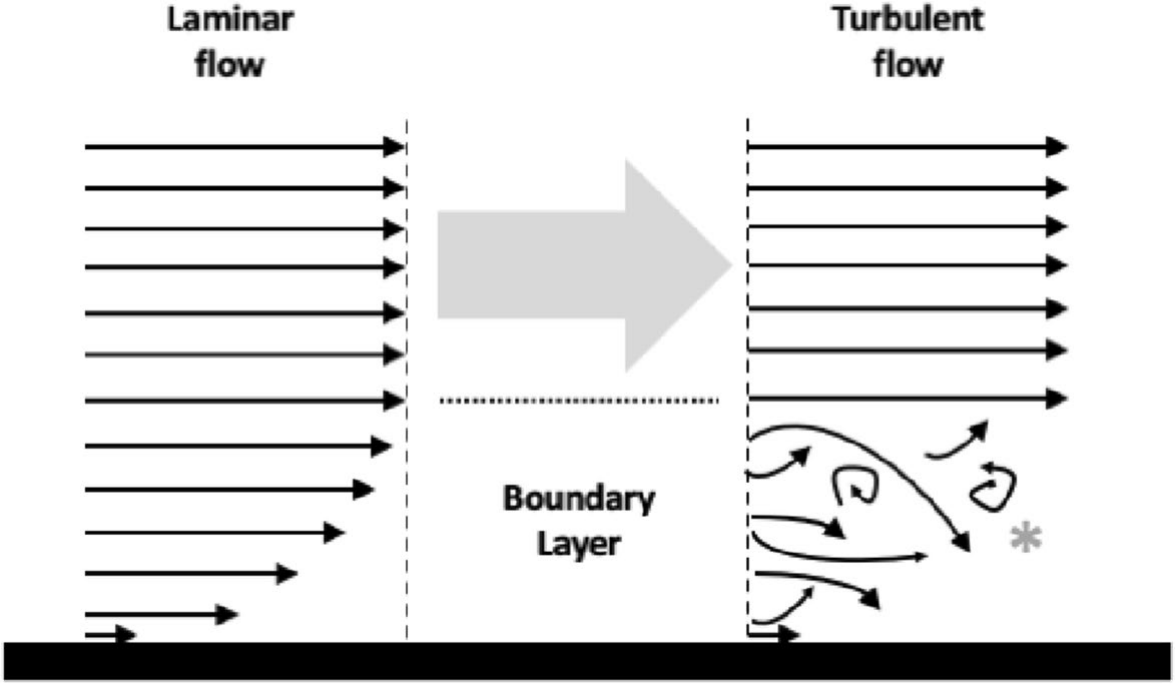
Schema of stream-wise velocity flows (black arrows) over deutosternal surface (black layer), here shown smooth but could be rough with ridges. Surface viscosity drives flow resistance, such that velocity at surface can be zero (no slip), Grey arrow is free stream above boundary layer. Laminar flow (left) can basally break into unsteady turbulent disordered flow (right, grey *) as flows increase which can in turn become separated. Turbulent vortices encourage mixing. A viscous sublayer can remain tight to the surface (lowest small arrow)

This study herein is based upon assuming laminar (i.e., layered) flow even over regions of rough surfaces, i.e., even the boundary layer of flow in any pipe and/or channel is assumed to be (initially) laminar (Fig. 3 left hand side).

As fluid moves past the groove/pipe walls, the molecules right next to the surface stick to the surface. The molecules just above this are slowed down in their collisions with the molecules sticking to the surface. These molecules in turn slow down the flow just above them, and so on. Thus the farther one moves away from the subcapitular surface, the fewer collisions are effected. This creates a thin layer of fluid near the surface in which the velocity changes from almost zero at the surface to the free stream value away from the surface. This is the boundary layer, which itself can become turbulent given a high enough Reynolds number ($\equiv$ ratio of the inertial forces to the viscous forces) and further affect skin friction drag. Kadivar et al. (2021) is an entry point to the literature on turbulent flow and rough surfaces (other papers could be chosen). The thickness of the boundary layer depends upon that fluid’s Reynolds number. Although sharks, rays (De Melo et al. 2022) and other aquatic animals do have surface spicules and riblets on their skin to improve hydrodynamics overall (Lauder et al. 2016) and in turbulent flow regimes (Dean and Bhushan 2010), drag is a multi-scale problem (Adams and Zamprion 2020). Herein drag is taken to be only surface-friction drag (Bushnell and Moore 1991) between the fluid and the mite cuticle.

For sure, some micro-channel surface roughness reduces flow (Xing et al. 2020). So such cuticular roughness on the sub-labral keel and oral gutter in predatory mites may restrict (not facilitate) prey fluid flow as well as masticate material by their opposing action on labral movement. There is no need to pose a straining function (Evans and Loots 1975; Evans 1992). It certainly would be a very small mesh to pass through if the latter was its function.

Some surface features decrease drag (Yu et al. 2020). Does the surface spiculate roughness seen throughout the length of the deutosternum in *Crassicheles holsaticus* Evans (1980), thus facilitate or impede flow? How might the denticle fields illustrated by Hirschmann (1962) in the uropodoids *Uropoda cassidea* and *Uropoda virgata* function? As is said,“the devil is in the detail". Indeed, wettability gradients along the channel surfaces can, counterintuitively, drive and accelerate overall liquid flow (Xing et al. 2020) even making water spontaneously move uphill. Hemi-wicking or super-wetting (Varaday and Mantooth 2021), a mechanism whereby due to capillary action a liquid wets a textured rough hydrophilic (Kim et al. 2016) otherwise impenetrable surface beyond its intrinsic wetting length (Krishnan et al. 2019) may also come into play. Here liquid wicks between the micro-features within rough effectively porous wall structures enhancing the capillary pull. Is this the function of sub-labral spicules, to synergise pharyngeal pumping? Whilst the general surface of the deutosternal groove appears smooth in SEM pictures of mesostigmatids, the secondary structure is rough at a certain scale. The mechanism by which such fluids spread in acarine deutosternal channels therefore needs careful consideration.

For materials like a hypostome or any subcapitulum with a highly irregular surface topography, the wetting front is expected to be diffuse and as such deriving analytical spreading model parameters directly from a mite’s surface topography would not generally be possible. Heuristics come into play. Small pillar patterned surfaces can facilitate droplet spread (Chen et al. 2019). This may be how the anal cribum spreads secretions in mesostigmatids (e.g., blattisocids; Lindquist and Moraza 2016) rather than repelling liquids like the surfaces of some plants (Quéré 2002). Other features will be relevant too to deutosternal liquid dynamics including dissolved solutes such as digestive emulsifying surfactants (Bowman 2019) and prey proteins in overspills or coxally excreted salts, sugars, etc., changing the fluid’s surface tension. Of course, having a fully open-top ‘pipe’ by the lifting of the tritosternum away from the venter of the hypostome reduces the risk of clogging the micro-channel with debris carried along by moving liquid solutions or colloidal suspensions.

Ridges on the usually hydrophobic chitinous shields of arthropods are assumed to hold any liquid deposits away from fully wetting any planar chitinous surfaces by virtue of surface tension in the droplets of fluids. For sure barriers, like a human hair at approximately $70\ \upmu$m thick, will hold back a substantial water droplet on a smooth surface. At least pergamasids are known to manipulate droplets bridged like this with their legs/gnathosoma (Bowman 2014) onto the substrate as discards. The height of the deutosternal primary and secondary structure may be just another such tool allowing flow to first be pinned, i.e., ‘stick’ at the surface features, then deform and ‘slip’ as pumping pressures or volumes increase.

Groove cross-sectional shape matters in microchannel fluidics. Defining a channel’s aspect ratio as

$$\lambda =\frac{channel\ height}{channel\ width}$$

then

- a large $\lambda$ groove channel behaves more like a modified Lucas–Washburn model of a simple fluid pipe;
- a small $\lambda$ groove channel behaves more like filament-propagation using a lubrication theory-based model of fluid flow.

Considering for instance mites with the same deutosternal channel depth, this again points to the importance of groove width (*AGW*) in determining its fluid operating characteristics. However, given mites of the same deutosternal width then groove height (perhaps measurable on SEM micrographs) would be crucial in determining fluid microdynamics.

Large heights induce pipe-like flows. However, for small height, wide deutosternal grooves, the additional contribution of free-surface curvature to liquid capillary flow in open rectangular cross-section micro-channels can lead to the formation over time of very thin filaments or ‘fingers’ extending in advance of the meniscus front along the four edges of the channel and influence its propagation (Kolliopoulos and Kumar 2021). These can extend essentially with no limit (Ouali et al. 2013) and even completely exit the end of the channel. Their increasing prominences could potentially spread ‘contaminants’ between micro-compartments that would otherwise be separate areas of liquids. Subtle integrated control of the configuration of mite body parts (e.g., labrum, internal malae, etc.) is thus needed if these type of fluid dynamics occurs, e.g., when any pumping is not occurring. This is true of fluid-feeding insects (Silva and Grunewald 2000) as much as in mites. Hard edges matter at this scale and acarologists are used to taking notice of them on various chitinous structures and drawing them in detail. Initial attempts at a classification of deutosternal forms has been made (Bourdeau-Gorirossi 1989). However, they are not just taxonomic characters (e.g., Hirschmann 1962; Karg 1965) but have a real hydrodynamic function.

**Fig. 4:**
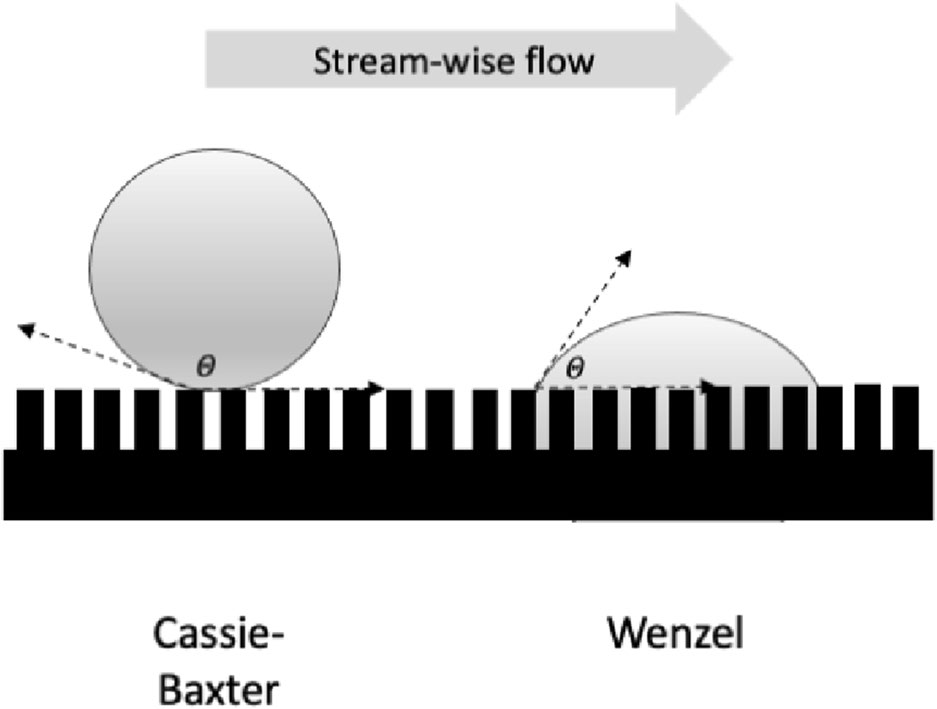
Schema of fluid droplets on an acarine cuticular surface with a slotted geometry of rectangular span-wise ridges—see Nguyen et al. (2014) and Bello et al. (2023). Spreading wetted Wenzel state (right) equilibrium contact angle $\theta < 90^{\circ }$ and hydrophobic Cassie–Baxter repelling state (left) contact angle $\theta > 90^{\circ }$. Cassie–Baxter droplets can slide over the surface (Tian et al. 2022). *Note* droplets and span-wise surface texture beneath them are not drawn to scale, also many more transverse ridges illustrated than typical for a mesostigmatid deutosternum. *k*- or *D*-type surface roughness can induce turbulence in moving Wenzel state fluids depending upon inter-rib distance (Han et al. 2021)

Despite almost 100 years of investigation in insects (Holdgate 1955), wetting is a complex topic. Droplets that sit on a surface are described as being in the Cassie state, those that are homogeneously wetted to the surface are in the Wenzel state (Hensel et al. 2016, Jin et al. 2020)—see Fig. 4. The equilibrium contact angle $\theta$ of the liquid is crucial in determining adhesion to any surface (Gells et al. 2019). A simple explanation of how surface topography and chemistry affect surface hydrophilicity and hydrophobicity can be found in Nguyen et al. (2014). However, the coating of a rough solid by a liquid can preserve the roughness if the film is thin enough (Quéré 2002). So, it is tempting to conclude that fluid flow in such a small $\lambda$ deutosternal groove design with thicker fluid prominences (Ouali et al. 2013) could be facilitated by having denticles of just the right size on its cross-ridges (as in mites). The same would apply to any partly opposable plumose or very fimbriate tritosternum resting nearby along the groove (or its nano-folded edges?) offering many more edges for fluid to creep along. Such in-channel structures would augment the solid–liquid contact area and hence increase the capillary pull ($\varDelta p_{c}$). Gradually opposing the tritosternum dorsally upwards to a horizontal position would encourage the circum-gnathosomal groove prey/coxal fluids to step slowly up from deutosternal ridge to ridge until a full continuous pipe is formed up to the fluid ‘ring’ described by Wernz and Krantz (1976) encompassing the hypostomal tip. Any multiple tips to the tritosternal lacinae then ensuring a distribution of fluid streams up to final steering by the various hypostomal tip excrescences (= internal *malae* often observed in mesostigmatids). This tritosternal control (or ‘switch’) would allow the mite to move modest amounts of material along the channel without any pumping, yet switch to bulk pipe-based flow in order to pump large volumes of recyclate back into the gut when necessary perhaps driven by the labrum pre-oral gutter and pharynx opening and closing (out of phase as in the buccal-opercula double pump of fish gills). For sure, in tritosternumectomised parasitid mites, the increasing volume of subcapitular suture fluids (presumably from the coxal glands shipping watery liquids out from the idiosomal interior; Bowman 2014) can occasionally engulf the tritosternal base, repeatedly grow, break and run down the mite’s ventral surface or be lost into the coxal angles formed by the (leg) coxae and the venter with accompanying gnathosomal ventral bending (Wernz 1972). The latter has some commonality with the normal persistent coxal droplets and observed ‘tilting discards’ in pergamasids feeding on very large prey recorded in Bowman (2014). Note that in tritosternumectomised mites, hypostomal fluids still disappear immediately (by imbibition) on the discard of the mite carcass. Perhaps macrochelids as well as parasitids might also produce macroscopically visible coxal droplets during normal predation given appropriate humidity conditions and excessively watery large prey? More investigation is needed.

The advantage of any water repellent surface is the ability in minimising the flow resistance in order to gain higher mass flow rates in narrow media such as any deutosternal microtube or channel when the external power source is restricted (Tan et al. 2020). As mesostigmatids with shallow facultatively open deutosternal grooves have different numbers of ridges across them (phytoseiids have $\approx 7$; Flechtmann and McMurtry 1992b; Liu et al. 2017), one interesting potentially trophically important feature to also evaluate is the inter-Querleisten spacing (or $wave\ length$ of denticled ridges) over the mites of different sizes (in phytoseiids over the three life-style groupings this is $\approx 6.7\ \upmu$m; Liu et al. 2017). This could inform the size (and therefore the physics; Berthier and Brakke 2012) of any discrete droplet of fluid sitting or moving on the subcapitular hydrophobic (or superhydrophobic; Yu et al. 2018) surface. Further to record

- if the shape and orientation of the ridges match a concave meniscus front with respect to the posited direction of fluid flow, and
- to note the anterior–posterior or posterior–anterior direction of the denticles on each ridge, and
- to record the number of denticles on each ridge ($\approx 2$ over the three phytoseiid life-style groupings; Liu et al. 2017).

These measures should inform the interpretation of function and help explain the observation by Wernz (1972) of a ‘hypostomal droplet’ anteriorly. Future work could investigate patterns of hydrophobic surfaces and hydrophilic paths (Darhuber et al. 2001) over all the mite gnathosoma. Furthermore perhaps SEM studies could attempt to measure comparatively the deutosternal groove depth between mites to check conclusions.

### Proposal

From all of the above, one concludes that:

*A suitable candidate BCA for biological trials may be a not too large active mesostigmatid of good reach compared to tetranychid sizes. It should have a potentially large deutosternal ‘pipe flow’ design with particular specialists having a denticulated deutosternal groove of notable average width relative to their size*.

This pilot feasibility study tests this.

## Materials and methods

**Table:** Glossary of abbreviations

Abbreviation	Meaning
$\varDelta$	“Change in...” (e.g., change in volume $\varDelta V$)
$\delta$	“Infinitesimal change in...” (e.g., infinitesimal change in time $\delta t$)
$\lambda$	Microchannel depth to width aspect ratio
$\omega$	Deutosternal wavelength between transverse cross-bars (‘Querleisten’)
$\varTheta$ or $\theta$	Equilibrium contact angle of liquid
*A*	Circumcapitular groove volume
*B*	Volume of single cheliceral protrusion or retraction (‘Tubevolume’)
*AGW*	Average deutosternal groove width (without lateral extensions)
BCA	Biological control agent
*BGL*	*Basis gnathosomatica* length
*BGW*	*Basis gnathosomatica* width
*BSL*	Basal cheliceral segment length
*CHI*	Cheliceral height index
*CLI*	Reach or cheliceral length index
*CSL*	Length of cheliceral segments
*DG*	Deutosternal gulp ($=DV$)
*DSL*	Distal cheliceral segment length
*DV*	Deutosternal volume
$f_{1}$	Characteristic resonance frequency
*F*1	Chelal adductive input lever arm muscular force
*F*2	Occluding chelal crunch force (*F*2*AV* in Bowman 2020)
*GL*	Deutosternal groove length
*GW*	Width of gnathosoma (see Bowman 2020)
*IL*	Idiosomal length index
*MDL*	Moveable digit length
$NIR_{t}$	Net imbibition rate at time *t*
*p*	Pressure
*PCn*	*n*th principal component
$PFR_{t}$	Deutosternal pipe flow rate
*r*	Correlation coefficient
$R^{2}$	Percentage variation explained
${\hat{tep}}^{*}$	Estimated trophic efficiency parameter
*t*	Time
$V_{d}$	Total volume of deutosternal + circumcapitular groove assembly
$V_{i}$	Ingestion volume
$V_{m}$	Volume of (predatory) mite
$V_{p}$	Volume of prey
*VR*	Adductive moveable digit lever arm velocity ratio $\frac{L1U}{L2M}$ (Bowman 2020)

Detailed nomenclature of deutosternal transverse cross-bars (‘Querleisten’) follows (Hirschmann 1962). Cheliceral measures follow Bowman (2020)

Scaled illustrations of a variety of mesostigmatid species from recent acarological papers were sourced by informed ad hoc Google searches of the Internet ensuring a wide variety of gamasine families were covered. Females of 72 species were digitised (Table 1) as in Ujvári (2011). These were analysed using ImageJ 1.53k ex National Institutes of Health USA (http://imagej.nih.gov.uk/ij). Structures (see Fig. 1) were measured and design parameters (e.g., ${\hat{tep}}^{*}$) estimated using data values and conversion formulae, e.g., in Tables within Bowman (2020) etc., if necessary. By convention, the posterior of the hypostome is demarked by the insertion of the posterior-most ($h_{2}-h_{3}$) setae. There are some exceptions in mites to this rule, e.g., Fig. 12.19C in Krantz and Walter (2009) (Dave Walter *pers. comm.*). The average deutosternal groove width was estimated by the planimetric area of an irregular polygon encompassing the deutosternum divided by the groove length. Tetranychid idiosomal length index (IL) was estimated from an average over species from scale drawings of adult female: *Tetranychus afridinicus*, *Tetranychus arifi*, *Tetranychus ismailis*, *Tetranychus salicornis*, *Tetranychus papayae* and *Tetranychus zaheri* in Carlos Flechtmann’s (2022) unpublished identification summary of the genus *Tetranychus* as per Bowman (2020) rather than Saito et al. (1999).

The hat notation (e.g., ${\hat{A}}$) is used to indicate observed estimates (of *A*) derived from the data. Linear regressions are through the origin unless otherwise stated. For a definition of terms used in describing the properties of surfaces see Law (2014).

Where possible the number of denticles on each ridge of the deutosternum were determined. An average between the minimum and maximum was then used for that species. For some species denticles could only be qualitatively described as few, some, many, or lots. For the purposes of comparative illustration, across all species studied, these categories were quantitatively mapped heuristically to 2.5, 5, 15 and 25 denticles, respectively.

Mesostigmatid mite gnathosomas were measured (*CLI* = reach = $DSL+BSL$, *MDL* = gape, $F1=CHI^{2}$, chelal ‘crunch force’ $F2=VR.F1$, etc.) and characterised as micro-, meso- and mega-cephalic as per Bowman (2020). Overall cheliceral segment length was the sum of the segment lengths minus the moveable digit length, i.e., $CSL=CLI-MDL$ (any baso-basal 3rd cheliceral segments if present were ignored throughout).

**Fig. 5:**
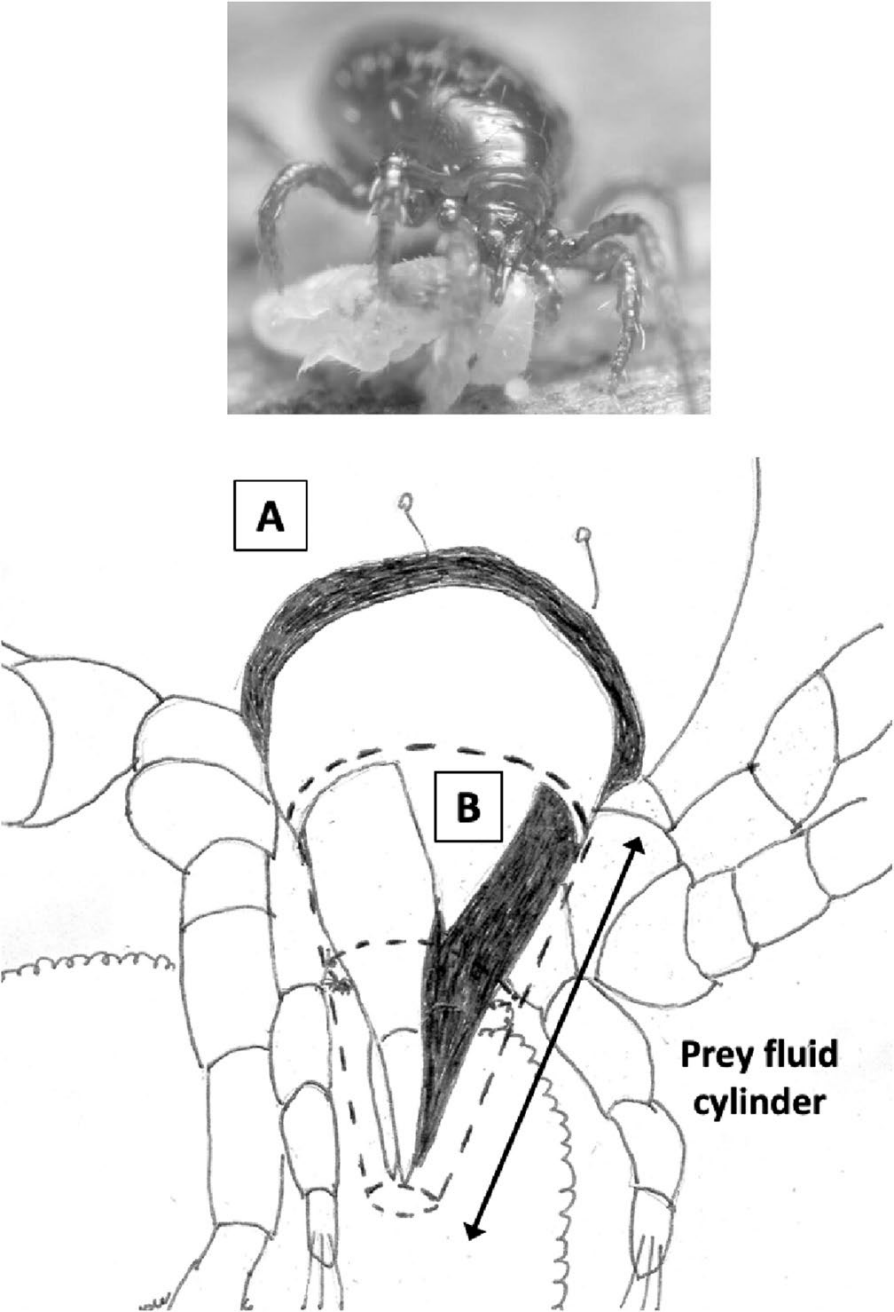
Fluid compartments in the gnathosoma of a predatory mesostigmatid (dorsal view) with chelicerae extended towards viewer. A cylinder of fluid encompassing the cheliceral module between the inner flanks of the setose palps (here apparently conical in shape simply because it is plunging down away from the viewer into the larval prey) connects the proximal gnathotectum/hypostome edge (dashed) to prey tissues being masticated by the cheliceral chelae. *Note* the basal cheliceral segments rest above where the hypostome may protrude ventrally, distal cheliceral segments approximate the length of gnathotectum, and the chelae rest at the level of the palp tips. (A) Circumcapitular groove full of overspilled or coxal fluids (estimated as $\propto BGW.(AGW^{2})$ and coloured black). (B) Volume available for prey fluids on single cheliceral full protrusion/retraction (estimated as $\propto Reach.CHI^{2}$ and coloured black). The posterior edge of (B) marks the end of the fluid ‘ring’ described by Wernz and Krantz (1976). Salivary styli are omitted. Redrawn and amended from colour photograph of Laelapidae. Under plank, Montagu, NW Tasmania, Nov 2014 $\copyright$ Andy Murray/chaosofdelight.org with permission

**Fig. 6:**
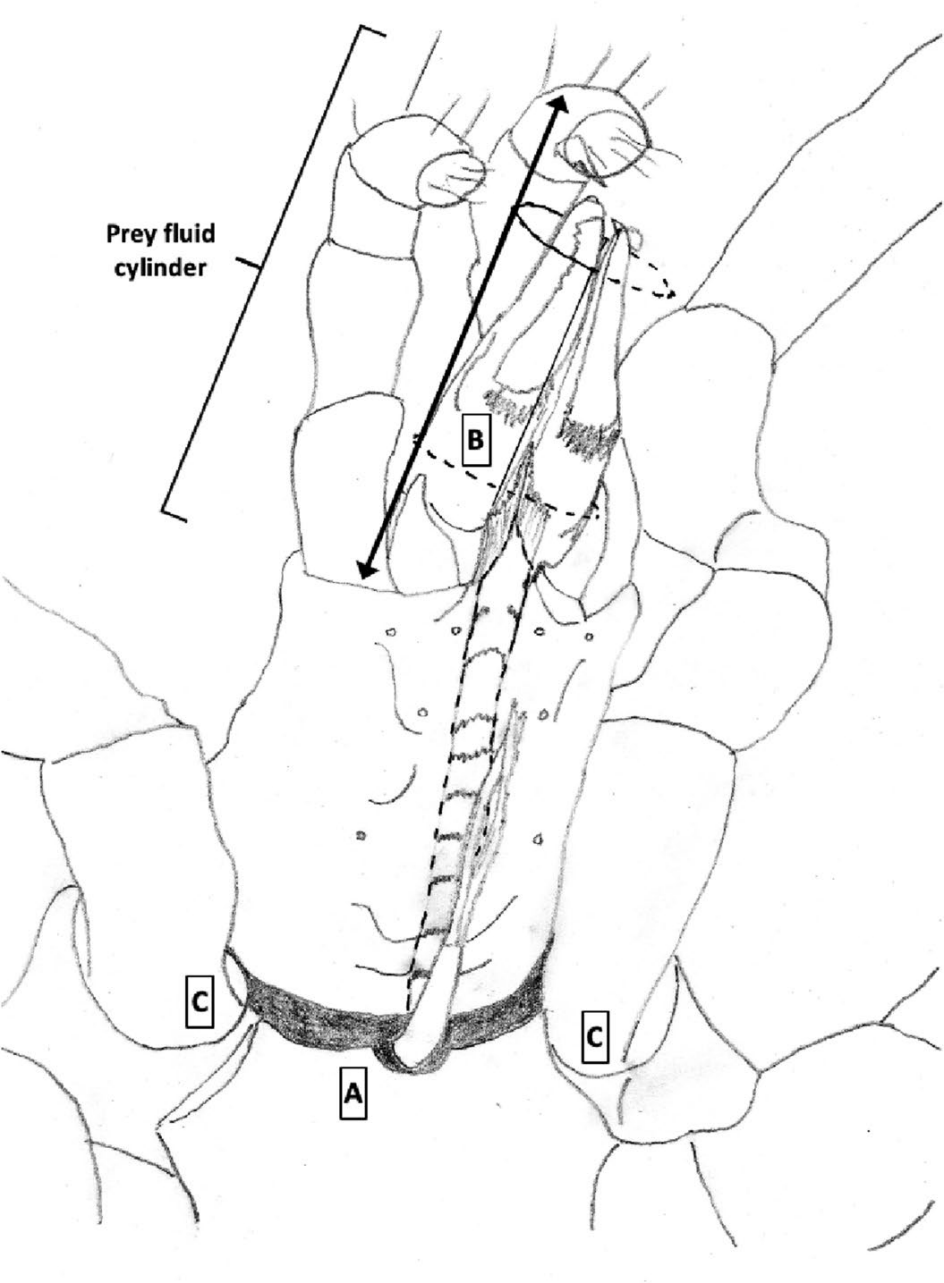
Fluid compartments in the gnathosoma of a predatory mesostigmatid (ventral view) with chelicerae partly retracted. A ring-like cylinder of fluid encompassing the cheliceral module between the inner flanks of the palps connects the gnathotectum/hypostome to prey tissues being masticated by the cheliceral chelae (full extended side indicated by double headed arrow). Note cheliceral segments hidden inside subcapitulum. (A) Circumcapitular groove full of overspilled/coxal fluids [estimated as $\propto BGW.(AGW^{2})$ and coloured black]. (B) Volume available for prey fluid on single cheliceral full protrusion/retraction (estimated as $\propto Reach.(CHI^{2})$)—not coloured in black so as to visualise cheliceral structure. Posterior edge of prey fluid cylinder matches that of the fluid ‘ring’ described in Wernz (1972) and Wernz and Krantz (1976). (C) Area of debouchment of coxal glands. Deutosternal groove indicated by dashed outline here extending to bifurcate tip of hypostomal module (deutosternal volume estimated by $\propto AGW^{2}.GL$). Semicircular full or part ridges (= Querleisten; Hirschmann 1962) with anterior facing denticles traverse the groove regularly. Other ventral surface ridges indicated. Note fimbriate internal malae centrally on the hypostome and arthrodial brushes to the base of chelal joints. Salivary styli are omitted. Drawn and amended from photograph of *Antennolaelaps* gnathosoma ex Photon Challenge: Last Chance (March 19, 2011) by Dave Walter at https://macromite.wordpress.com/page/4/ with permission

Various estimates of fluid volumes were made. The *basis gnathosomatica* width (*BGW*) was taken to be the maximum width of the gnathosoma. Then, the circumcapitular groove volume (A) was estimated as being $\propto BGW.(AGW)^{2}$ (Fig. 5), i.e., a length equivalent to the gnathosomal cross-section perimeter and a square groove width and depth around the outside sides of the gnathosoma equivalent to *AGW*. This is regarded as a buffer store. It was described as the postcapitular suture by Wernz (1972) and the postcapitular channel by Wernz and Krantz (1976). It majorly functions during pergamasid feeding (Bowman 2014) where no sternal overflow was observed. The volume displaced by a single chelicera on protrusion/retraction (B) within the gnathosoma was estimated as being $\propto Reach.(CHI^{2})$ (Fig. 6). This is often abbreviated herein to the term ‘Tubevolume’ and is considered to be the primary fluid challenge for the mite to deal with. Being a gnathosomal cross-section extruded out to the mite’s reach, it is an approximate multiple of the volume of the fluid ‘ring’ described by Wernz and Krantz (1976), i.e., the “copious flow of prey fluids around and between the chelicerae” ....“posteriorly to the epistome” on prey rupture, the gnathosoma being “inserted into its prey nearly to the level of the palptrochanteral angles”. The ring encompasses the internal malae and the bases of the corniculi. The deutosternal volume (described in lateral view as “a fluid bridge” by Wernz and Krantz 1976) was estimated as being $\propto GL.(AGW^{2})$, i.e., a cylindrical tube. This is sometimes referred to herein as ‘deutosternal gulp’ (*DG*). It in itself is not regarded a priori as a major buffer store.

The observed deutosternal ridge wavelength on average was estimated by *GL* divided by the ‘*number of ridges including both of those actually or notionally at the ends*’ minus 1. This is considered as representative of peak-to-peak distance between ridges.

## Results

The intention in this review was to cast its net deliberately wide. Of interest was the hydrodynamic differences of phytoseiids from a general mesostigmatid ‘Bauplan’. Various modern acarological authors’ works were examined. This study was designed to give on average four exemplar species per mesostigmatid family. Their representativeness as being typical of their family was ensured by the random collection of these species from different publications. A wider follow-up study of more species could explicitly test this.

Deutosternal architecture varied considerably over the species reviewed herein. Most species showed the classic deutosternal groove of two longitudinal edges and multiple transverse cross ridges. These longitudinal edges could be considered as equivalent to two of the stream-wise riblets found on the placoid scale of shark-skin denticles. Here their cross-section matters in determining fluid drag reduction (Pu et al. 2016) and this needs examining in a follow-up study of mites. Are any of the ridges (stream-wise or span-wise) blade-like, sawtooth or scalloped which will affect their hydrodynamic properties (Tian et al. 2021)? However, *Philippinozercon makilingensis* showed no cross ridges at all and the megisthanids showed an overall tessellated structure with a central suture. A central suture with cross-ridges was present in *Heteroparasitus mariae* and most of the davacarids examined. *Davacarus lindquisti* lacked transverse ridges. *Acanthodavacarus klompeni* lacked longitudinal edges (these being also missing in *Iphidozercon gibbus* and *Sejus serratus*; Karg 1965). Yet these mites must handle fluids appropriately.

The demarcation of the posterior of deutosternum length can be not so clear, e.g., Fig. 12.19C in Krantz and Walter (2009), following the approach illustrated in Fig. 5d of Evans and Till (1979) is far better (Evert Lindquist *pers. comm.*). Herein the most posterior extent of the longitudinal ridges is used (with a notional ‘closing’ ridge if short of the circumcapitular groove, e.g., *Leioseius naglitschi*, *Antennoseius pannonicus*, *Eugamasus nolli*, *Hypoaspis auris* and others; Karg 1965), or the circumcapitular groove itself if the ridges appears to plunge into it (like *Arctoseius sessiluncus* or *Antennoseius dungeri*; Karg 1965). Also, the posterior of the hypostome being defined by the insertion of the posterior-most $h_{2}-h_{3}$ was not always clear (so the palp trochanter is used herein; Fig. 1). The level of the setal insertions of $h_{2}-h_{3}$ (when transversely aligned) does not always mark the anterior of the deutosternum either. Of course, in published illustrations the insertion locations of hypostomal setae may be stylised. Alignments may not actually be correct and other pairs of structures anterior of the first transverse row of deutosternal denticles, at the level of the cornicular bases may exist. Accordingly, this review focuses on the longitudinal ridges as being the key defining measure (allowing the deutosternum to extend deep, and being thus *integrated*, into the hypostome if necessary). Best endeavours were used to define the anteriormost transverse ridge characteristics which may then be a smooth (denticles-free) margin (Evert Lindquist *pers. comm.*) or essentially notional (i.e., when there is no physical ridge where the longitudinal edges end). It is acknowledged that there is an element of subjective judgement here in the location of the ‘closure’ at the front of the groove (see discussion on sutures below). Notwithstanding this, for the five families in common between this review and Wernz (1972), i.e., Eviphididae, Heterozerconidae, Laelapidae, Macrochelidae and Phytoseiidae, the average length of the deutosternal grooves generally agree ($R^{2}=0.9122$), as does *BGL* with Wernz’s length of the gnathosoma from base to hypostomal seta $h_{3}$ ($R^{2}=0.8758$), pointing to the reasonable typicality of both studies.

Using groove width (*AGW*) in the estimation of circumcapitular groove buffer volume is reasonable given that Wernz (1972) states that the depth of the subcapitular suture fluid “...seldom exceeded the thickness of the tritosternal base...”. Further that “..the tritosternal base seemed to stop the fluid from rising too far, and spilling or running all over the venter of the mite..”. However, herein the deutosternal groove volume in itself is not considered just to be a static buffer store (as assumed in Wernz and Krantz 1976) rather it is seen a dynamic fluid bridge between gnathosomal fluid compartments. Normally it vanishes after feeding (Wernz 1972).

### Comparability of species studied

**Fig. 7:**
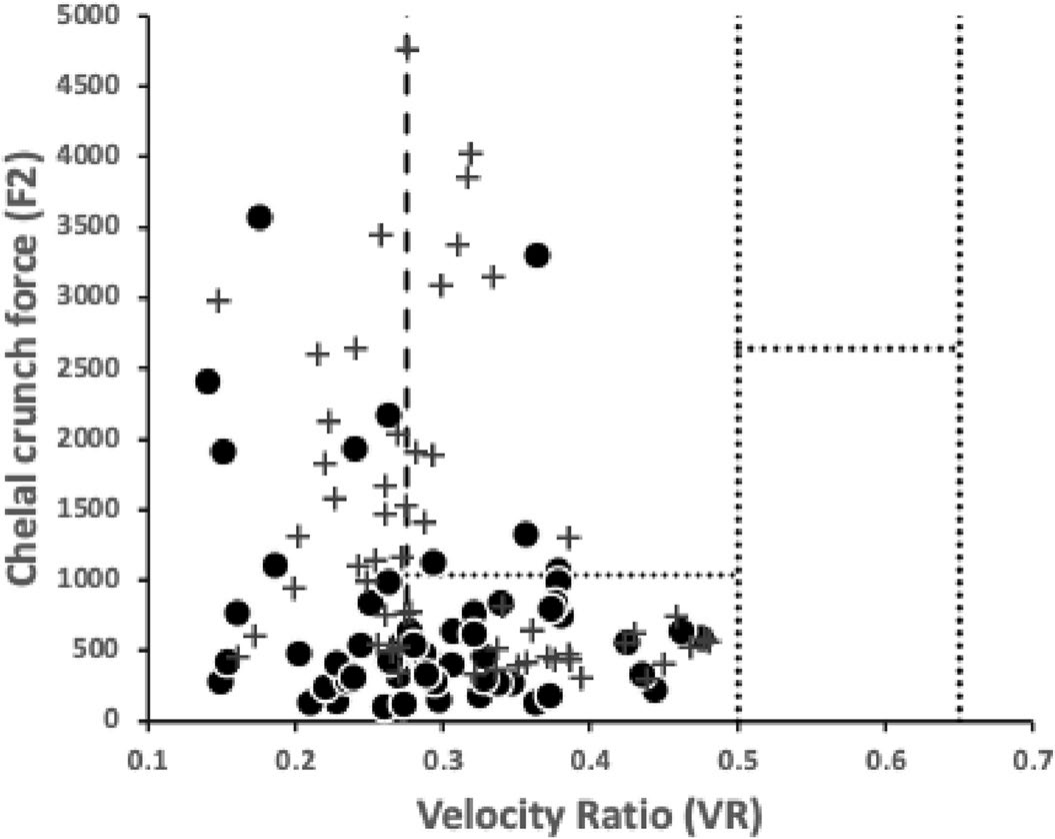
Plot of species used (including *Uropoda abantica*) in the space of chelal velocity ratio (*VR*) and chelal occlusive crunch force (*F*2) showing the similarity of the species studied herein (black dots) with those of Bowman (2020) (shown as pluses). Boundary at $VR=0.276$ indicates threshold between arthropod-cutting style feeding action (to the left) and worm-crushing style feeding action to the right. Hypocarnivory indicated below lowest dashed horizontal boundary ($F2 \approx 1000$). Other boundaries demark regions of astigmatid and oribatid cheliceral designs (see Bowman 2021)

A variety of sizes of mites (measured by *IL*) was deliberately chosen for this review. Figure 7 illustrates that the species used in this pilot study, although being different to, still strongly overlap the cheliceral design space of those studied in Bowman (2020).

**Fig. 8:**
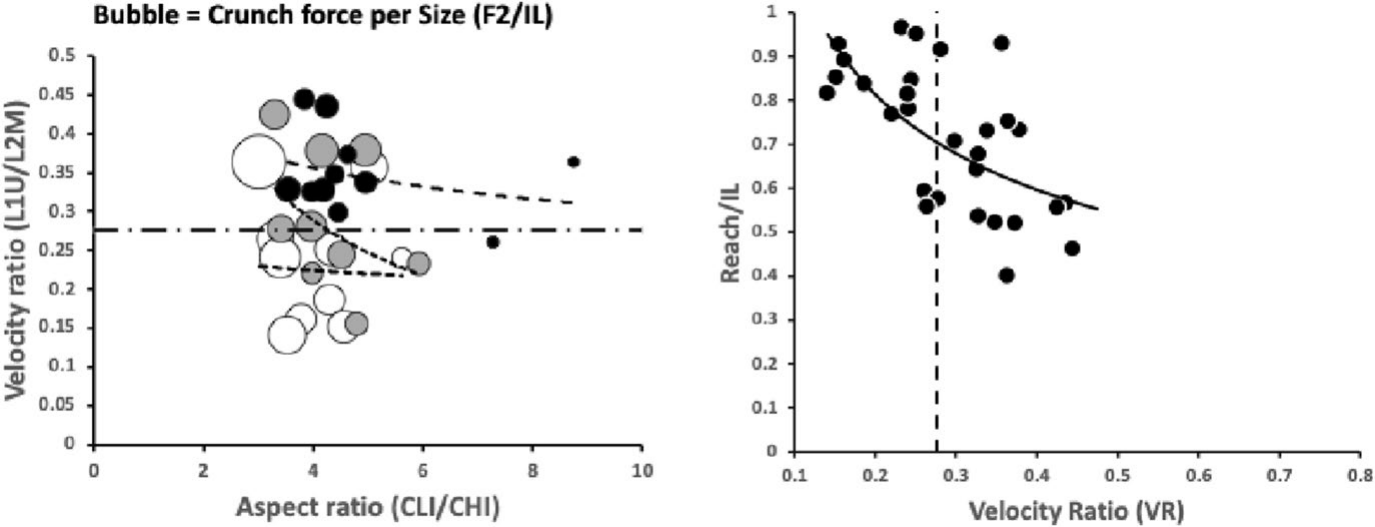
Plot of species studied (including *Uropoda abantica*). *Left* in the space of chelal velocity ratio (*VR*), size-adjusted chelal crunch force and cheliceral aspect ratio for micro-cephalic (black dots), meso-cephalic (grey dots) and mega-cephalic (white dots) species together with power trend lines consilient with results for other free-living mesostigmatids in Fig. 25 of Bowman (2020). Note comparative lack of megacephalic large (7–8) aspect ratio exemplars. Horizontal threshold at $VR=0.276$. *Right* large and small mites have different constraints. *Black dots* species used in this feasibility study. The dashed vertical line is killing style design threshold. Thin black trend line as power function simply to illustrate effective break-point regression at threshold, between a scaling for one trophism (on the left) and a scaling for the other (on the right). Relative measures on the *y*-axis do not vary much for predicted worm-like prey-crushing feeders (on the right) but show a concerted change versus micro-arthropod prey-cutting style feeders (on the left) just as in Fig. 23 of Bowman (2020)

As in Fig. 23 (right panel) of Bowman (2020), species predicted by velocity ratio to favour micro-arthropod feeding have excessive reach (*CLI*) for their body size; those predicted to be worm-like prey feeders do not (Fig. 8, right panel). Note that reach, i.e., *CLI*, on its own is not a good predictor of feeding on vermiform prey (Bowman 2020). Rather large reach is an indicator of an attack style at long range. It is the chelal velocity ratio that is predictive. *Antennocheles* sp. who have exceedingly excessive reach (Lindquist and Moraza 2014), have chelae indicative of high-speed closing. However, they are known to be nematophagous which is thus not consilient. Perhaps this excessive reach (with possible third cheliceral segment like uropodids, telescopic sheathing internal to the idiosoma, etc.; Evert Lindquist *pers. comm.*) is a special adaptation for probing remotely in fluid films/refugia on enfurled *Heliconia* leaf surfaces as the predator slowly quests? Blind probing while the predator moves with a swimming-like action would require a fast acting grip on prey before it could squirm and swim away. The corollary would be the need for sensing structures on the tip of chelal digits like in uropodids but they are not obviously present (Lindquist and Moraza 2014). By happenstance, fewer diverse taxa with very high *CHI*/*IL* or *F*2/*IL* values were included in the study herein. Few excessive gape species were included in this feasibility study so also validating Fig. 23 (left panel) in Bowman (2020) awaits further work (which should include examining the deutosternum/tritosternum of more ascids and especially antennochelids).

### Proportionality of subcapitular measures

**Fig. 9:**
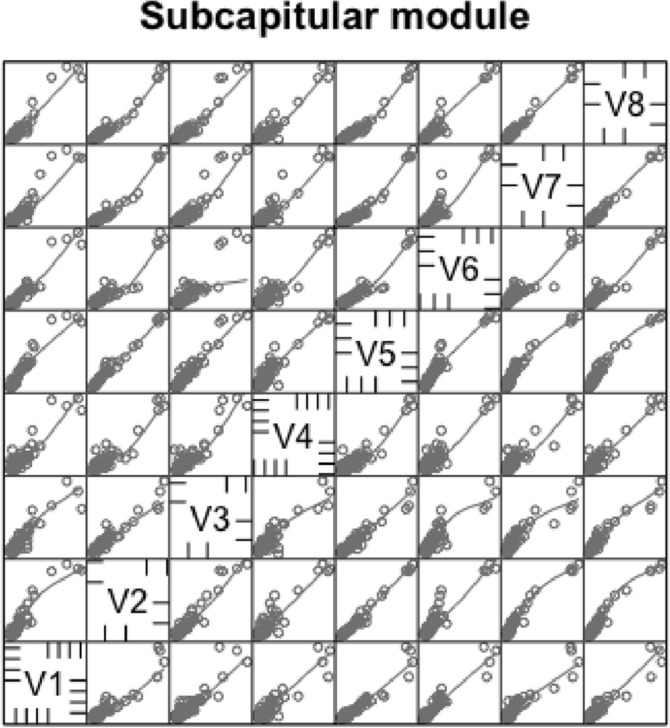
Scatterplots (plus LOESS lines) for subcapitular measures [1] to [8] taken (see Fig. 1), labeled as V1 to V8 over the species reviewed. *V3* deutosternal groove length *GL*,  *V4* deutosternal average width (without lateral extensions) *AGW*. Note lack of linearity between *GL* and *AGW*

The eight subcapitular measures for each species can be found in Table 2. All hypostome measures were in general highly positively correlated with each other and with the idiosomal index (*IL*). There some outliers (sometimes including *Uropoda abantica*) or a degree of non-linearity present in most $2\ by\ 2$ plots (Fig. 9). The proportion of variance in one measure attributable to the variance in another ($R^{2}$) ranged from a low 0.888 (deutosternal groove length versus hypostomal width at the base of the corniculi) to a high 0.991 (*basis gnathosomatica* length versus overall subcapitulum length) when the subcapitulum was considered as a distinct module. This indicates that widths of structures may be adjusted somewhat independently of lengths i.e., width–length aspect ratios may be important in discerning different mite adaptations like that already found in mite chelicerae (Bowman 2020, 2021). Further, that the investment in achieving hypostomal length is generally in sync with that for the *basis gnathosomatica* (i.e., concerted elongation) may be occurring during evolution.

With the exception of *U. abantica* at 0.876, the width–length aspect ratio *AGW*/*GL* varies within each family by a generally similar extent (Table 2). *Amblyseius omaloensis* (Phytoseiidae) shows the minimum (0.062) overall and *Macrocheles forceps* has a notable value at 0.408.

With respect to overall body size, the maximum correlation of subcapitular measures with idiosomal index (*IL*) was unsurprisingly with the overall subcapitular length ($r=0.980$), indicating a general gnathosomal scale factor at play. The minimum correlation to *IL* values was with hypostomal width at the bases of the corniculi ($r=0.952$) suggesting slightly different processes may be important perhaps in hypostomal breadth at the cornicular bases than just scale (see below).

Amongst the lengths, the minimum correlation was $r=0.946$ (deutosternal groove length versus length of corniculi), the maximum correlation was $r=0.995$ (*basis gnathosomatica* length versus overall subcapitulum length). So, cornicular length may be determined by factors somewhat different than that determining deutosternal groove length (see below).

Amongst the widths, the maximum correlation was $r=0.989$ (*basis gnathosomatica* width versus hypostomal width at the base of the corniculi), the minimum correlation was $r=0.959$ (hypostomal width at the base of the corniculi versus average deutosternal groove width). So, although the hypostome and *basis gnathosomatica* show concerted widths, the average deutosternal groove width appears somewhat dissociated indicating some other process may be going on in determining it (that is $\tfrac{AGW}{Width\ at\ bases\ of\ corniculi}$ ranges over an order of magnitude, min$=0.0980$, max$=0.8862$). Note that a quadratic relationship between *AGW* and *IL* produced a better empirical fit to the data points than just a linear relationship (although the $R^{2}$ was slightly lower, 0.9039 versus 0.9298). The corollary of this latter point is discussed below.

Further evidence that processes involved in subcapitular structure lengths may be somewhat distinct to those involved in determining widths is that the geometric mean of $R^{2}$ values over all combinations of lengths (excluding *IL*) measured with widths is lower (at 0.927) than either the geometric mean of $R^{2}$ over all the lengths as a set (where $R^{2}=0.944$), or that over the set of all the widths measured (where $R^{2}=0.951$).

Indeed linear regression coefficients (of 0.9–1.1, i.e., within 10% of unity) through zero within the subcapitular module were seen between

- subcapitular length and *basis gnathosomatica* width,
- hypostomal length and the hypostomal width at the bases of the corniculi,
- *basis gnathosomatica* length and deutosternal groove length (where $R^{2}=0.9684$).

From the first two results, this indicates a square basis for the overall design of the ventral part of the gnathosoma and generally independent of this the hypostome itself. Further, from the last result, that what determines deutosternal groove length might be also that which determines the *basis gnathosomatica* length, and counterfactually, the length of the groove shows a degree of independence with any hypostomal size changes. The corollary of the first conclusion is that the *basis gnathosomatica* itself must be rectangular (see below).

Liu et al. (2017) gives evidence that hypostomal measures may define their groupings of specialist versus generalist versus pollen feeding phytoseiids. Indeed looking at the size adjusted $\tfrac{hypostomal\ length\ [1]}{IL}$ and $\tfrac{corniculi\ length\ [6]}{IL}$ values in the species reviewed herein shows that most phytoseiids as micro-cephalics (Bowman 2020) have noticeably lower values for both measures than most mega-cephalic macrochelids. The phytoseiid subcapitular design is one of deadly daintiness.

### Proportionality of cheliceral measures

**Fig. 10:**
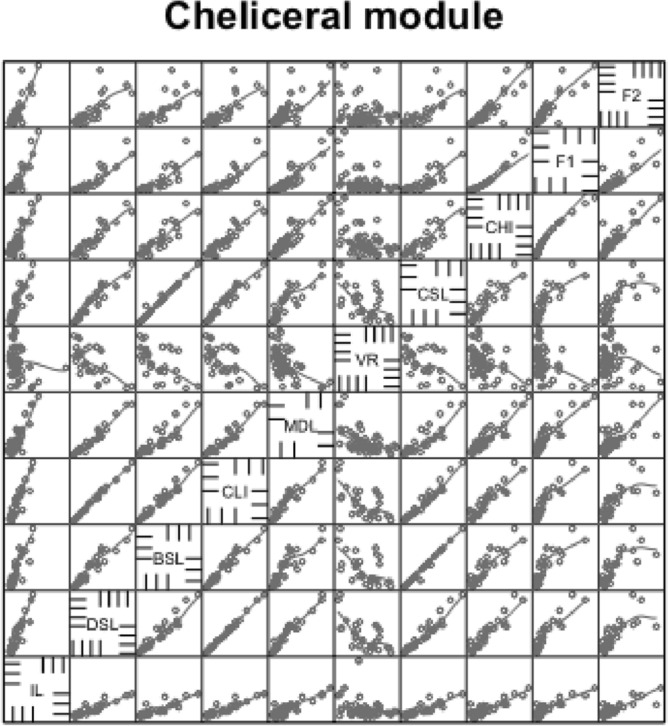
Scatterplots (plus LOESS lines) for cheliceral measures taken (see Bowman 2020)

The cheliceral measures for each species can be found in Table 3. Cheliceral measurements were in general positively correlated with each other and with the idiosomal index (*IL*) (Fig. 10). Unsurprisingly due to its formulation, the maximum correlation amongst cheliceral measures (as a separate module) was reach (*CLI*) with cheliceral distal segment length ($DSL,\ r=0.999$). The minimum correlation was between moveable digit length (*MDL*) and cheliceral shaft length ($CSL,\ r=0.928$) indicating some other processes may impact moveable digit length rather than just gnathosomal elongation (see Discussion on reach and velocity ratio in Bowman 2020). When just lengths were considered, the situation remained the same. With respect to overall body size, the maximum correlation of cheliceral measures with idiosomal index (*IL*) was $r=0.980$ with reach (*CLI*), suggesting that for this set of mites studied herein there were few if any excessive reach species like veigaids. Given the small size of phytoseiids, a restricted range of reach (*CLI*) values is expected. The minimum correlation with *IL* was with *MDL* at $r=0.949$ again pointing to a degree of independence in moveable digit length with overall scale.

Indeed linear regression coefficients (of 0.9–1.1, i.e., within 10% of unity) through zero within the cheliceral module were seen between *DSL* and *CSL*, unsurprisingly due to their derivation and between *MDL* and gnathosomal width. The latter may simply relate to the predicted arthropod feeding species usually having large *F*2 (and thus large *F*1 chelal adductive forces) in the species used in this study (see Fig. 7).

### Interdependence of hypostomal and cheliceral structure sizes

Liu et al. (2017) is the only mesostigmatid study quantitatively co-analysing cheliceral and hypostomal morphology as an ensemble. Although those authors combine lengths, widths, angles and meristic count characters together, their first principal component (PC1) has strong elements of overall size in its loadings and is dominated by cheliceral measures. The size of a mite must determine its feeding performance in some way. The six hypostomal measures they used: corniculi length, internal malae length, tritosternum length, subcapitular groove length ($\approx GL$?), number of denticles scales in the subcapitular groove, and number of denticles of every scale in the subcapitular groove, have conflicting loadings on PC1 suggesting partial independence in their evolutionary determination versus the chelicerae. In the current review of species herein, whether looked at overall, or just the set of length measures, or just the set of width measures, both the maximum and the minimum proportion of variance in one measure attributable to the variance in another ($R^{2}$) was always lower when considering association between subcapitular versus cheliceral measures than those within each morphological module (range of $R^{2}=0.818{-}0.991$). This suggests again some possible evolutionary independence between the design of each set of structures. In other words, the design of the cheliceral module and that of the subcapitular mesostigmatid module are partly decoupled.

**Fig. 11:**
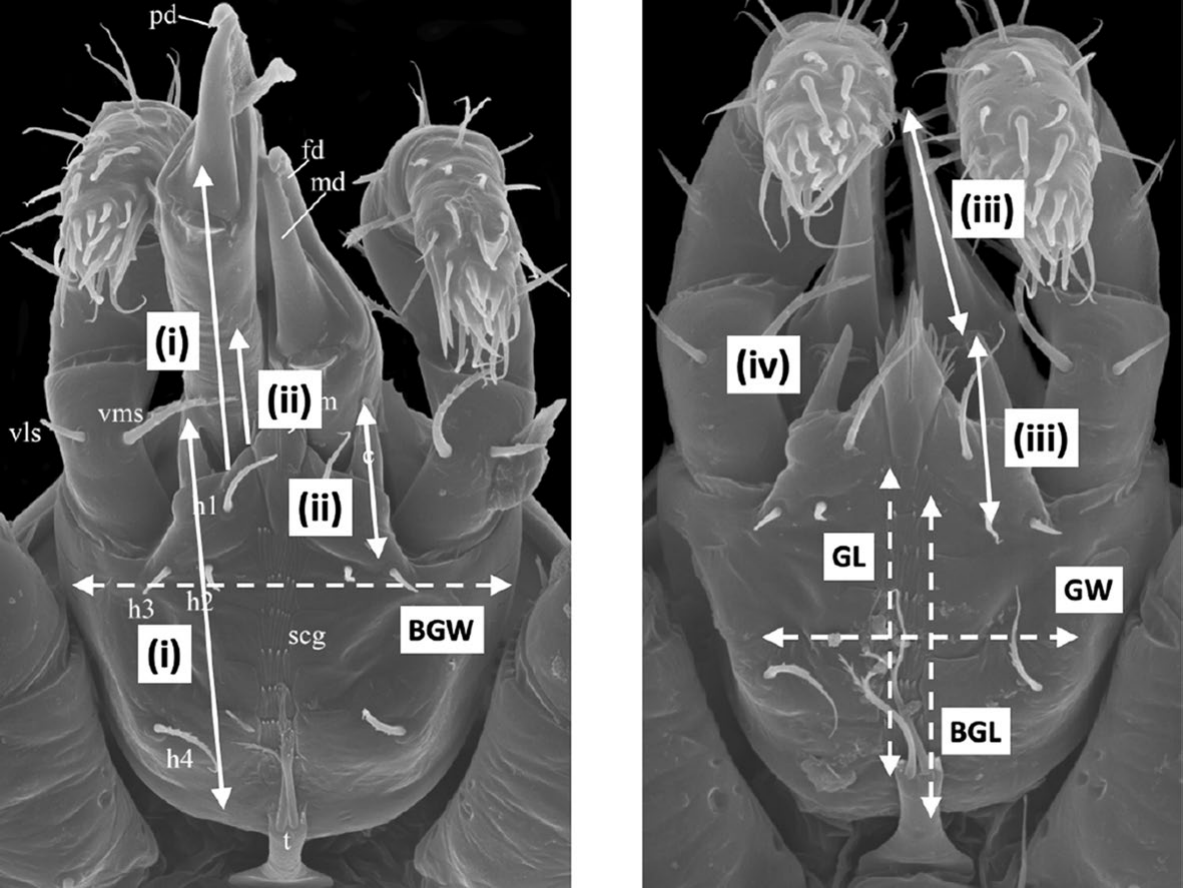
Illustrative consilience of gnathosomal structure sizes. SEM of venter of *Zercon albanicus* amended from Ujvári (2011) $\copyright$ with permission. Left: female. Solid lines and highlighted boxes for each point in text. Dashed line is *basis gnathosomatica* width. Right: male. Solid lines and highlighted boxes for each point in text. Note supportive function of corniculus (iv). Dashed lines are deuterosternal groove length (*GL*), *basis gnathosomatica* length (*BGL*), and gnathosomal width (*GW*) sensu (Bowman 2020)

Examining only the linear regression coefficients (of 0.9–1.1, i.e., within 10% of unity) through zero between the subcapitular module and the cheliceral module numerically confirms some basic assumptions that acarologists already have across mesostigmatids:

(i) The cheliceral shafts could just fit nicely sitting above the subcapitulum interior to the mite when the chelicerae are retracted, leaving the chela exposed anteriorly (see Fig. 6), as the length of the cheliceral shaft (*CSL*) (and particularly that of the distal segment *DSL*) are numerically consilient with the subcapitulum length (Fig. 11 Left).
(ii) The cheliceral basal segment could snugly sit above the hypostome on full cheliceral protrusion, as the distal cheliceral segments (*DSL*) are pushed between the palps (see Fig. 5) since the length of the basal segment of the chelicera (*BSL*) matches the length of the hypostome (Fig. 11 Left).
(iii) The moveable digit could then sit above the hypostome on full cheliceral retraction (see Fig. 6), as the moveable digit length (*MDL*) of the chela closely matches the hypostomal length (Fig. 11 Right). This would agree with the function of the chela in holding food material being dragged back towards the labrum and masticated there by the chelal digits. This could be independently validated by acarologists examining veigaids in particular who represent extreme chelal digit elongation (Bowman 2020).
(iv) The corniculi are positioned to support the cheliceral shafts as they move in and out (Fig. 11 Right), as cheliceral height (*CHI*) broadly matches the hypostomal width at the bases of the corniculi. Recall that the centres of two sub-cylindrical shafts of diameter approximately *CHI* sitting next to each other would be the same *CHI* apart. Consiliently the highest variation explained of cornicular lengths with cheliceral measures is for *BSL* ($R^{2}=0.9536$). This segment would need the largest support to resist flexure/bending upon cheliceral protrusion. This review does not support the view that corniculi are jointed at their base (as in Wernz 1972). Cheliceral height is an indicator of chelal adductive force (*F*1) and thus crunch force *F*2 (i.e., indicating powerful chelal closing muscle-filled chelicerae) so a degree of future validation could be by looking for particularly long corniculi in such designed predators.

For *Antennocheles* (i) applies only because the elongated cheliceral shafts seem to be telescoped by a sheathing mechanism within the body (Evert Lindquist *pers. comm.*). For (ii) this would have to apply to the second-most anterior cheliceral shaft segment in *Antennocheles* as its cheliceral ‘basal segment’ can be ensheathed well into the podosoma, well posterior to the hypostome. Elongated middle segments are known (Woodring and Galbraith 1976), as are divided basal segments (Athias-Binche 1982). However, (iii) seems to hold as well for *Antennocheles* (Evert Lindquist *pers. comm.*). Arising from (iv) is a conjecture, for future investigation by acarologists, that the length of the membraneous internal malae may therefore be more related to *CHI* (i.e., they are part of the cheliceral design module, rather than the subcapitular design module) as they almost certainly project between the moving cheliceral shafts to clean them of debris. Note that Liu et al. (2017) finds the internal malae relatively long for the specialist pollen feeding phytoseiid *Euseius utilis* (and such excrescences are considerably developed in trigynapsids, Owen Seeman *pers. comm.*).

Furthermore, three other numerically close relationships are present:

- Gnathosomal width broadly matches both the *basis gnathosomatica* length ($R^{2}=0.9540$) and the deutosternal groove length ($R^{2}=0.9684$) (Fig. 11 Right), and
- The *basis gnathosomatica* width broadly matches cheliceral shaft length (*CSL*) $R^{2}=0.9684$ (Fig. 11 Left).

**Fig. 12:**
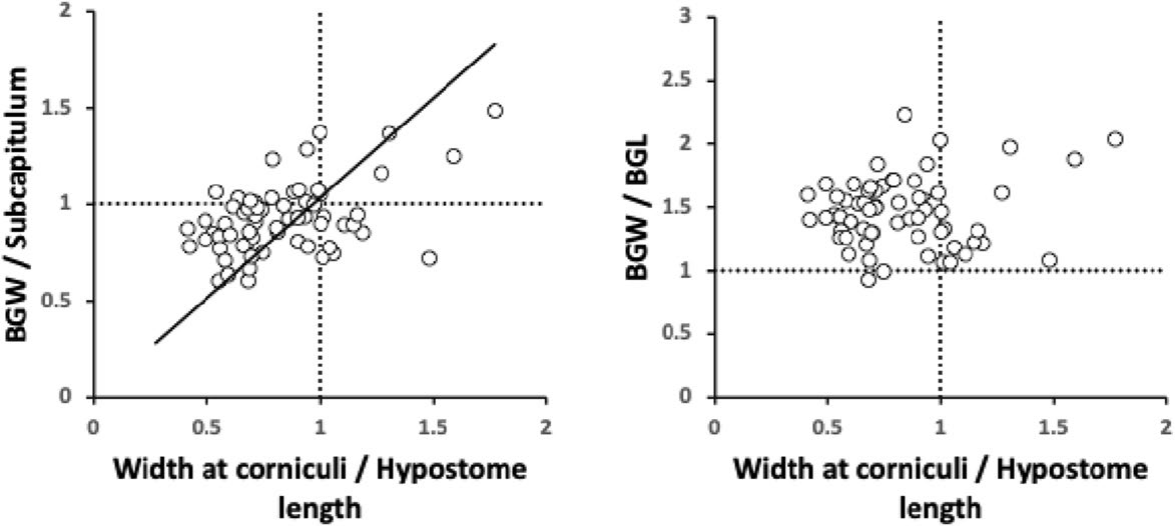
Aspect ratios within subcapitulum, all species studied including *Uropoda abantica*. *Left* self-similarity structures at different scales. Solid regression line through zero $slope= 1.0342,\ R^{2}=0.9255$ almost through equality point, showing width at cornicular bases only slightly underestimates triangular hypostomal width. Note cluster ($<1$) above regression line in left lower quadrant shows that most species have an even more elongate hypostome than any elongation of the subcapitulum itself. *Right* the *basis gnathosomatica* is fundamentally rectangular (broader than wide)

It is not clear why this extra functionally should be so. For sure, in *Antennocheles*, the *basis gnathosoma* width is far less than cheliceral shaft length (Evert Lindquist *pers. comm.*). Some species of *Zerconopsis* may also provide other exceptions (i.e., those with three-segmented cheliceral shafts/basal segment sub-divided, Evert Lindquist *pers. comm.*). Note that the subcapitulum is broadly square (overall average $\tfrac{BGW}{subcapitulum}=0.9084$) but the hypostome is a different rectangular shape (overall average $\tfrac{width\ at\ cornicular\ bases}{hypostome}=0.8109$). These are only mildly correlated ($r=0.3828$)—see results above showing their widths are driven differently, and Fig. 12. Gnathosomal width (estimated from *CHI* values) correlates well with $BGW, r=0.9778$. Then, if the *basis gnathosomatica* length is related to pharyngeal musculature, and the deutosternal length somehow related to volume of fluid transported, then the first bullet point above infers mites with large chela adductive force *F*1 (‘gnathosomatistion’ sensu Bowman 2020) might also have a need for strong imbibition processes.

If cheliceral shaft length is instrumental in the volume of fluid temporarily stored in the circumcapitular groove, then this would drive *basis gnathosomatica* widths given the sub-circular design of mesostigmatid gnathosomas as in the second bullet point above. On cheliceral retraction a volume approximately scaling with its total shaft length (recall that the chelal digits remain external) would be displaced internally, causing a rise in intra-idiosomal pressure and perhaps increased passive filtration of haemocoelic fluids by and through the coxal glands—thus contributing to circumcapitular groove fluid volumes. However why these sizes should be around equality (slope $\approx 1.0$) is not clear, it just could be a co-incidence. One possibility is that long cheliceral digits occupy large internal volumes in the subcapitulum, pushing other propodosomal tissues laterally and thus requiring a wider *basis gnathosomatica* volume to accommodate them. The independence of deutosternal/tritosternal structures with cheliceral reach is confirmed for instance in *Antennocheles* where the former are not particularly modified but cheliceral segment length comprises almost the whole podosoma (Evert Lindquist *pers. comm.*).

Given the above it is unsurprising that the width–length aspect ratio $\tfrac{AGW}{GL}$ (ignoring *U. abantica*) positively correlates well with: the cheliceral aspect ratio $\tfrac{CHI}{CLI}$ ($r=0.9472$), $\tfrac{BGW}{subcapitulum\ length}$ ($r=0.9497$) and $\tfrac{width\ at\ cornicular\ bases}{hypostome\ length}$ ($r=0.9101$). Mites vary consistently in the shape of their trophic structures.

However, the moveable digit velocity ratio (*VR*) is unrelated to most gnathosomal measures. The chelal design appears to be independent of: cheliceral elongation ($\tfrac{CHI}{CLI}$, $R^{2}=0.0011$), subcapitular design ($\tfrac{BGW}{subcapitulum\ length}$, $R^{2}=0.0139$), hypostomal elongation ($\tfrac{width\ at\ cornicular\ bases}{hypostome\ length}$, $R^{2}=0.0929$), *basis gnathosomatica* shape ($\tfrac{BGW}{BGL}$, $R^{2}=0.0581$) and, deutosternal groove aspect ratio $\tfrac{AGW}{GL}, R^{2}=0.0057$).

### Hypostomal volume bite size

The area of the triangular hypostome can be approximated by

$$\tfrac{1}{2}.(width\ at\ base\ of\ corniculi).(hypostome\ length).$$

Above this is potentially a *CHI*-worth height of food material, but this would only be true if both chelicerae with their chelal digits were fully retracted. As the chelicerae usually work alternatively to drag material into the labrum area then the actual food volume above the mesostigmatid hypostome at any one time is therefore

$$\tfrac{1}{4}.(width\ at\ base\ of\ corniculi).(hypostome\ length).CHI.$$

As expected from the size adjusted hypostomal lengths discussed above, phytoseiids have the lowest values on average of all the families studied herein for hypostomal volume bite size ($6570\ \upmu$m$^{3}$). The range of values over the individual phytoseiid species ($4864{-}10,705\ \upmu$m$^{3}$) are around the maximum food fragment size grabbed (*MvG*) values listed by Bowman (2023) for free-living astigmatids ($4703{-}7786\ \upmu$m$^{3}$) calculated from their chelal sizes, confirming the trophic daintiness of traditional BCAs. Rhodacarids on average show only about twice that hypostomal volume bite size of phytoseiids (i.e., $10,144\ \upmu$m$^{3}$, and in at least one species a similar reasonably high chelal velocity ratio). Other relatively low value species are the blattisocid *Lasioseius orangrimbae* ($8502\ \upmu$m$^{3}$), the digamasellid *Dendroseius reductus* ($7792\ \upmu$m$^{3}$) and the groups of ascids ($18,800\ \upmu$m$^{3}$), eviphidids ($12,744\ \upmu$m$^{3}$) and melicharids ($7967\ \upmu$m$^{3}$). On average macrochelids (at the much larger $98,503\ \upmu$m$^{3}$) have a hypostomal volume bite size of 15 times that of phytoseiids, and parasitids (at the even larger $111,769\ \upmu$m$^{3}$) have on average 17 times that of phytoseiids. The families of yet bigger mites even more (up to 139 times).

Bite size is surely related to wound size inflicted on the prey. Phytoseiids are thus comparative nibblers. Indeed, larger biters may not be good candidate BCAs in that they may effectively completely destroy tetranychids on initial attack causing them to release alarm pheromones and disperse. Phytoseiids in only slightly cutting into them, dissolving them slowly and drinking them in semi-alive may be a more subtle tactic and could avoid this release (a simple experiment might prove this?). At a $\hat{V_{p}}\approx 6.68\ \upmu {\text {m}}^{3}/10^{6}$ for a typical tetranychid (Table 6) the wound from an average macrochelid or parasitid single chelicera cutting/crunching into the prey would be equivalent to around 1.5–1.7% of total prey body volume. As a single bite, this is equivalent to a shark biting off: three human hands, or a little more than a whole human foot, or a little less than a whole human forearm (https://robslink.com/SAS/democd79/body_part_weights.htm). Considering an optimistic survivable loss of 45% of one’s body on injury (https://www.sciencefocus.com/the-human-body/how-many-organs-in-the-body-could-you-live-without/#), means that around as few as 15 attacks by each macrochelid or parasitid chelicera would effectively kill a tetranychid (and therefore completely mangle it in seconds). Compare this to the stealthily biting phytoseiid at a wound size around 0.1% of total prey volume (equivalent to about one human finger-worth for a shark attack). Hardly an alarming great loss. Injection of a neuro-arrestant and extra-corporeal digestive enzymes would allow a leisurely consumption of the whole tetranychid volume by the phytoseiid without alarming other prey mites.

### Deutosternal footprint and ‘tubification’

The length of the deutosternal groove was always shorter than the length of the subcapitulum (being equivalent to about 59% of its length on average) and usually slightly shorter than (i.e., equivalent to on average 94% of) the length of the mites’ *basis gnathosomatica*. Meaning in theory it could be positioned solely to link the circumcapitular groove reservoir with the hypostome.

Five out of six phytoseiids had groove lengths matching the length of their *basis gnathosomatica*. However, in 24 (of the 72) species examined it exceeds the length of the *basis gnathosomatica* by at least 10% indicating that even if it spanned all of the latter, it must be at least partly *integrated* into the hypostome as well in these mites. This is confirmed in Wernz (1972) where the position of the most anterior row of deutosternal teeth is slightly anterior of seta $h_{3}$. Indeed, on average macrochelids have a deutosternal groove length 19% bigger than their whole *basis gnathosomatica* herein, suggesting it must at least be implicated in hypostomal fluid control even if positioned posteriorly tight on the circumcapitular groove. Again this is confirmed in Wernz (1972) where the position of the most anterior row of deutosternal teeth in macrochelids is slightly anterior of seta $h_{3}$ despite different species being included than in this review. Whether all mesostigmatids have tritosternal lacinae inserted into the meniscus of prey fluids forming a ‘ring’ (as far posterior as hypostomal seta $h_{1}$) enabling tip contact with retracted cheliceral arthrodial brushes as observed in *Parasitus* sp. (Wernz 1972) remains to be seen.

The exact relative positioning of structures is unclear. Wernz and Krantz (1976) suggests that the entire tritosternal base is posterior to the subcapitulum, with only the lacinae being aligned to the deutosternum. Fred Beaulieu (*pers. comm.*), recounts that the tritosternum base probably starts more posteriorly than the most posterior (basal) position of the deutosternum (or subcapitulum), in a resting, live position in mesostigmatids which may explain the tritosternum as a whole being so long. Is the tritosternal base ever elongated to reach over the subcapitulum? How do species with fused lacinae (which then look like having extended bases) fit in a unified schema? The deutosternal groove is therefore regarded herein as a part of the whole subcapitular module rather than a separate entity or solely related to the *basis gnathosomatica*. Follow-up work should look for any functional relation between the sizes of gaps at the ends of the deutosternal groove and the extrema of the subcapitulum. Is there an advantage of the deutosternum being relatively advanced or regressed within the subcapitulum? The part-integration design comprised 9 out of the 11 macrochelids, 5 out of 10 laelapids and 3 out of 8 parasitids, suggesting either a phylogenetically driven distinction or something particular about how these mites feed compared to the other mesostigmatid families. Could it be that the deutosternum is not directly related to chelal function at all but determines *only* a fluid handling competency and that these families consume particularly high volume prey for their body size? Do such mites have matching specialised tritosterna? Do they have particular designed internal malae or other differentiations in the anterior of their hypostome?

There is a widely held view amongst acarologists that apomorphy in parasitiform mites is an evolutionary trend towards more efficient liquid feeding via an increasing tendency for tubular mouthparts. Particulate feeding mesostigmatids being unusual. What about in this study? The overall average values of $\tfrac{CLI}{CHI} \equiv \tfrac{1}{cheliceral\ aspect\ ratio}=0.2358$, the overall average $\tfrac{AGW}{GL}=0.1561$. Examining the mites herein (omitting the derivative *U. abantica*), found no evidence that mite cheliceral designs tending towards long narrow elongate tubes (as measured by high values of $\tfrac{CLI}{CHI} \equiv \tfrac{1}{cheliceral\ aspect\ ratio}$) are correlated with deutosternal grooves being similarly tubular (as measured by low values of $\tfrac{AGW}{GL}$) across the mites studied herein, $R^{2}=0.0582$. Within the subcapitular module, the relative elongate nature of the deutosternal groove was similarly not well correlated with either the overall subcapitular relative elongation ($\tfrac{BGW}{subcapitulum},R^{2}=0.1092$) nor with the hypostomal relative elongation (estimated by $\tfrac{hypostomal\ length}{width\ at\ base\ of\ corniculi}$, $R^{2}=0.0404$) either. Whilst the cheliceral design and the rectangular hypostomal/subcapitular design are linked by mechanical constraints (see above), the deutosternal groove itself is partly decoupled from them. Rather its design may have more to do with the volume (and thus the type) of prey consumed. Indeed apomorphy in ameroseiids is characterised by having a single, wide, multi-denticulate, transverse ridge extending lateral of the deutosternum at the level of ca. the 6th row of deutosternal ridges (Evert Lindquist *pers. comm.*), for example Fig. 5 of *Ameroseius lidiae* in Khalili-Moghadam and Saboori (2014) and not even a tubular design.

Regarding ancillary subcapitular transverse ridges, etc., several families have notable extra features beyond the lateral margins of the deutosternal groove (species marked * in Table 1). These can effectively increase the width of parts of the deutosternal groove by three or more times in some arctacarids, some of the phytoseiids and the single heterozerconid studied (Table 4). Facultatively these taxa may be designed to be able to handle excesses of prey fluid overspill if they anecdotally occur. On average an increase of 59% in groove width was observed. That is, whilst these mites are designed for a certain trophic ‘business-as-usual’, they have the possibility of tackling unusually watery or high-volume prey opportunistically from time to time. Does this match feeding records? There is much more work for acarologists to do. Could these extensions be the subcapitular plesiomorphic state? That is, strict deutosternal canalisation being the derived condition? This would infer that phytoseiid BCAs are the least plesiomorphic. An improved detailed phylogeny within mesostigmatids based upon genetic markers would help.

### Comparative deutosternal groove flow rates

**Fig. 13:**
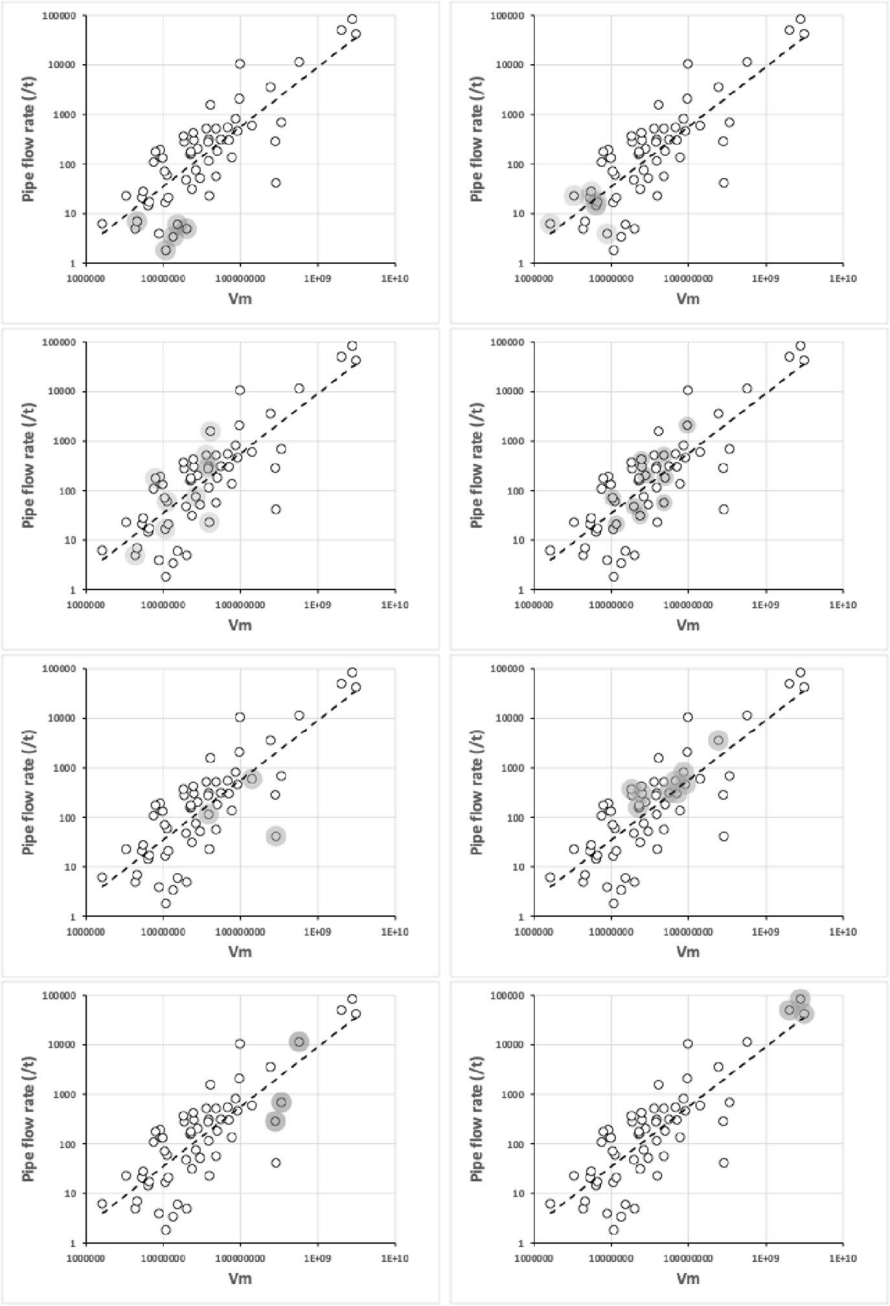
Deutosternal pipe flow rate *PFRt* (estimated by $\frac{AGW^{4}}{GL}$) versus predator volume (in $\upmu$m$^{3}$) for all the species studied herein including *Uropoda abantica*. Each featured family highlighted with grey halos. Sub-figures ordered left to right in approximate average mite sizes for that family. *Top row* the small sized phytoseiids, rhodacarids. *Second row* the medium sized ascids, laelapids. *Third row* the large sized parasitids, macrochelids. *Bottom row* the very large sized arctacarids, megisthanids. The remaining species including *Uropoda abantica* are scattered centrally

Figure 13 shows that the estimated deutosternal groove flow rates (*PFRt*) generally increase with body volume ($V_{m}$, $R^{2}=0.8633$). Families look distinct both in body volume and deutosternal flow rate. Larger mites have larger flow rates commensurate with possibly dealing with larger prey comprised of larger amounts of fluids. Phytoseiids are a notable low flow outlier group even compared to similar size mites like the rhodacarids. If *PFRt* is normalised by mite volume $V_{m}$ phytoseiid values are very low indeed. Pumped deutosternal pipe flow may not be a key parameter in their fluid handling, rather typical BCAs may rely upon optimised passive microchannel flow dynamics (i.e., filament-propagation based flow). Their natural prey may thus be quite dry.

Deutosternal tubification as measured by $$\tfrac{AGW}{GL}$$ is not well predicted by circumcapitular volume (A, $$R^{2}=0.0435$$), deutosternal volume (DV, $$R^{2}=0.0533$$), or the volume available from the protrusion/retraction of a single chelicera (B, $$R^{2}=0.0482)$$. It was only mildly correlated with *PFRt* ($$r=0.5034$$). Instead, the range of *PFRt* values could be better considered as defining functional groups sensu (Walter and Proctor 2013), based upon the need to shift fluid volumes commensurate with those mites’ typical prey in a feeding bout. The latter being impacted upon by the predator’s body size. Overlain on this relationship is that for this review as expected from Bowman (2020), larger species (which tended to be megacephalics) usually had lower chelal velocity ratios, higher chelal crunch force *F*2 values and larger reach (*CLI*). In phytoseiids there may not be the need for very high initial feeding rates if they steadily consume modest size relatively dry prey caught nearby in small chunks and steadily drink their prey’s liquidised tissues through small incisions (Flechtmann and McMurtry 1992a). In a sense they appear to be ‘browsers’ not true predators. One could consider them almost as kleptoparasites of the herbivores accessing sugars etc. secondarily, that the plant pest is extracting from the plant.

### Comparison of gnathosomal compartments

Microfluidic technology is defined as the precise manipulation of very tiny quantities of fluids (from $10^{-9}$ to $10^{-18}$ L) by leveraging delicate channels with the dimension of tens to hundreds of micrometres (Whitesides 2006). Table 5 shows the circumcapitular groove volume (A), the volume displaced by a chelicera (B) and the deutosternal volume (DV) values for the species in this study. The mite volumes (as water) are in the correct ball-park for microfluidics to apply as $1\ \upmu {\text {m}}^{3}=(10^{-6})^{3}\ {\text {m}}^{3}=10^{-18}\ {\text {m}}^{3}\equiv 10^{-15}\ {\text {L}}$ [since $1\ {\text {L}}=1000\ {\text {cm}}^{3}=10^{3}.(10^{-2})^{3}\ {\text {m}}^{3}=10^{-3}\ {\text {m}}^{3}$], given the range of maximum volume (i.e., *Proarctacarus johnstoni*) $=5.09 \times 10^{-9}\ {\text {L}}$ available for prey fluids on single cheliceral retraction, and the minimum volume (i.e., the deutosternal volume in *Amblyseius omaloensis* $=3.69 \times 10^{-13}\ {\text {L}}$).

Given that in Fig. 4 of Bowman (1984) the first deutosternal denticulated ridge of a typical soil pergamasid is $\approx 10\ \upmu$m away from the edge of the ventral sternal plate and the deutosternal groove width is $\approx 36\ \upmu$m, the circumcapitular groove volumes (A) may be underestimates if there is a degree of cuticular folding/unfolding at the gnathosoma to idiosoma junction say on gnathosomal flexure or retraction/protrusion. Such adjustments during feeding are described by Bowman (2014). Similarly, implicitly by using the cheliceral shaft volumes in the calculation for the displaced pool of fluid when the chelicerae move about (‘Tubevolume’), one is assuming that the gnathotectum is the upper boundary of the cylindrical fluid ‘ring’ (Wernz 1972). This simple assumption that its anterior margin aligns with the tip of the hypostome may be slightly wrong but simplifies calculations. Its often fimbriate epistome border (like the corresponding hypostomal internal malae) in many mesostigmatids must be related somehow to holding the fluid meniscus, much like those setae near the circumcapitular groove in say *Epicroseius* sp. for example. A follow-up study of gnathotectal lengths, divisions, fimbriations etc., would be useful particularly in micro-cephalic eviphidids where the epistome is very elongate centrally.

**Fig. 14:**
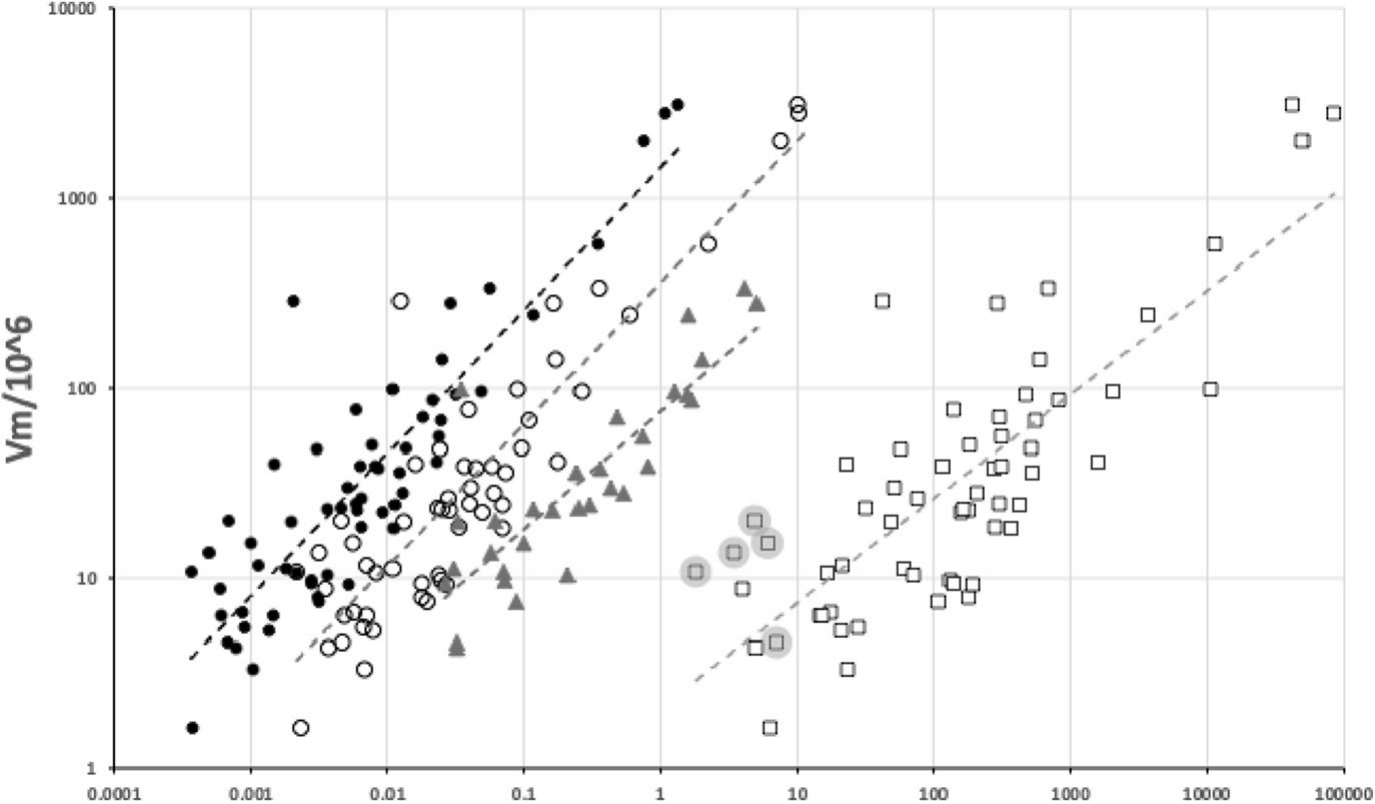
Gnathosomal compartment volumes with regression lines showing general scaling with body volume (includes *Uropoda abantica*). *Open dots* circumcapitular groove volume [estimated by $A=\pi .BGW.(AGW^{2})/10^{6}$]. *Black dots* deutosternal volume [estimated by $\frac{\pi }{4}.AGW^{2}.GL/(10^{6})$]. *Grey solid triangles* the volume available for prey fluids on a single cheliceral retraction [estimated by $B=\pi .Reach.\frac{0.828}{4}.CHI^{2}/(10^{6})$]. *Open squares* deutosternal pipe flow rate *PFRt* (estimated by $\frac{AGW^{4}}{GL}$) with phytoseiids highlighted by grey halos

The volume of each of the three gnathosomal compartments [deutosternal volume, circumcapitular groove volume (A), and the volume displaced by a single chelicera (B)] all linearly scale well with idiosomal volume $V_{m}$ ($R^{2}=0.9837$, 0.9863, 0.8794, respectively)—Fig. 14, and thus also scale well with *IL*. The deutosternal pipe flow rate (*PFRt*) similarly scales ($R^{2}=0.8792$) with idiosomal volume, but is noticeably more variable over the mites species. This variability is marked when plotted against the volume available from a single cheliceral protrusion/retraction (*Figure not shown*) since the latter measure is the most likely to be affected by cheliceral adaptations, e.g., extended reach (*CLI* increase) or chelal adaptations, e.g., high crunch force *F*2 (driven by increased *CHI*). In each case the phytoseiid species show low compartment volumes at the bottom of the point clouds (*PFRt* highlighted in Fig. 14). Their small volume available from a single cheliceral retraction is consilient with: the small jaw ‘stabbing’ or a crushing/mashing kill style and the ‘lapping up fluid’ mode of feeding characterised in Bowman (2020) and, that the chelicerae are protracted beyond the corniculi by the length of the moveable digit (Flechtmann and McMurtry 1992a). Phytoseiid fixed digits remain outside of attacked legs (Flechtmann and McMurtry 1992a) and the latter can be used like “...a straw to extract fluids from the whole prey mite...” through modest ($5{-}50\ \upmu$m) cuts in the cuticle. Cheliceral lobes in phytoseiids may form an extra ancillary tube to retain these fluids (Flechtmann and McMurtry 1992a) and prevent prey tissue overspill during such ‘nibbling’. Phytoseiids predate in a particular manner.

Bigger mites clearly can handle bigger volumes, however, the regression lines gently flatten as one moves left to right from measure to measure in Fig. 14. So if one takes the deutosternal volume (or deutosternal ‘gulp’) as the basis, bigger mites have disproportionately bigger circumcapitular groove volumes (i.e., they can store more coxal recycling volumes per deutosternal gulp, or alternatively they can gulp a lesser proportion of their temporary depot each time) than small mites. The bigger mites appear to need to be major shifters of prey overspill/coxal fluids or rely upon the deutosternal volume itself as an overspill buffer store (as Wernz and Krantz 1976 effectively assume). Bigger mites also may have slightly more volume available for prey fluids on a single cheliceral retraction/protrusion than expected per deutosternal gulp, but for sure large mites have disproportionately increased deutosternal pipe flow rates per gulp. So large mites may take relatively shorter time to move their bigger reservoirs from place to place. Indeed, considering (the admittedly only indicative) ratio $\frac{PFRt}{deutosternal\ volume}$ shows that phytoseiids exhibit noticeably low ratio values compared to where mites of their size would be predicted given the other families. Phytoseiids thus may be small and slow fluid shifters (i.e., they are designed as fluid ‘tricklers’). Taking the mesocephalic taxa as the basis, micro-cephalic species have approximately 2.5 times smaller volume available for prey fluids on a single cheliceral protrusion/retraction. However, megacephalic species have an approximately 3.5 times more, suggesting that these substantial large gnathosoma-bearing mites may be adapted to deal with very large fluid volumes comparatively. Could the small deutosternal groove in phytoseiids ensure a slow continuously flowing narrow thread of fluid is occurring? Note in particular that increasing the deutosternal groove width by 50% in phytoseiids of the same *IL*, means for the same prey and groove length, a resultant $1.5^{4}=5$ times fluid flow suggesting that they are flow limited when at small size.

The volume available for prey fluids on a single cheliceral retraction/protrusion (B) is equivalent to a minimum of 0.4 times (in *U. abantica*), and a maximum of 21.2 times (in arctacarids) that which can be temporarily stored in their respective circumcapitular grooves (A), if the latter were regarded as buffering reservoirs. At overall on average of 9.7 times, these are large multiples and accordingly suggest that either prey overspill occurs at only fractional cheliceral protrusion/retractions, or only anecdotally on small scale whole gnathosomal adjustments, or that this mode of action whereby fluids majorly flow posteriorly through the deutosternum assembly as traditionally thought by acarologists for most mites is actually unlikely. Irrespective of deutosternal volumes, there is nowhere else than the circumcapitular groove for a major bolus of fluids to go once they arrive from this postulated backwards flow. There would be rather quick saturation of the putative buffer store (A) in practically all the mites reviewed. Moreover as circumcapitular volume is at most 11 times that of their corresponding deutosternal volumes (e.g., in *Arctoseius wisniewski*) and usually are approximately half of this, instead, the scale of these deutosternal gulps would be more effective at repeatedly emptying a full circumcapitular groove of accumulating recycled fluids from coxal secretory processes promptly forward within only about six goes on average (and then re-imbibition of the fluid into the gut). This would be a useful ability to handle high and repeated recycled coxal secretion volumes if necessary during their feeding. This potential rapid draining matches the relatively prompt emptying of the circumcapitular suture once a predatory mite discards its prey (Bowman 2014), and the very slow disappearance in tritosternumectomised mites observed in Wernz (1972). Recall that, at least in pergamasids (Bowman 2014, 2017), very rapid initial expansion of the gut occurs at the start of feeding commensurate with handling major essentially tissue-less fluid challenge on prey rupture, which would avoid any gnathosomal overspill.

Although circumcapitular volume and deutosternal volume increase together predictably in general over all mites (even when fractionated by cephalic type), could $\frac{circumcapitular\ volume}{deutosternal\ volume}$ be important? Consider that the deutosternal volume was estimated as $\pi .\left( \frac{AGW}{2}\right) ^{2}.GL$ and the circumcapitular volume estimated as $2\pi .\left( \frac{BGW}{2}\right) .AGW^{2}$ therefore, if

$$\frac{circumcapitular\ volume}{deutosternal\ volume}=6.3$$

which is the average over reviewed phytoseiids, then for possible BCAs

$$4.\frac{BGW}{GL}=6.3\quad \textrm{or,}\ \frac{GL}{BGW}=0.6.$$

Now the final equation is the deutosternal groove length as a proportion of the width of the *basis gnathosomatica*. From above, within the subcapitular model, overall subcapitular length matches *basis gnathosomatica* width on average. So this constraint looks like it has to do with a trade off between groove elongation (*GL*) versus overall subcapitular length. In other words how much it covers the ventral gnathosoma. On average over the reviewed mites as a set, *GL* is 61% of the length of the subcapitulum. So the phytoseiid design seems typical. Families with noticeably higher values are parasitids 0.73, macrochelids 0.79 and eviphidids 0.8. Those families with noticeably lower values are heterozerconids 0.51, megisthanids 0.46 and blattisocids 0.45. It would appear that having the typical design might be associated with being a useful BCA as this covers ascids, digamasellids, laelapids and rhodacarids some species of which have been tried out as agents (however, counterfactually arctacarids showed a value of 0.65). Intriguingly melicharids at 0.60, and parholaspids at 0.61 might be useful to further investigate as BCAs.

**Fig. 15:**
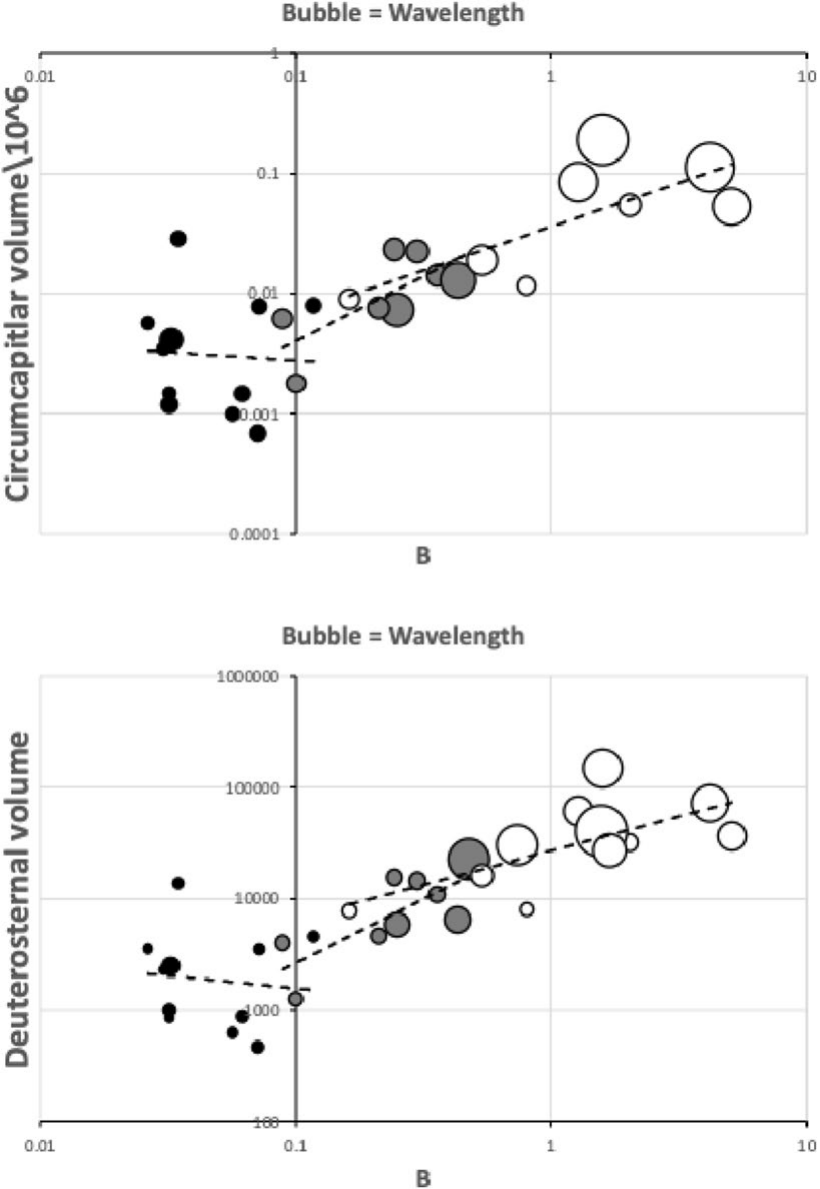
Compartmental volumes of fluids in $\upmu$m$^{3}$. (B) Volume available for prey fluids on a single cheliceral protrusion/retraction. All mites including *Uropoda abantica*. *Black dots* microcephalic species. *Grey dots* mesocephalic species. *Open dots* megacephalic species. Note small gnathosoma mites scale differently. Bubble size indicates wavelength of deutosternal transverse cross-bar ridges

Looking at the above result in another way, from the above regression results where increasing groove length while maintaining the same flow rate might indicate stronger pharyngeal pumping (so as to avoid the expected pressure drop in long grooves—see the Hagen–Poiseuille equation) and the *basis gnathosomatica* width broadly matching cheliceral shaft length, this would all suggest phytoseiids and rhodacarids have also narrower deutosternal pipes than their reach and their possible pumping mechanisms might suggest. In some species the slot-like appearance is so narrow so as to look like a thread (with the minimum observed $\tfrac{AGW}{GL}$ values of all the species reviewed herein). This in miniaturised species may need other structures to work effectively (see below). Phytoseiids and most rhodacarids may just not have the need for speedy transport of large fluid volumes. The converse of these conclusions apply to macrochelids and parasitids for instance which would certainly be consilient with their need to handle large fluid flows from say attacking large dipteran larval prey (in unstable environments like animal dung; Krantz 1998). This is not an issue of mites with proportionately wider than long deutosternal grooves being able to handle a proportionality larger volume in a single cheliceral retraction/protrusion per mite size (as there is no clear relation between $\tfrac{AGW}{GL}$ with $\tfrac{B}{V_{m}}$ for micro-, meso- or megacepahlic species, $R^{2}=0.0682, 0.0000, 0.1146$, respectively) but is about the *total* prey volume that may need to be handled. Indeed, Fig. 15 shows that small mites really are different. Whilst the circumcapitular buffer and deutosternal volume ‘gulp’ scale with the volume available for prey fluids on a single cheliceral retraction (B) for most mites, microcephalics (including all the phytoseiids studied) scale distinctly. They are hydrodynamically different.

A useful validation check in a follow-up study would be to compare the size of typical prey ($V_{p}$) with the initial predator volume ($V_{m,t=0}$). Single mites dealing with prey much larger than themselves on their own, would need a gnathosoma designed for high recycled coxal volume handling (like macrochelids and parasitids perhaps). Single mites dealing with prey one at a time markedly smaller than themselves on their own, might show a gnathosoma with limited coxal volume handling. Furthermore comparing $V_{p}$ to the volume available for prey fluids on a single cheliceral retraction (B) in a follow-up study may drive further insights. If one assumes that for each and everyone of the species studied herein, they only attacked prey of the same size as themselves (i.e., $V_{p}=V_{m}$, e.g., *Phytoseiulus persimilis*) then phytoseiids have the highest value on average (203) amongst the families studied herein for $\tfrac{V_{p}}{B}$ whilst parasitids have the lowest (59). That is, in phytoseiids little proportion of the prey fluid could be in and around the retracted chelicera (i.e., they are high chelal velocity ratio nibblers) and thus a trickle mechanism for liquid recycling may be sufficient. A check extending the work of Akimov and Starovir (see references in Bowman 2019) using a histological time series as in Bowman (2014, 2017) of starved phytoseiid abilities to rapidly increase their gut volume when challenged with *high* volume (i.e., not tetranychid) prey would be useful. Could this be why for instance *Phytoseiulus persimilis* has such a large globose idiosoma to accommodate fluid?

Flechtmann and McMurtry (1992b) recounts that *Iphiseius degenerans* and the *Euseius* spp. that they studied had wider deutosternal grooves (ca. $7{-}9\ \upmu$m) than other phytoseiid species which usually have $=4{-}6\ \upmu$m. It is possible that slot-like narrow deutosternal grooves in the latter may be about ensuring a continuous flow of fluids (perhaps by passive microchannel transport in the background) across the subcapitulum. The tritosternum in these species then perhaps being used to control the need for large intermittent recycling from time to time. This would be consilient with the relatively wide groove being posed by Flechtmann and McMurtry (1992b) as a modification possibly associated with the intake of the liquified contents of pollen grain cores. Inspection of a SEM of the black rat parasite *Laelaps nuttali* in Montasser (2013) (Fig. 2) shows a clear continuous slot from well under the tritosternum up to the fissures leading to the hypostomal tip/modified *malae* and labrum. This slot has a few longitudinal elongate spines in it. At a gross level these are streamlined like fish, dolphin (Fish 2006) and shark shapes. However, at a fine level they are reminiscent of the upper blades of tiger and mako shark skin riblets (Yunqing et al. 2017; Ibrahim et al. 2018) or the skin spines of sailfish (Tian et al. 2021). Hirschmann (1962) illustrates such central deutosternal spines in the blattisocid *Blattisocius tineivorus*, the laelapid *Hypoaspis (Pneumolaelaps) greeni*, and the two parasitic species *Rhinonyssus nitzschi* and *Pneumonyssus simicola*. Could these ensure continuous capillary flow like in the horned lizard water transport system (Comanns et al. 2015)? Continual serum or blood feeding would require continual fluid excretion much as phytoseiids drinking their prey (Flechtmann and McMurtry 1992a). In both parasites and phytoseiids there would be the need to keep the wound moist, so examining more parasitic mesostigmatids may be very useful. For sure central features (and the shape of these ‘ribs’) can alter fluid flows in rectangular micro-channels (Zhu et al. 2020).

*Laelaps agilis* is an ectoparasite associated with the *Apodemus* genus, which transmits *Hepatozoon* species via the host’s blood (Nazarizadeh et al. 2022). This species has a very elongate tritosternum clearly socketed behind the circumcapitular groove lying tightly over a narrow long deuterosternal channel. The tritosternal base encompasses only half the length of the *basis gnathosomatica* but the paired lacinae reach right up into the hypostome to the cornicular bases. Batwing parasitic spinturnicids lack a tritosternum or possess only a remnant (Dhooria 2016). Bird nasal rhinonyssids have tiny narrow many ridged deuterosternal grooves with seemingly one central tooth-like denticle per ridge row (Knee 2008). *Neomegistus remus* (Baker and Seeman 2008) has almost no span-wise ridges, just a long subcapitular traversing slot-like groove. *Neomegistus julidicola* is thought to feed upon fluids secreted by millipedes, examining the deutosternal function in kleptoparasites may also be useful. What is the situation in parantennulids (Kim and Castagnoli 2010)? Are there any parallels with phytoseiid structures? *Antennophorus* is a kleptoparasite (Owen Seeman *pers. comm.*) taking advantage of their ant carrier’s trophallaxis. Their *basis gnathosomatica* can be unusual (Wiśniewski and Hirschmann 1992) as are their membranous or micro-spiny corniculi. Indeed, many trigynaspid mites might do different things—scavenging, hunting, and stealing nutritious fluids (e.g., from prey if they are riding on a predator) are probably all in their ‘toolkit’. The well-studied parasitids on carrion beetles also seem to feed on various things, from being predators to somehow chewing-up carrion. Of course phytoseiids somehow handle pollen too. So while there is a link with food, some generalisation in function probably allows a degree of opportunism in feeding.

### Trophic efficiency and species comparability

**Fig. 16:**
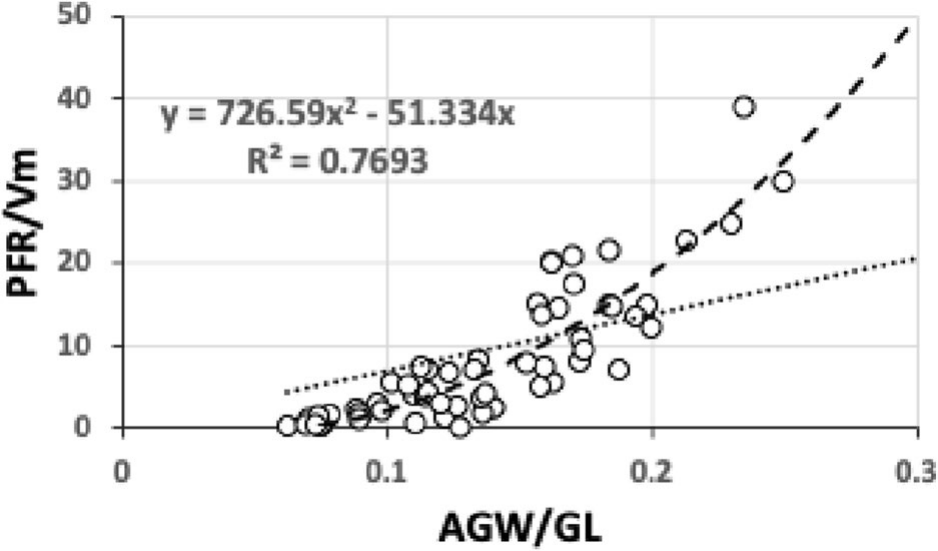
Mites (not including *Uropoda abantica*) with more broad than long deutosternal grooves have disproportionately bigger pipe flow rates per mite body volume (i.e., they show a ‘runaway’ process of design, dashed polynomial regression line through zero). Dotted line is poorer linear fit ($r=0.8535$)

Recall that $\frac{{\hat{tep}}^{*}}{V_{p}}=\frac{(AGW)^{4}}{GL}.\frac{1}{V_{m}}=\frac{PFRt}{V_{m}}$. In other words the flow rate relative to predator body volume for a deutosternal pipe is equal to the trophic efficiency of that mite per prey volume. The right hand term can be calculated for the mites species studied herein (Table 5). It is high in megisthanids. It is low in phytoseiids. It varies from species to species over the remaining families. Mites with relatively broad deutosternal groove design have bigger pipe flow rates per mite volume. It should and does linearly scale with deutosternal shape $\left( \tfrac{AGW}{GL}\right)$ quite well ($r=0.8535$, Fig. 16), since $PFRt=AGW^{3}.\left( \tfrac{AGW}{GL}\right)$, $V_{m}\propto IL^{3}$ and *AGW* is reasonably well correlated with *IL* ($r=0.6096$). This is a common-sense design effect.

However, an approximate square law is a much better visually fitting relationship. Allometry is occurring. The corollary of the latter point is discussed below. Now by definition (see above), $tep=\tfrac{V_{d}}{V_{m}}$ where $V_{d}$ is the total deutosternal volume over feeding. So $\tfrac{V_{d}}{(V_{m}.V_{p})}\propto \left( \tfrac{AGW}{GL}\right) ^{3} \Rightarrow \sqrt{\tfrac{V_{d}}{(V_{m}.V_{p})}}\propto \tfrac{AGW}{GL}$ i.e., broadening a deutosternal groove relative to its length for a mite of fixed size disproportionately even more increases the proportion of the prey volume that can be ‘stored’ as deutosternal groove ‘gulps’ during feeding. Broadening the groove indicates adaptations dealing with transporting (much) more water containing prey. Lengthening a deutosternal groove relative to its breadth (i.e., becoming like a pencil) has the opposite corollary.

**Fig. 17:**
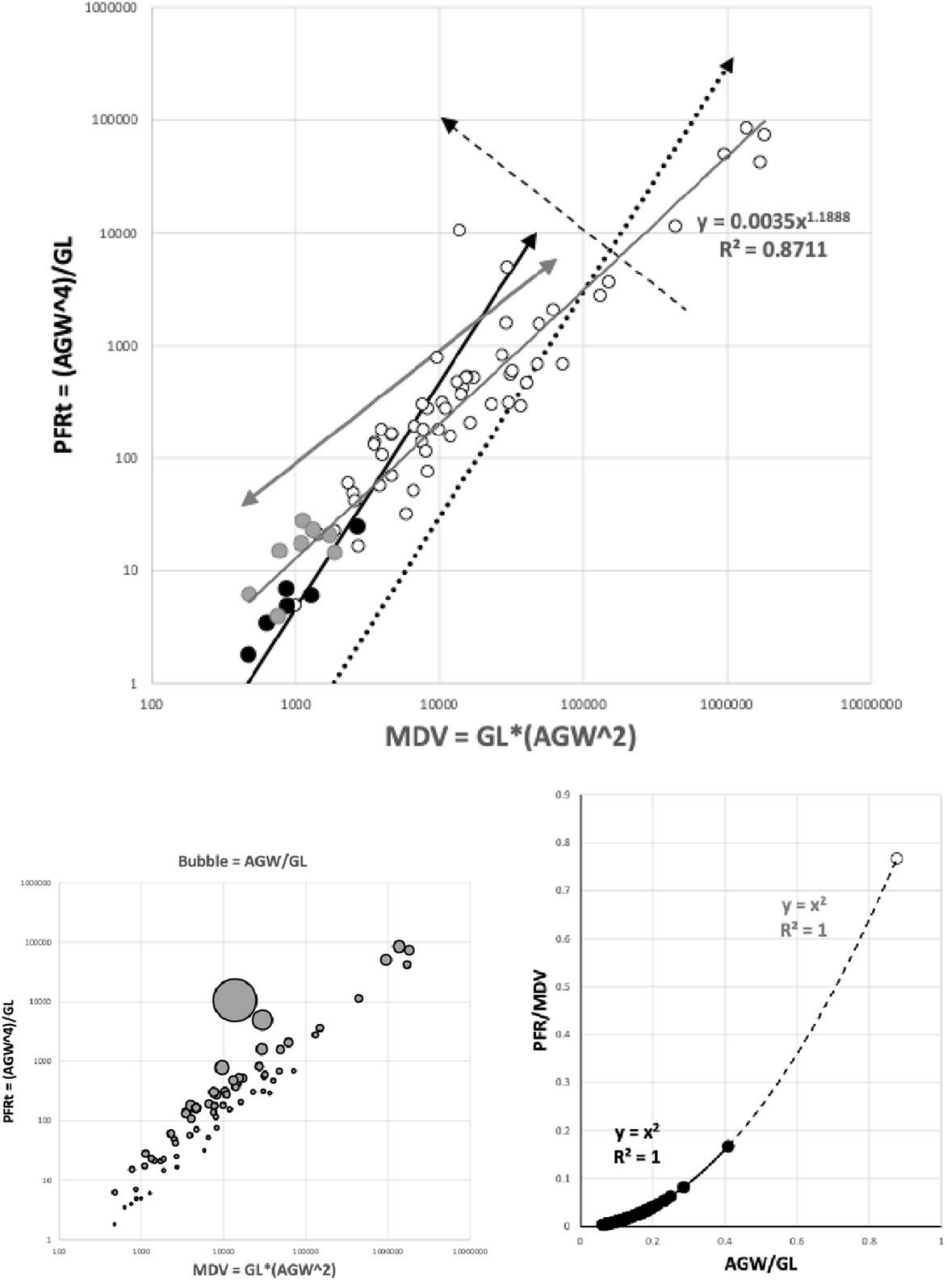
Mites including *Uropoda abantica*. Upper: *Black dots* phytoseiids. *Grey dots* rhodacarids. *Open dots* rest of studied species. Grey regression line shows good fit. Dotted line with arrow is fixed $GL=150\ \upmu$m and *AGW* changes $2{-}86\ \upmu$m. Dashed line with arrow is fixed $AGW=32\ \upmu$m and *GL* changes $10{-}500\ \upmu$m. Their crosspoint is approximately at the average [*x*, *y*] for all of the species reviewed. Double headed grey contour line is constant deutosternal aspect ratio $\tfrac{AGW}{GL}= 0.3$ (other contours omitted for clarity). Black single headed arrow is scaling phytoseiid design showing atypicality. Lower left: Species as one moves to upper left have ever increasing deutosternal groove aspect ratios in a square law sense. Lower right: Square law. *Open dot* *Uropoda abantica*. Black squares remaining species studied

This basic premise should not be a surprise. Firstly, define a deutosternal volume morphometrically i.e., $MDV\propto GL.(AGW^{2})$ which scales isometrically well with mite body volume ($V_{m}, R^{2}=0.9842$). Then, in Fig. 17 the spacing of the other contours of groove aspect ratio over the species (other than the single one drawn) will be arranged according to a square law because algebraically$\tfrac{PFRt}{MDV}=\tfrac{(AGW)^{4}}{GL}.\tfrac{1}{GL.(AGW^{2})}=\left( \tfrac{AGW}{GL}\right) ^{2}$. Studying other large mites and others with broad deutosternal aspect ratios in follow-up work should check if this algebraic level of overcompensation is empirically sustained.

The fluid pipe’s relative width is important in known BCAs, i.e. phytoseiids and rhodacarids have a design of relatively narrower thin pipes at any given length compared to other mites (or, alternatively a shorter length given their relative width). It is not clear why that should be so unless their prey volume shifting abilities are not needed due to relatively dry prey yielding modest coxal fluid secretions etc. (which would be consilient with the aforementioned observations by Flechtmann and McMurtry 1992a, and the fact that the *PFRt* values for microcephalics are lower than either their circumcapitular or deutosternal volumes would predict). Could this be why their tritosternum is in a depression and may therefore function differently? Could the tritosternal base being in the subcapitular depression (Flechtmann and McMurtry 1992a) be a different micro-droplet holding buffer mechanism to cope with anecdotal large prey fluid volumes from time to time?

Although the gnathosomal morphology of heavily sclerotised phytoseiids from grasslands needs examining more, Dave Walter (*pers. comm.*) recounts that phytoseiid gnathosomal morphology is aberrant compared to most soil predatory mesostigmatids. This being especially true of those species that seem most useful in conservation biocontrol. He recounts watching a *Euseius* species feeding on a pollen grain, there is very little fluid flow at all, and what there is comes from the mite and not its meal. Their gnathosomal ornaments are reduced dorsally (i.e., its tectum) and also ventrally with even the corniculi seemingly modified into a ‘butterknife’ shape. The tritosterna are pretty basic too. To the reviewer, all this is consilient with different hydrodynamics than say in macrochelids or parasitids. Indeed pollen grain feeding may generate no excess fluids at all, but actually require salivary liquids contributed by the mite, e.g., the laelapid *Pneumolaelaps longanalis* effectively removing ‘pollenkitt’ (Royce and Krantz 1989).

**Fig. 18:**
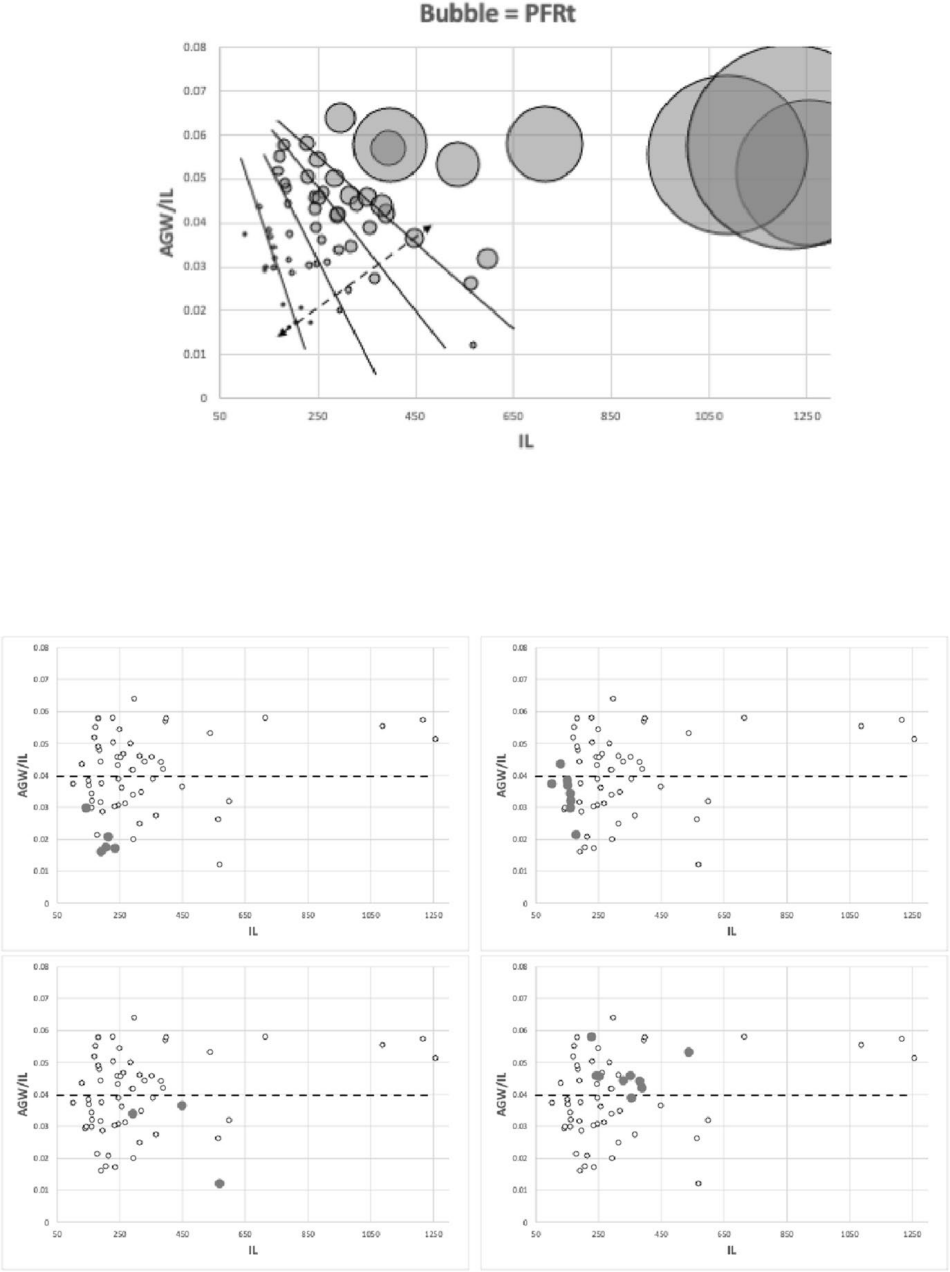
Mites including *Uropoda abantica*. *AGW* and body size are key characteristics for deutosternal pipe flow. *Upper*: Solid lines functional groups, sensu Walter and Proctor (2013), of approximately equal *PFRt*. Double headed dashed line approximately $y=a.x$ located on the phytoseiids roughly orthogonal to the functional groups. *Middle left* Phytoseiids in grey. *Middle right* Rhodacarids in grey. *Lower left* Parasitids in grey. *Lower right* Macrochelids in grey. Dashed horizontal line is average *AGW*/*IL* to emphasise lack of lower right hand quadrant species in each sub-panel

Deutosternal groove width (*AGW*) is a key dominant character in all the above relations. Body size is an important correlate of possible prey sizes that can be attacked. Large and more watery prey require large deutosternal pipe flow rates (and possibly more recycling). So, Fig. 18 shows their inter-relations using the species studied herein.

What does this show?

- Firstly there are no large size ($>650\ \upmu$m IL) relatively narrow deutosternal groove species. Is this a hydrodynamically ‘forbidden zone’?
- Secondly, parasitids and macrochelids have broadly similar deutosternal flow rates, yet vary in body size where smaller mites have a linearly bigger *relative* *AGW* i.e., as $AGW\propto IL^{2}$.
- Thirdly, phytoseiids and rhodacarids have broadly similar deutosternal flow rates, and also vary in body size where bigger mites have a linearly smaller *relative* *AGW* i.e., as $AGW\propto IL^{2}$ (with a different higher proportionality factor).
- Fourthly, a quadratic relationship between *AGW* and *IL* over all the mites studied (i.e., ignoring this change in proportionality constant from functional group to functional group) is supported by the whole data ($R^{2}=0.9039$).

So, this means that according to the ‘Bauplan’ that certain mite families within a functional group might share (perhaps due to their shared phylogeny), larger mites will have disproportionately narrower deutosternal grooves than simple linear growth compared to their *IL* suggests (i.e., their relative *AGW*/*IL* falls). This gives two equivalent options within a functional group: a smaller mite with a somewhat wider deutosternal groove (e.g., macrochelids); versus, a larger mite with a somewhat narrower deutosternal groove (e.g., parasitids) both with large flow rates adapted to deal with large volume and/or watery prey i.e., both being in the same functional group. Rhodacarids versus phytoseiids are another pair but designed for small drier prey—for such small mites their narrow *AGW* changes very little with body size. More example species of the very high *PFRt* value functional group are needed in follow-up work to discern equivalent families to the arctacarids etc.,but the same principle generally applies (though not to such an extreme since the lines flatten as one moves rightwards in Fig. 18 Upper, here *AGW* appears to scale linearly with *IL*). It is tempting to infer that parasitids are effectively repeat-feeding macrochelids in predatory habits, and phytoseiids repeat feeding rhodacarids. Further observations in the wild are needed. As an internal consistency check, the double headed arrow in Fig. 18 Upper, shows that if the deutosternal groove grows faster than *IL* (essentially moving across the functional groups), deutosternal pipe flow rate (*PFRt*) increases as expected. Further work is needed on ascid species with very wide oligodentate deutosterna such as *Cheiroseius samani* (Mehranian 2014).

### Height, shape and direction of ridges

For the most part deutosternal ridges in mesostigmatids are essentially linearly transverse, and arranged perpendicular to the groove length axis (like a ladder). Seven rows are claimed to be typical of gamasines (Flechtmann and McMurtry 1992b) although one phytoseiid they studied has 10–12 rows. The height of transverse ridges is important in influencing microchannel flow, above a critical height flow friction does not increase (Garg and Agrawal 2019). This height in mesostigmatids needs measuring in a follow-up SEM study. As does the shape of the span-wise secondary structure cross-sectionally (as whether they are square or semi-circular will impact any possible turbulence; Cervo et al. 2013).

In capillary rise, menisci are concave (equilibrium contact angle $\theta <90^{\circ }$), in capillary depression menisci are convex (contact angle $\theta >90^{\circ }$; Hensel et al. 2016). In 17 of the studied species, the transverse deutosternal ridges are essentially straight at the beginning and end of the groove. However, in *Macrocheles agilis* and *Macrocheles jabarensis*, for example, anteriorly near the internal malae deutosternal ridges can be concave crescent shaped, i.e., their centre portion is more posterior than their edge sections. This could impact the direction of capillary flow. Also, for example in *Macrocheles kaju* and *Macrocheles willowae* anteriorly they may be the opposite, i.e., convex crescent shaped. Similarly, posteriorly near the circumcapitular groove in for example *Gaeolaelaps jondishapouri*, they can be concave and in for example *Mucroseius insolitus* they can be convex. If one assumes that microchannel edge effects are important in defining the deutosternal fluid meniscus and flow (via $\varDelta p_{c}$), then forward movement towards the hypostomal tips is facilitated in a further 23 of the reviewed species (to deplete the circumcapitular reservoir). Backward flow would be facilitated in a further 14 reviewed species (thus filling the circumcapitular groove). Only one species (*Anntenoseius bregatovae*) showed transverse ridge shape that might disperse a centrally held subcapitular droplet both forwards and backwards (Karg 1965 illustrates a similar situation accompanied by a pear-like shape to the longitudinal edges of the deutosternal groove in *Eugamasus eta*). Seven species showed a transverse ridge arrangement that might focus a droplet on the subcapitulum in the groove centrally, e.g., *A. wisniewski*, *Antennoseius pyrophilus* and others. No clear pattern with family was observed. All six phytoseiids show incomplete transverse ridges centrally, so that the groove had just lateral ribs making the whole assembly look like one of the rectangular ribbed microchannels in microfluidic heat sinks (Wang et al. 2015; Patil and Wangdare 2018; Zhu et al. 2020). This would facilitate a central channel flow of an even narrower pipe-like fluid thread.

Now, crescent shaped micro-structures facilitate fluid flow over fish skin, producing beneficial drag reduction even at low speeds (Tian et al. 2022). V-shaped ribs are known to increase mixing in rectangular microchannels (Wang et al. 2019). Given pharyngeal pumping as a fluctuating pressure source for the microchannel (Nandi and Duari 2022), and denoting this pressure alternately as ‘sucking material into the labral area’ versus ‘pushing material into the circumcapitular buffer store’, then

- Mixing anteriorly in and around the bases of the internal *malae* could be facilitated on pharyngeal sucking if anteriorly the deutosternal ridges are convex but posteriorly the ridges are concave (e.g., in *A. wisniewski*, *A. pyrophilus*), or on pharyngeal pushing if anteriorly the ridges are concave but posteriorly the ridges are convex (e.g., in *A. bregatovae*).
- Mixing posteriorly in and around the junction with the circumcapitular groove could be facilitated on pharyngeal pushing if posteriorly the ridges are concave but anteriorly the ridges are convex (e.g., in *A. wisniewski*, *A. pyrophilus*), or on pharyngeal sucking if posteriorly the ridges are convex but anteriorly the ridges are concave (e.g., in *A. bregatovae*).

It is worth noting that the pattern of convexity and concavity of transverse ridges varies in other *Antennoseius* species (Gwiazdowicz and Haitlinger 2010). Validation of this mechanism by biomimetic fabrication is needed. An entry point for custom microchannel manufacturing techniques is Kandlikar and Grande (2003). Recent laser patterning methods can be found in Liu et al. (2020).

### Number of deutosternal ridges and ‘vibrating fluid’ in the groove channel

Over the female species studied, the number of ridges (including both ends, real or notional) varied from 2 to 14 (average of 7.8). There was scant evidence of any relationship with groove length ($R^{2}=0.0191$) nor with groove width ($R^{2}=0.0803$). Parasitids and arctacarids had the highest number of ridges on average (11.6 and 10.7, respectively), megisthanids the least (5.3). Phytoseiids showed 8.5 ridges on average over the species studied. There was scant relationship to deutosternal tubification (as measured by $\tfrac{AGW}{GL}$, $R^{2}=0.0283$) although relatively elongate grooves had a higher number of ridges.

A linear surface of transverse bars (like a deutosternum) could capture and hold a large amount of fluid and through surface tension maintain a film of liquid across itself—consider the circular ‘bubble wands’ that children use dipped into a soapy solution and then pulled sharply through the air to make bubbles. Here the passing changing air pressure pulls at the liquid making it move and change shape. For the purposes of investigating the comparative primary and secondary subcapitular structure across mites, one could pose as a ‘thought experiment’ that the deutosternal ridges indicate some sort of mechanism to entrain a vibrating wave within fluid in the groove. This is not so exotic a notion as it sounds as certain energy dissipation conditions can lead to an oscillatory regime of laminar flow in microchannels (Yarin et al. 2009). Furthermore, for instance when a liquid flowing over a surface hits a step protruding up from the substrate fluid oscillations occur (King and Bloor 1987). Ridges may be such steps. Further, when flowing thin-film liquids encounter a step-down in substrate level flow heights temporarily change (Kalliadasis and Homsy 2001; Saprykin et al. 2007). So perhaps the location of subsequent ridges represent the existence of natural frequencies in the deutosternal fluids which are easy to excite into standing waves (or droplet-like ‘bulges’). This could be by alternate pharyngeal fluctuations or even perhaps by repeated flexure of the gnathosoma at the idiosomal joint or its periodic retraction as a whole. It is not at first clear how this is trophically advantageous, but what would be the corollary of this possible resonance?

Firstly, if one considers that the spacing of the deutosternal ridges (i.e., the peak-to-peak distance between Querleisten) may mark the nodes of a fluid vibration in the channel, i.e., they (on average) define the distance between two wave crests (or similarly between two wave troughs), then this defines an observed wavelength ($\hat{\omega }$, Table 5). Over the species studied this was $17.9\ \upmu$m on average. There was little evidence that exactly the same process determining wavelength occurs over all mites, as this wavelength increases both with groove length ($r=0.6588$) and with groove width ($r=0.6917$)—*Diplothyrus lehtineni* being an atypical outlier. Larger mites (which tended to be mega-cephalics) have a bigger value for their deutosternal wavelength than smaller mites. However, the wavelength relative to *IL* is similar across most of the families studied (except for the phytoseiids where this is somewhat small), This suggests a possible common modality of formation or handling for deutosternal bulging ‘droplets’ of fluid even though their size is different between species. Recall the results above that *AGW* appears related to $IL^{2}$ i.e. so a fluid droplet spanning the deutosternal groove width should have a surface area coverage $\approx \pi .\left( \tfrac{AGW}{2}\right) ^{2}$ if of regular minimum surface area shape. Indeed, if one considers each ‘cell’ of the deutosternum ‘ladder’ to be estimated by the surface area $Wavelength * AGW$ i.e., the droplet is not exactly regular, this then scales as a quadratic function with mite idiosomal length (*IL*) well over the species reviewed $R^{2}=0.9539$. Each almost square cell thus containing a possible bulge of fluid which if the chitinous surface was hydrophobic could form a discrete sub-spherical droplet, bigger mites having exactly predictable bigger deutosternal ‘droplets’. There is no obvious reason why one would get such a regular arrangement if the function of ridges was simply to mechanically scrape tritosternal lacinae clean of debris as posed by Wernz (1972).

**Fig. 19:**
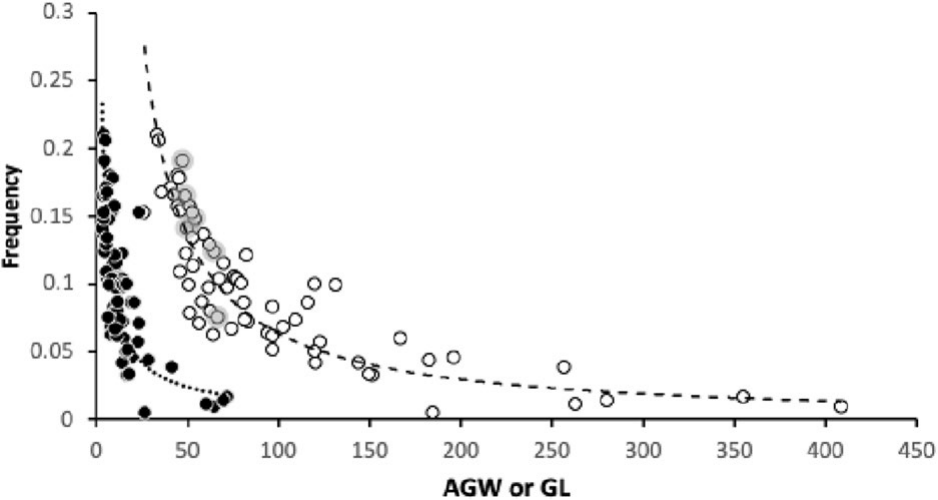
Deutosternal ridge frequency versus average groove width (solid dot, dotted fit) and groove length (open dot, dashed fit) with indicative power regressions. All species studied including *Uropoda abantica*. Note essential ‘broken-stick’ linearity: $<25\ \upmu$m average groove width versus $>25\ \upmu$m average groove width, or $<75\ \upmu$m groove length versus $>75\ \upmu$m groove length. Grey dots are phytoseiids

If one assumes that the speed (*s*) at which any fluid wave travels is broadly the same between mite species. Then without loss of generality the observed frequency $\hat{f}\propto \tfrac{1}{\hat{\omega }}$. Indeed the observed frequency consiliently shows both a power relationship with groove length ($R^{2}=0.7424$) and with groove width ($R^{2}=0.5551$) indicating a broadly ‘broken-stick’ linear relationship—Fig. 19. As a musical analogy, longer deutosternal grooves match a lower frequency note, narrower deutosternal grooves a higher frequency note. Note that as expected, the rate of change in frequency is highest amongst the small groove lengths or small groove widths, and the rate of change lowest amongst the large groove lengths and large groove widths. As Bowman (2020) says “Small size matters”.

Now, the fundamental oscillatory frequency of an ideal string (the real stiffness of which can slightly affect the frequency) fixed at both ends is

$$f_{1}=\frac{1}{2L}\sqrt{\frac{T}{\left( \frac{m}{L}\right) }},$$

where *m* is the mass of the string of length *L* and *T* is its tension. The mass of a uniform *circular* cross-section string of radius *R* and length *L* is $m=L \pi R^{2} \rho$ where $\rho$ is its *volume* density since the cross-sectional area of such a string with radius *R* is $\pi R^{2}$. This means that

$$f_{1}=\frac{1}{2LR}\sqrt{\frac{T}{\pi \rho }}$$

or for pipe-like strings of the same length of the same density experiencing the same tension, the thicker string will have a lower fundamental frequency. Harmonics follow as integer multiples of $f_{1}$. The majority of the range of calculated mite frequency values (Fig. 19) falls between 0.05 and 0.2, i.e., a fourfold difference, this would encompass the first 3 harmonics beyond the fundamental frequency suggesting a polyfunctional redundant design to the deutosternal layout in high ridge frequency species like phytoseiids (i.e., they can also behave like a lower frequency design) or run at different speeds.

Water has a density of $994\ {\text {kg}}/{\text {m}}^{3}$, animal tissues range from 812 to $1178\ {\text {kg}}/{\text {m}}^{3}$ (https://itis.swiss/database/density). So, given essentially the same density of the subcapitular fluids across mesostigmatid species (i.e., as being either prey tissue overspills and/or excreted coxal fluids), and assuming the same fundamental tension in this analogous ‘string material’, then the above relations with the observed frequency should infer that $\hat{f_{1}}\propto \tfrac{1}{GL*AGW}$. Indeed, there is a good linear relationship of observed frequency with this above righthand side term (with or without the megisthanids and the holothyrid, $r=0.8219$ and $r=0.8146$, respectively).

There is therefore reasonable evidence that the deutosternal ridge arrangement is to do with perhaps reinforcing a channel of vibrating fluids (or engendering patterns of drag suppression at a certain scale—see Bandyopadhyay and Hellum 2014). Further that this result would be consilient with the small groove length, narrow groove width species being designed to shift low density fluids (e.g., water, dilute solutions, coxal fluids etc.) and the long groove length, wide groove width focused on a design to move high density fluids (e.g., prey liquidised tissues etc.). This would suggest prey overspill is more likely in macrochelids and (only) coxal fluid transport more likely in phytoseiids. Again detailed observations of macrochelid feeding may be relevant. Polyfunctional species would be adapted to do both and thus deal with small and large volumes of both prey fluids and coxal secretions. Again as Bowman (2020) found smallness engenders strict specialisms. Whether also flexibility in feeding habits is related to size awaits further investigation. Whether the ability to be an opportunistic feeder also engenders particular sub-capitular differentiation remains yet to be seen.

**Fig. 20:**
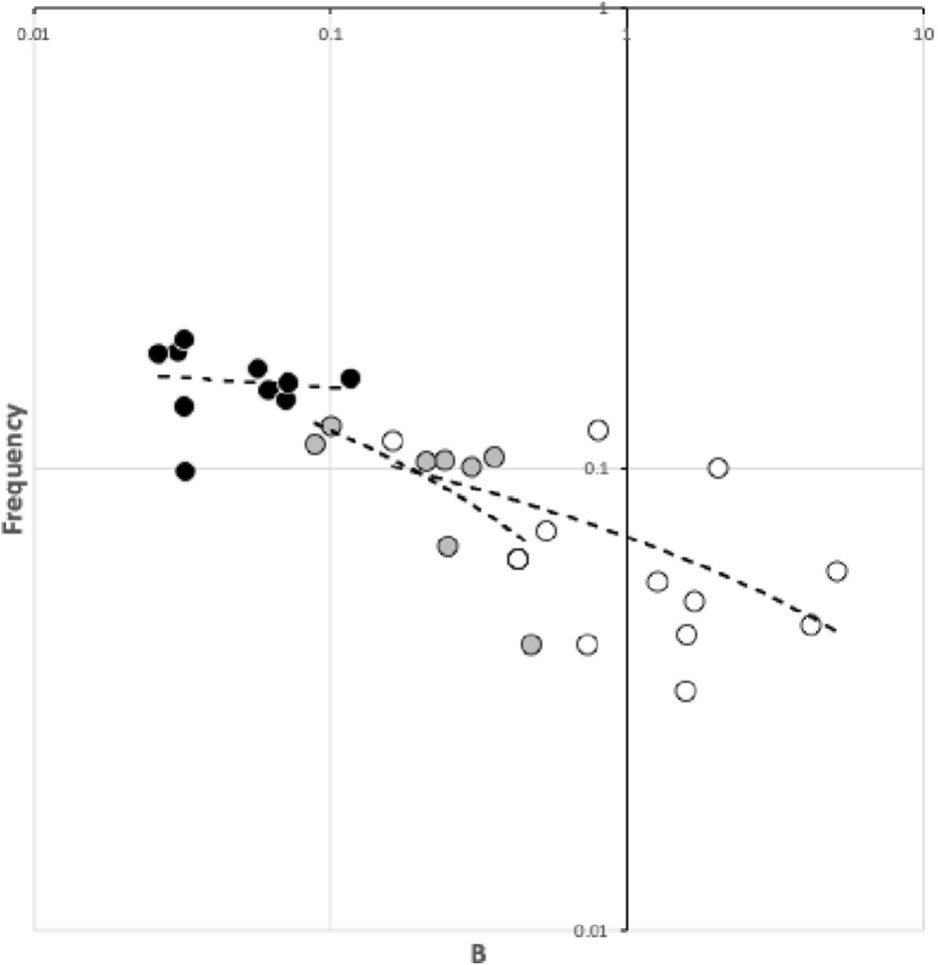
Deutosternal ridge frequency versus volume available for prey fluids on a single cheliceral retraction/protrusion (B), all mites not including *Uropoda abantica*. Note limiting frequency for microcephalic mites. *Black dots* microcephalic species. *Grey dots* mesocephalic species. *Open dots* megacephalic species. Indicative logarithmic regressions as dashed lines

However this all means that if the deutosternal groove is long (i.e., in an elongate gnathosoma mite) high frequency ridges only occur in thin microchannel species, i.e., microcephalic mites who may be water handling specialists. Conversely, in ‘snub nosed’ megacephalic mites with *per force* relatively short deutosternal grooves, paucity of ridges (i.e., a low frequency) only occurs in wide channel species (e.g., *Megisthanus* spp.). Hence there is a lack of any relationship with the width–length aspect ratio *AGW*/*GL* (omitting uropodids) with either the number of ridges ($R^{2}=0.0302$) or the deutosternal ridge frequency ($R^{2}=0.0988$) despite including the unusually designed *Macrocheles forceps*. Phytoseiids as a group are particularly unusual as a family in having such low ratios. As microcephalics they show a limiting frequency with declining values of volumes available for prey fluids on a single cheliceral protrusion/retraction (Fig. 20). Perhaps this is indicating a switch in these high frequency low *PFRt* species to a different kind of fluid flow (e.g., crawling central threads in a narrow microchannel of variable speeds)? Larger mites then having a single speed of subcapitular fluid transport ?

**Fig. 21:**
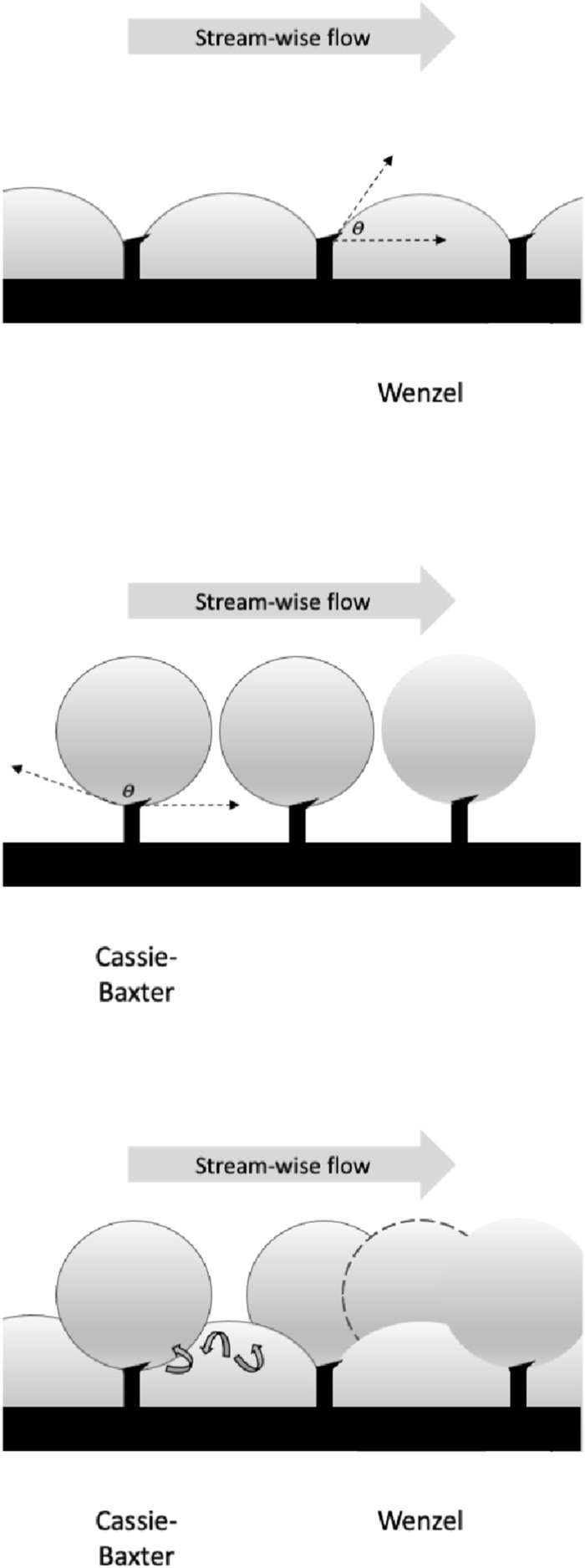
Suggested fluid transport mechanism in mesostigmatid deutosternal groove. *Upper* wetted basal layer of fluids in Wenzel state between span-wise ridges (‘Querleisten’ Hirschmann 1962). *Middle* fluid ‘droplets’ in Cassie–Baxter state notionally ‘stick’ above denticulated deutosternal ridges (which may act as pinning barriers). *Lower* resultant ‘droplets’ undergo speedy pumped transport (i.e., de-pinning as necessary) and ‘slide’ as bulges over a stationary/slow moving Wenzel state lower boundary layer. Denticles act as vortex generators to expedite flow. Note exaggerated distinction between Cassie–Baxter and Wenzel states for ease of exposition. Fluid edge boundaries shown for clarity of exposition. Dashed edge is bulge passing over inter-ridge ‘cell’ (i.e., in act of effective sliding). The fluids in Wenzel state and those as droplets are in fact completely continuous ‘vibrations’ as in far right hand bulge. Size of fluid droplet bulge approximately matches inter-ridge spacing (and indeed *AGW*)

This vibration analogy would be consilient with fluid drops being in a Wenzel state between the longitudinal deutosternal edges as they transit the planar inter-transverse ridges ‘cell’, but become raised into a Cassie state when they encounter the next micro-denticulated transverse ridge (and then sit on top of the denticles, hence the variation in the number of denticles across species). The droplet essentially pulsating up and down as the fluid contact angle changes ($\theta$ high to low) in its posterior–anterior subcapitular transit (Fig. 21), pinning and de-pinning (Ceyhan et al. 2020) as necessary. The Wenzel layer acting like a boundary layer. The footprint of the sub-spherical droplet thus inferring that *AGW* should scale quadratically with *IL* (which it does). The denticles could then act as low-profile vortex generators (Domel et al. 2018) to expedite this transport above. If indeed turbulence does occur at the scale of deutosternal groove flows then the apparent bulges at the above ridge wavelengths could be due to the formation of secondary flows over the ridge type of span-wise heterogeneous roughness (i.e., example (a) in Fig. 17 of Kadivar et al. 2021). Further, if any turbulence is occurring then subcapitular cuticular stiffening might facilitate span-wise vibrant wall drag reduction for the stream-wise flows (see Yunqing et al. 2017). Cuticular stiffening then obviates considering compliant wall models and fluid flow (see Bandyopadhyay and Hellum 2014; Tian et al. 2021). The relationship of groove edge absence/presence and their lengths to body size needs investigating. Further investigation is also needed to see if the deutosternal cuticle does indeed vary in its hydrophobicity to synergise this postulated mechanism. Of note is that denticle width does not appear to be related to deutosternal flow rate (*PFRt*) suggesting that any effect of vortex generators is related to the number of the finlets not necessarily their size. One could think of this lower Wenzel state layer as effectively acting like liquid infused textured surfaces do (see Kant and Pitchumani 2020, Sabdullah et al. 2020). Perhaps the mite might already have permanently lubricated the surface with something in advance of fluid flows? More follow-up work is needed.

Secondly, could rather these conclusions be driven by accidental morphological coincidences dependent upon some function that changes phylogenetically? If the deutosternal ridges were directly related to the number of oral gutter depressor and/or pharyngeal opener muscles (that insert on the ventral chitinous surface inside the mite above them), then the former should increase with hypostomal length (i.e., approximately with moveable digit length) and the latter with the length of the *basis gnathosomatica*. Thus under this assumption, the number of ridges should scale with subcapitular length. However, it does not (overall $R^{2}=0.0315$). Furthermore, anecdotal evidence e.g., in zerconids Ujvári (2011) shows pharyngeal muscle attachments on the venter of the subcapitulum out of phase with deutosternal ridges. Only if one omits the megisthanids then does this hypothesis have some modest evidence supporting it herein ($r=0.869$). Pointing perhaps to fluctuation in these muscles being the origin of the fluid waves in the smaller mites, with a less strengthened cuticle, whether directly (or by resonance through the gnathosomal fluids). However, the latter species are more likely to depend upon microchannel based hydrodynamics than pumped micro-pipe based fluid transport. Such resonances would, of course, be affected by the degree of openness or closedness of the gnathosomal liquid cylinders controlled by the tritosternum and their interconnectivity to other fluid holding compartments in and around the gnathosoma. However, one then must pose say what advantage a longer labrum gives in some species, why some species might have many more pharyngeal effectors, etc. The idea that deutosternal morphology directly matches pharyngeal musculature appears unlikely.

So, the suggestion herein is that the ridge pattern is to facilitate the movement along of semi-sessile droplets (‘bulges’) held in certain parts of the deutosternal groove (via a coupled discrete ‘stick-slide’ process) over a morphologically induced boundary layer. Periodic pharyngeal pumping moves bulges of fluid from being pinned at each ridge to the next ridge. Cuticular cross-hatching, that is ‘ladder-like’ strengthening in a narrow structure, would be exactly what one would expect if a designer needed to stiffen a surface. Is this why megisthanids show tessellations that seem to match a possible plesiomorphic fusion of two sternites to form the hypostome/*basis gnathosomatica*? Their cuticle is probably already stiff due to their size. Longitudinal ridges prevent excessive anteroposterior bending of the cuticle as the pharyngeal contraction wave sweeps posteriorly internal to the gnathosoma. Note that longitudinal edges are missing (Karg 1965) in many pergamasids, eugamasids and veigaids presumably with already well sclerotised stiff ventral surfaces. The transverse Querleisten ridges would prevent expansion of the venter of the subcapitulum surface laterally. Wernz (1972) describes (at least the teeth on) the deutosternal surface as ‘flexible’. The transverse ridges and the longitudinal edge ridges would stiffen the venter of the already chitinised subcapitulum therefore like struts. Micro-mechanical investigations are needed.

Imagine for a moment, each ‘cell’ in the ladder-like groove design might encompass a single sub-spherical water droplet (consiliently over all the female mites studied herein $wavelength=1.2123 \times AGW\, (\upmu \text{m}),\ R^{2}=0.6149$, if males and nymphal stages are included then $wavelength=0.8854 \times AGW (\upmu \text{m}), R^{2}=0.328$). Then rapid alternation of the pharyngeal muscles anchored interiorly on this surface might also cause this stiff floor to oscillate inducing resonant vibrations in any fluid droplets (Yoshiyasu et al. 1996). Wall vibration is a method of active drag reduction in fluid flow (Marusic et al. 2021; Tian et al. 2022). Of course, fluid ‘sloshing’ would absorb energy and dampen down the vibrations (Alum and Dickerson 2021) of any droplet sustained at the top of the asperities. However, at the right resonance, asymmetric small amplitude vibrations (of the reported flexible membrane deutosternum; Wernz 1972) could induce droplet movement horizontally via a ratcheting mechanism as shown in some model systems (Dong et al. 2006). Fluid would then flow in semi-discontinuous ‘blobs’. The increased deutosternal cross-bar wavelength in mites with longer grooves (*GL*) would be consilient with this if the size of the sessile droplet was bigger for bigger mites. This needs experimental validation but should be the case as the frequency of vibration for a water drop is $\propto \sqrt{\frac{S}{\rho .r^{3}}}$ where *S* is surface tension of liquid, $\rho$ is density and *r* is the radius of the droplet. So for constant speed and surface tension across mites $wavelength \propto r^{3/2}$ which for say a droplet as wide as the deutosternal groove, would suggest $wavelength \propto AGW^{1.5}$. A relationship that fits the observed data reasonably well ($R^{2}=0.5499$). When a droplet of water is placed on surfaces patterned with parallel, periodic grooves, they have been shown to wet anisotropically to the surface elongating the shape of the drop (Temperton 2012). Under vibration these elongate droplets can show standing waves on their major and their minor axis (Temperton and Sharp 2013). Effects like this may account for the not quite square shape of each deutosternal groove ‘cell’. Asymmetric vibrations can move sessile drops on a horizontal hydrophobic surface (Dong et al. 2006). In model systems vibrated droplets can even climb uphill (Brunet et al. 2007). Indeed at low volumes could the deutosternal edges and transverse ridges be designed for micro-scale droplet handling, and the denticles for separate nano-scale droplet handling much like the hierarchical function of structures on termite wings (Watson et al. 2010)? Or are the denticles just an ancillary mechanical cleaning system as the lacinae and their pili scrape over them as described by Wernz (1972)? A flat surface (like the posterior dorsum of the tritosternum and vertically standing denticles would facilitate that. Further, species with no need of deutosternal cleaning (including specialised fluid feeding parasites) should lack teeth. So does this infer that phytoseiids do not deal with heterogenous prey material? Biomimetic simulations are needed.

Three corollaries arise if this suggested mechanism (Fig. 21) is the case.

(i) The deutosternal wavelength should be well correlated with *AGW* (i.e., $AGW=0.9165*wavelength$ $r=0.9149$ omitting megisthanids and the neothyrid).
(ii) The deutosternal groove length should reflect the overall length of the pharynx internally. This could be checked by micro-dissection or a series of histological cross-sections in a follow-up study. Di Palma et al. (2006) appears to illustrate pharyngeal constrictor muscles in *Veigaia* sp. only above part of the gnathosomal venter (see Fig. 9 therein).
(iii) The partial or full integration of the groove with the hypostome and/or the *basis gnathosomatica* over different species should reflect where the pharynx sits above it for that species. In particular any swop in deutosternal external architecture along its length (such as in Fig. 23) reflects the various internal oral musculatures. For instance the most anterior portion being where the oral-gutter depressor muscles or anterior pharyngeal dilator muscles (*adm* in Starck et al. 2022) are above it, the medial deutosternal portion being where the ventral pharyngeal dilator effector muscles (*vdm* in Starck et al. 2022) are above it, and any gap posteriorly under the tritosternal base before the circumcapitular groove arises having no muscles above it at all. The circumcapitular groove being affected only by gnathosomal flexure/protrusion/retraction (in anactinotrichids muscles attach to the gnathosomal base). Examining larvae, protonymphs and deutonymphs as well as adults of the same species (e.g., Cómbita et al. 2018; Ujvári 2011) versus other mites of the same size could be very illuminating here as it is posed that external architecture should reflect notional pharyngeal pumping power, prey type (and thus fluid volume), etc.

Note that a follow-up study could also check how the length of the gnathotectum (and flexible epistome; Wernz 1972) relates to the length of the subcapitulum. If one fixed the deutosternal cell frequency over all mites as the same, then the speed (*s*) of fluid movement would be proportional to the wavelength (i.e., $f=\tfrac{s}{\omega }$). That would mean the ratio of speeds for different fluids at say $f=1$ would be $\frac{\omega _{max}}{\omega _{min}}$ which for the species reviewed herein would be $\approx 39$. In other words a 40-fold speed increase between the smallest and largest mites. Can water move substantially quicker than thick prey fluids given the same pumping pressure? Experimental validation is needed to check if this is reasonable.

Finally for the completion of possible options, if the deutosternal groove fluid system were rather to be matched to a mass–spring system (i.e., as a fluid pulse of mass *m* is displaced by *Q* units one way then relaxed), then the solution for its sinusoidal movement over time is $x(t)=Q.\cos \left( \sqrt{\frac{k}{m}}.t\right)$ where *k* is the massless spring constant. Each single displacement could be by the alternate action of a chelicera causing volume B to slosh over the gnathosoma. This volume has an inertia to overcome, however, there is no obvious way to estimate the cheliceral retraction/protrusion force. Nevertheless, this alternative model of the deutosternal pipe/channel acting as a simple harmonic oscillator means that irrespective of being a horizontal or vertical system, a higher fluid bolus mass would lead to a smaller frequency (as $f=\tfrac{1}{2\pi }.\sqrt{(}\tfrac{k}{m})$). In this case a shorter frequency over species would represent a stiffer spring. A plot of frequency versus $GL.(AGW^{2})$ shows poor linearity ($R^{2}=0.2162$ overall, on omitting megisthanids $R^{2}=0.1467$) i.e., the opposite. The ridges do not echo a mechanism of just shifting bulk masses (i.e., bulk fluid volumes) to and fro. So, it still appears that the deutosternum is designed for one-way fluid transport.

### Karg’s trends of hypostomal form in free-living gamasids

Karg (1965) offers an interesting table of hypostomal forms, focusing upon their morphology with particular reference to Querleisten (deutosternal cross-bars). How might these work hydrodynamically? He categorises mesostigmatid species firstly by the degree of sclerotised fusion with the pedipalp coxae (viz. “Hypostom weichhäutig” versus “Proximale Sklerotisierung” versus “Fast vollständige Sklerotisierung"). Proximal here meaning with respect to the idiosoma. Mechanically this axis must be confounded with mite size (larger Acari are invariably more sclerotised) and also with the forces involved in moving chelicerae in and out and those delivered by the cutting/crushing action of chelal digits (i.e., *F*2) during prey handling. Although for at least his genera listed under “Ursprüngliche Form mit der Querleisten $Q_{1}-Q_{8}$”, extraction of the *IL* and *F*2 values for the nearest species in Bowman (2020) does not obviously confirm this. A strong gnathosoma would be needed to handle actively struggling prey like fly larvae. Note that Karg appears to regard some mites with $L_{x}$ (i.e., “Längstlesiten”, like the weakly sclerotised *Prozercon* and *Zercon*) to be included in the basal form. He also allows various ridges to be oligodont, polydont, lengthened (“länger”) or absent (“frei”) in this type.

The weakly sclerotised basal form encompasses various other genera: *Leioseius*, *Asca*, *Typhlodromus*, *Arctoseius*, *Antennoseius* in part, *Lasioseius* in part, *Protogamasellus*, and *Rhodacarellus*. Some of these are used as BCAs. This suggests that substantial subcapitular differentiation may not be a prerequisite for BCA success (in other words the Proposal in this review might not be the case). The proximally sclerotised basal form is shown by other *Antennoseius*, and other *Lasioseius* species as well as in *Epicriopsis* and *Proctolaelaps*. Furthermore, *Iphidozercon* and *Sejus* typify the almost completely sclerotised forms. It is tempting to conclude that these sclerotised forms might be just ‘beefed up’ variants of a possible BCA design useful for larger tougher prey than say tetranychids.

Karg’s other categorisation (cross-classifying the above) is to contrast:

- Cross-bar $Q_{1}$ being present, being repeated (“$Q_{1}$ wiederholt verkütz”), and a proximal reduction in Querleisten, versus
- A distal reduction in cross-bar $Q_{1}$, development of additional supernumerary ridges (“Zusatzleisten”), and a distal secondary formation of a $Q_{x}$ ridge.

Some of the second type (b), e.g., many *Pergamasus* spp., *Poecilochirus*, many *Eugamasus* spp., *Saprogamasus*, *Gamasolaelaps*, and *Veigaia*, show proximally a multiplication of cross-bars (“Vermehrung der Querleisten”). This seems to suggest that extra hypostomal based fluid flows are of importance in these mites’ feeding hydrodynamics. Other mites Karg lists showing a similar trend (but varying in details) are the weak or proximally sclerotised *Rhodacarus*, *Gamasellus*, *Sessiluncus*, *Saprolaelaps*, *Saintdidieria*, *Saprosecans*, * Leitneria* and *Ameroseius*. At least the last genus is mycophagous. A curious form is the sclerotised *Gamasiphis* (Ologamasidae), this has “Längsleisten” ($L_{x}$) which suggests it is hypostomaly adapted more like the pergamasids and parasitids.

The first type (a) above comprises the weakly sclerotised: pachylaelapids *Pachyseius* and *Pachylaelaps*, eviphidid *Alliphis* (and the almost completely sclerotised *Evimirus*), laelapid *Hypoaspis*, digamasellid *Dendrolaelaps*, plus the macrochelid genus *Macrocheles*. The soil-based *Hypoaspis miles* (= *Stratiolaelaps scimitus*) is a natural predator of fungus gnat pupae. *Stratiolaelaps* spp. are already used commercially as BCAs. This suggests that perhaps deutosternal adaptations behind this type of differentiation (i.e., possibly shorter but more posteriorly abbreviated grooves) may be of interest for new mesostigmatid agents.

### Ancillary subcapitular structures

**Fig. 22:**
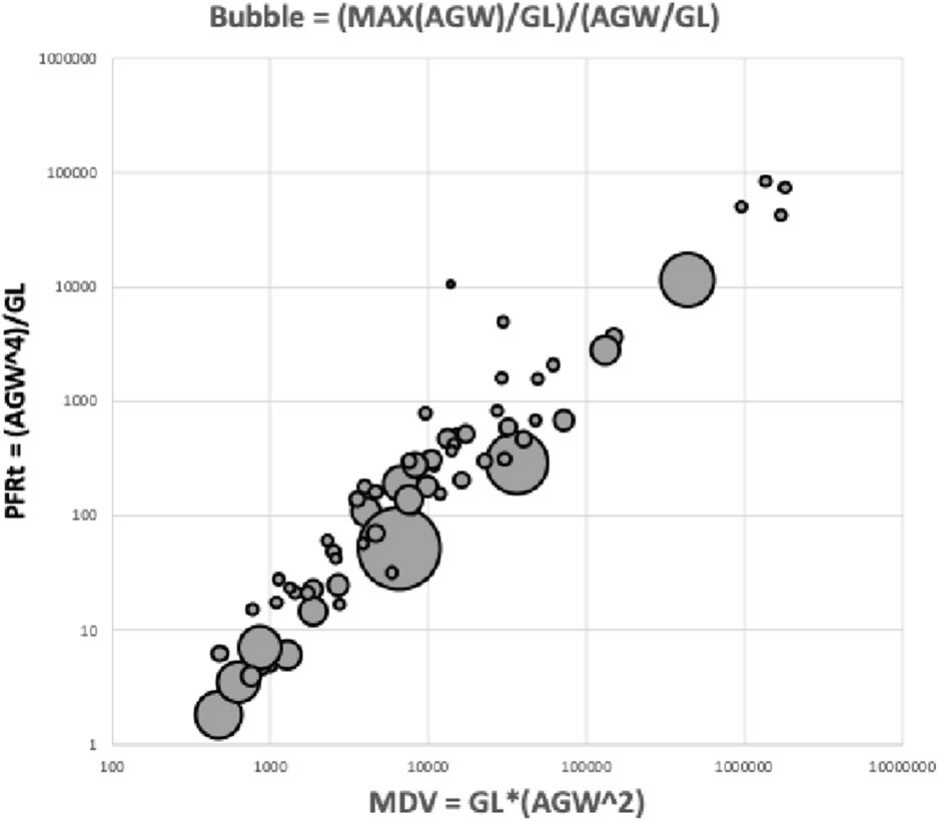
Facultative feeding design, all mites including *Uropoda abantica*. Note situation for all phytoseiids (lower left group). Most taxa can augment their narrow deutosternal groove geometry. Compare to Fig. 17

Figure 22 shows that effective widening of the deutosternal groove by ancillary ridges etc., occurs throughout mites of different morphometric deutosternal volumes and deutosternal pipe flow rates. Geometry matters. Offering more routes for fluid flow both reduces the pressure it is under and its velocity (Liang et al. 2019). More total fluid can be accommodated. This facultative feeding option occurs at all mite sizes, in all functional groups and is particularly pronounced in phytoseiids (lower left group).

**Fig. 23:**
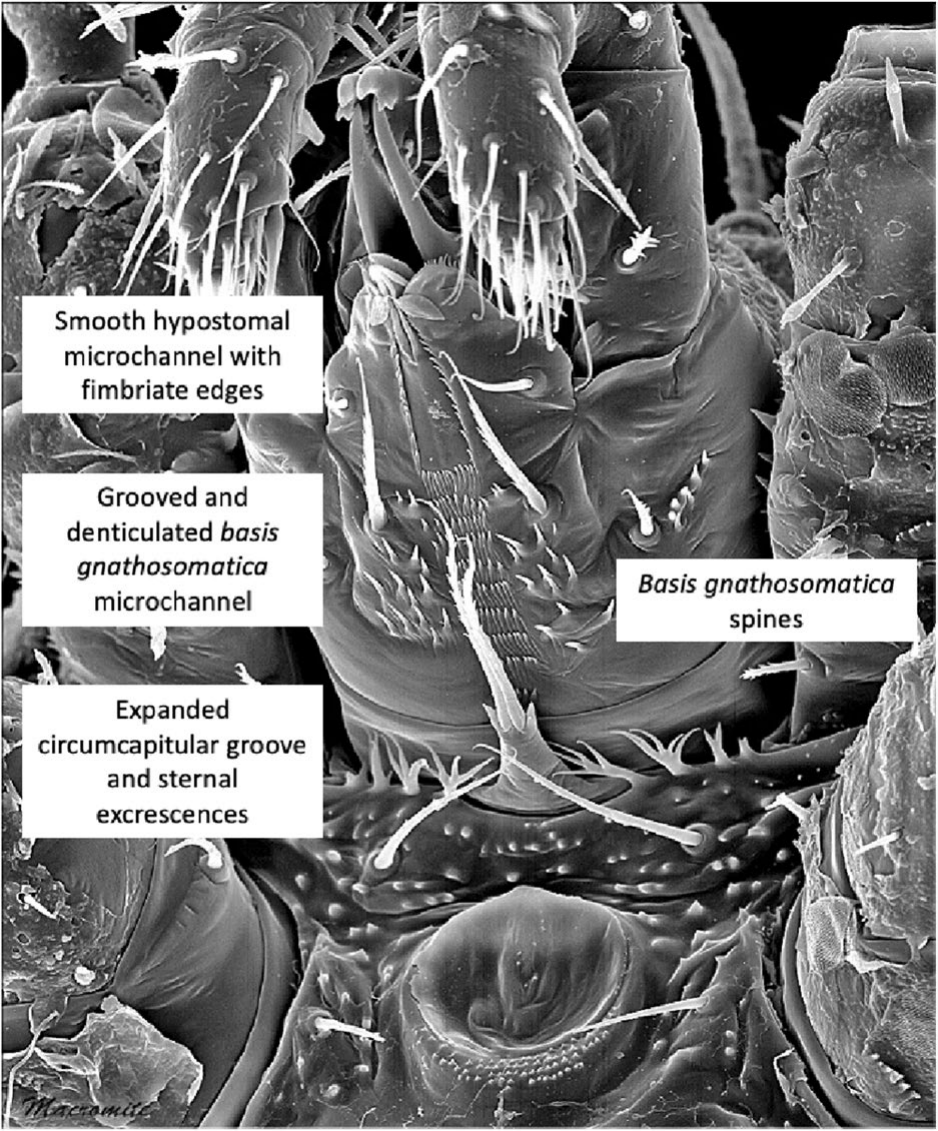
Venter of male sejid showing a variety of deutosternal groove adaptations. Features lateral of subcapitular groove may facilitate handling of unusual amounts of fluids from opportunistic predation of atypical prey. Exact function may change according to how extended or retracted the whole gnathosoma is as the tritosternum moves relative to the subcapitulum. Here the anterior smooth surface channel sits in the fluid ‘ring’ of Wernz and Krantz (1976). Setose sternal excrescences may increase circumcapitular buffer volume. Subcapitular surface roughness changes left-right and anterior–posteriorly. Amended from photograph of male *Epicroseius* sp. ex An Empty Shell (April 25, 2015) by Dave Walter at https://macromite.wordpress.com/2015/04/25/an-empty-shell/ with permission

Figure 23 displays many of these adaptations. Such could be a useful design for the efficient handling of unpredictable types of prey (i.e., in generalist versus specialist predators). Sternal excrescences with their regular spacing and setose/fimbriate nature could effectively increase the volume of fluid held behind them (by surface tension) without evolution requiring a deepening of the circumcapitular groove through a change in gnathosomal socketing with the idiosoma. Whether the latter is airorhynchid (typical gamasine) or klinorhynchid (typical uropodoid; Bowman 2020) will also matter. Acarologists should not only consider optimal designs that there may be for regular typical prey feeding but also morphological contingencies to facilitate trophic adaptation. Could trying a transform-roughness correlation approach as used in understanding drag in rivers (Adams and Zamprion 2020) upon contours of estimated surface roughness over the whole subcapitulum be a way to start?

Where along the groove the lateral extensions are, may matter. Hirschmann (1962) illustrates three scenarios:

(i) Basal subcapitular denticles (*Zr* in the ameroseiids, and the heterozerconid *Allozercon fecundissimus*). This looks like a particular overflow mechanism to expand the subcapitular groove fluid buffer for fungivores, and specialists associated with wet millipede frass and litter.
(ii) Major denticulated ridge areas laterally (*Zr*1, *Zr*2, *Zr*3, *Zr*4) in the discozerconid *Discozercon mirabilis*, the epicriid *Epicrius mollis*, and the heterozerconid *Heterozercon audax*. These are assumed to be an adaptation to opportunistically hold extra fluid volumes as films across the subcapitulum if and when necessary (or to act with the groove as a gross oral overflow ‘wick’).
(iii) Anteriorly positioned extensions in the uropodoid *U. cassidea*. These are presumably forming a hypostomal overflow or temporary buffer mechanism anteriorly. Could such be the cause of the small ‘hypostomal droplet’ sometimes observed by Wernz (1972)? Is this what the forward-pointing denticles sometimes covering the hypostomal setae $h_{1}$ in *Antennophorus* are also doing? This species is probably feeding directly from sugary droplets disgorged during trophallaxis of their ant carrier (Owen Seeman *pers. comm.*). Perhaps these denticles are doing all that they can do to keep that droplet from breaking and flooding over the hypostome (see Wiśniewski and Hirschmann 1992 for a description of all *Antennophorus* life stages)?

Uropodoids are different again. *U. cassidea* has ancillary denticle fields in the posterior of the deutosternum, and *Uropoda virgata* has only extensive denticle fields over its deutosternum (Hirschmann 1962). Denticle fields also appear at the sides of the deutosternum in the parasitic *Rhinonyssus nitzschi* and *Pneumonyssus simicola* (Hirschmann 1962). Could such surface roughness act as pillars for a super-wetting mechanism to ensure expedited flow of fluids in a Wenzel state? For sure these parasites live in ‘wet’ conditions.

Zerconids show a denticulated grooved deutosternum (e.g., *Zercon triangularis*; Hirschmann 1962) that makes the excrescences look lobate (Ujvári 2011). Whilst arranged in serried rows (in *Zercon peltatus* and *Prozercon fimbriatus*; Karg 1965) rather than diamond-like tessellations, these look like the microstructures on Galapagos shark skin (Bechert and Hage 2006) known to facilitate stream-wise flows. Such dense arrays of longitudinal ridges (“Zähnchen durch Längsleisten verbunden”; Hirschmann 1962) can also be found in the ologamasid *Gamasiphis sextus*, the polyaspidid *Polyaspis sansonei*, the sejid *Epicroseius angeloides*, and the uropodoids *Uroseius (Apionoseius) infirmus* (Trachytidae), and *Trichouropoda dialveolata* (Trematuridae). What could be the common denominator in feeding mechanism between these taxa requiring such hierarchical channel architecture? Indeed the different structures at different levels in the hierarchy (like in springtails; Hensel et al. 2016) could have different surface properties leading to droplets in different combinations of Wenzel and Cassie states at different scales occurring.

The number of transverse denticulate ridges across the deutosternal groove being usually seven is the case among numerous other families, no matter the width or dentition of those ridges, e.g., Ascidae, Blattisociidae, free-living Dermanyssidae and Ameroseiidae (Evert Lindquist *pers. comm.*), In many an anterior-most smooth border or transverse rim is also present. Dave Walter (*pers. comm.*) notes that the deutosternal groove and internal malae are separated from the hypostome by a suture (in some mesostigmatids). This was described in the historical literature, where some have argued that this area is derived from the sternites of putative segment II and hence its name. How this smooth border or rim or suture might impact fluid movement is not clear.

Euzerconids, paramegistids, parantennulids and klinckowstroemiids (Womersley 1958) would be worth examining more. Especially given for instance the striking morphological similarity of klinckowstroemiids to fedrizziids (Seeman 2000).

Palp trochanters can have unusual protuberances of unclear function too (Faraji et al. 2021). Owen Seeman (*pers. comm.*) recounts that the setae on the trochanter, and on also other segments, can be heavily barbed (see for example Fedrizziidae). They look like they have a cleaning function, but in SEMs they appear to support unusual corniculi. On the other hand, setae on the palp femur look like they could clean the fixed digit, or maybe serve as guides. Fedrizziidae have large palp trochanter processes which do not look like they would touch the chelicerae, but perhaps they could do in life? These long excrescences must take some managing, being used to sweep fluids from tiny carcasses (like a brush or mop). Pairs of several vertically orientated ‘denticles’ on the margins of the phytoseiid deutosternal groove are illustrated by Flechtmann and McMurtry (1992b). Strangle scale ‘denticles’ are also mentioned in Flechtmann et al. (1994) rather than groove ridges. Specialist phytoseiid predators have various numbers of (nano?) denticles on various numbers of rows of these scales (Flechtmann and McMurtry 1992b). Cheliceral lobes may function as fluid tubes in some phytoseiids to steer fluids to the oral cavity (Flechtmann and McMurtry 1992b). Indeed, phytoseiid deutosterna exhibit considerable variation (Flechtmann and McMurtry 1992b). How all of this impacts feeding hydrodynamics needs follow-up work and testing via micro-fabrication.

### Ridges as a micro- or nano-structure

A follow-up study using micro-fabrication is needed to properly ascertain the detailed function of the longitudinal ridges along deutosterna especially to understand their (nano-scale?) infoldings (Wernz 1972). Some mites function apparently without them e.g., *Zygoseius papaver*, yet close relatives collected at the same place e.g., *Zygoseius lindquisti* have them (Ahadiyat and Beaulieu 2016). They could function as cuticular surface level changes to pin the edges of the deutosternal droplet/bulges as they flow forwards. Few or any longitudinal groove edges were recorded by Hirschmann (1962) for: the ascid *Platyseius major*, two ologamasids *Hydrogamasus littoralis*, *Cyrtolaelaps mucronatus*, three parasitids *Saprogamasus ambulacralis*, *Eugamasus hyalinus*, *Eugamasus eustructurus*, the veigaid *Veigaia nemorensis*, the halarachnid *Halarachne halichoeri*, and two uropodoids—the trachyuropodid *Trachyuropoda elegantula* and the uropodid *Uropoda virgata*. Yet the subcapitulum in these species must still hydrodynamically function effectively somehow.

For sure, increasing the area of flow by the groove widening posterior–anteriorly will cause a velocity drop in the fluid moving forward (i.e., as in a divergent duct, like that illustrated for the probably fungivorous *Ameroseius gracilis* by Karg 1965). This diffuser effect may also be achieved by the supernumerary transverse hypostomal denticulated ‘cross-bars’ $Q_{x1}$, $Q_{x2}$ arranged anteriorly of the deutosternal groove proper in the euseiid *Euseius hyalinus*, or the supernumerary $Q_{x}$ in the macrochelid *Macrocheles subbadius*, most ologamasids, most parasitids, the rhodacarid *Rhodacarus roseus*, the sejid *Epicroseius angeloides*, and veigaids (see Hirschmann 1962). In that way these are functioning like extended tritosternal lacinae would to guide microchannel fluid flow forward.

Compensation for the lack of channel edges anteriorly might be the function of the longitudinal anterior $L_{x}$ in ologamasids *Euryparasitus emarginatus*, *Ologamasus rotulifer* and the parasitid *E. eustructurus* (Hirschmann 1962) possibly acting beyond where the lacinae might reach. They could also indicate lateral fluid micro-flows and thus facilitate mixing inside any fluid diffuser region. Their height and orientation needs measuring in a follow-up SEM study.

The opposite situation applies for convergent flows. Supernumerary transverse arranged denticles at the posterior of the deutosternum were illustrated by Hirschmann (1962) in *Cyrtolaelaps mucronatus*, *Eugamasus hyalinus*, and *Typhlodromus wichmanni*. Convergent denticulated rows with no longitudinal edges were illustrated by Karg (1965) in pergamasids, eugamasids and some *Veigaia* species. All of these structures could act as the inverse of a diffuser (i.e., a gathering ‘concentrator’ of fluids flowing anteriorly) reinforcing the function of the opposable tritosternal base (and perhaps supporting an even bigger droplet resting under it when necessary like an extra buffer). *Poecilochirus carabi* (Parasitidae) appears to have an hourglass shape to its deutosternal groove (Karg 1965) which may facilitate holding droplets at either end.

Some species have a systematic decreasing trend in the width of deutosternal cross-bars as one moves posterior-anteriorly, e.g., *Platyseius serratus* (Hirschmann 1962). Transverse ridges in a microchannel can change fluid flow in two distinct ways (Gaddam et al. 2018). The resistance (or pressure drop, $\varDelta p_{c}$) to transport a liquid through a microchannel decreases rapidly with its characteristic length. One way to reduce this pressure drop is by texturing the inner surfaces of the microchannel. Surface texturing causes the liquid to exist in one of two states. As pointed out above, these can be described by either liquid flow over entrapped vapour in cavities formed by the textures, known as the Cassie–Baxter state, or penetrative flow with the liquid filling the cavities formed by the textures, referred to as the Wenzel state (Hensel et al. 2016). In the former, a reduction in the pressure drop is due to the velocity slip at the liquid–gas interface. A reduction in pressure drop in the latter is ascribed to an increase in the effective flow area. Theory suggests that the heterogeneous wetting Cassie–Baxter state offers a lower resistance to the liquid flow than the homogeneous wetting Wenzel state barring certain conditions (cf. compare fast ‘pipes’ versus slow channels).

Although no survey of the deutosternal surface has been reported to include a basal vapour layer, a possible drop in fluid resistance next to the subcapitular cuticle may be how the deuterosternum is designed to function. The deutosternum is a groove textured by its ridges which it is proposed herein could behave as a microchannel and/or a micro-pipe. The longitudinal ridges restrict the lateral but enhance longitudinal spreading of fluids to form a basal layer. Being in a Wenzel state this allows filament propagation of fluids along edges which then merge across the rectangular cells of the deutosternum. Indeed the particular arrangement of small amplitude subcapitular ridges and grooves appears to be a designed element to manipulate the shape of fluid menisci over long ranges (see Johnstone et al. 2022). In the postulated mechanism herein, the transverse denticulated ridges, in turn, ensure that the liquid film between the longitudinal edges remains macroscopically stable, thereby creating a continuous basal ‘slippery track’ when little volume is present. This hierarchical ridge structure would thus render the whole subcapitulum wettable for droplets/fluid bulges centrally, and the water films continuous above it, so avoiding the need for a strongly hydrophilic surface chemistry, which would attract detrimental contamination. Flow over a surface with evenly spaced, finite-depth rectangular grooves can be regarded macroscopically as being equivalent to a smooth surface with partial slip (Wang 2003; slip length is explained in Bhushan 2011). This partial slip condition can then expedite the flow through a channel where it mimics the effects of superhydrophobic surface (Tan et al. 2020). For more details regarding flow on hydrophobic surfaces containing a periodic array of longitudinal and transverse micro-grooves under Poiseuille flow see Teo and Khoo (2009). Given that the ridge to ridge wavelength in mites is quite high, i.e., the texturing is sparse, and no deutosternal ‘silvering’ by a vapour layer in a subcapitular ‘plastron’ has been observed by acarologists, the scale of any Cassie–Baxter–Wenzel effect (as is detailed further below) is expected even if present to be modest but still potentially useful. Independent experimental results on flow past biomimetic textured microchannels (e.g., Gaddam et al. 2017) especially constructed at this mesostigmatid scale is needed as experiments are not always in agreement with the theoretical calculations (such as can be found in Gaskell et al. 2010; Alireza and Floryan 2011). Game et al. (2017) is a good entry point to follow up on this literature. Incomplete transverse ridges (‘ribs’ and ‘cross-ribs’ Chen et al. 2022) may still improve flow performance in a microchannel (Zhang et al. 2022). Even offset incomplete ribs ($\equiv$ serrated channels; Chang and Zhu 2020) are another possibility to consider as useful in mites.

The heuristic mapping of denticles used in this review yields that over the 72 species studied: $no.\ denticles = 1.1662 \,*\, no.\ ridges$ ($R^{2}=0.4670$). Taking the 91 (different) taxa listed in Hirschmann (1962) and deriving an average number of denticles individually counted by him over the transverse Querleisten for each species (ignoring $Q_{x}$, $Q_{x1}$, and $Q_{x2}$) yields a very similar relationship over all of those species: $no.\ denticles = 1.0361 \,*\, no.\ ridges$ ($R^{2}=0.5708$), and strongly overlapping data plots (*not shown*). Furthermore the groupings of parasitids, macrochelids, rhodacarids and phytoseiids between the two studies are in broadly similar positions on a $2 \times 2$ plot. The heuristics are therefore validated. It would appear that approximately 1.1 denticles are associated with 1 ridge as the base case for mesostigmatid designs in general, although wide variation occurs.

Flechtmann and McMurtry (1992b) recount that phytoseiids have one to four, most commonly two, denticles per ‘row’ ($\equiv$ ridge). They claim that in some phytoseiid species one or two of the basal ‘rows’ (i.e., numbers six and seven, using their terminology) have pairs of several vertically oriented denticles on the margins of the deutosternum. To this review author, these appear to be simply flattened wider teeth compared to other rows. In *Phytoseiulus longipes*, Flechtmann and McMurtry (1992b) claim that these ‘rows’ resemble overlapping scales (rather than discrete denticulate ridges). To this review author, there appears to be also scale-like extensive ridge extensions laterally of the groove in many of the phytoseiids that they illustrate. The two *Phytoseiulus* species studied by Flechtmann and McMurtry (1992b) differ from all of the others in that for *Phytoseiulus fiagariae* all seven deuterosternal ‘scales’ bear 3 to 5 (sub)denticles on their anterior margin while for *Phytoseiulus longipes* it has 10 to 12 scales each bearing up to 5 (sub)denticles. Furthermore, *Galendromus occidentalis* differs from other species in that it has only one (sub)denticle on ‘rows’ one to five and two (sub)denticles each on six and seven. The (sub)denticles may or may not function like other mesostigmatid denticles. The review author is restricting the term denticle to be those clearly on Querleisten. In either case hydrodynamic characterisation with biomimetic fabrications is indicated.

**Fig. 24:**
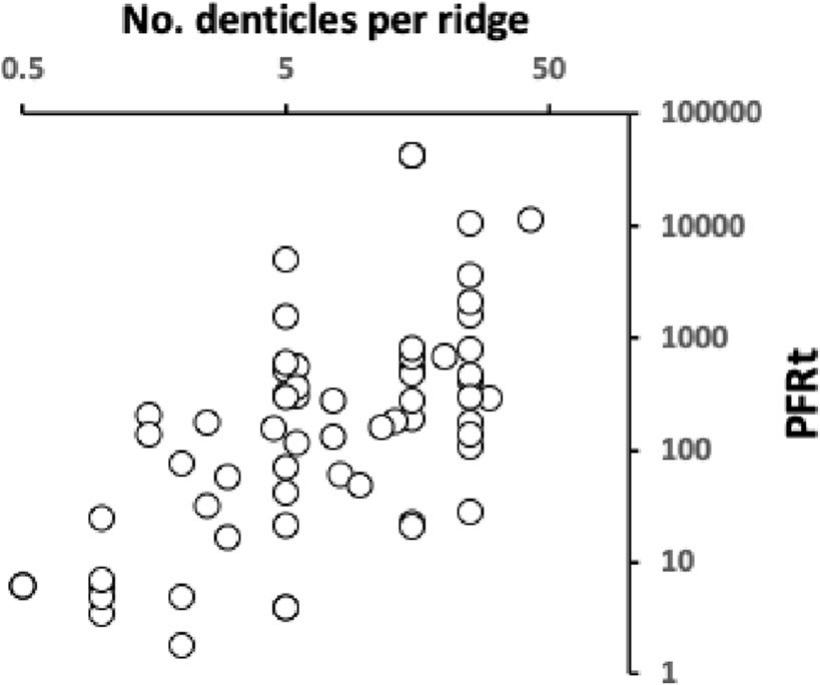
Of those species showing denticles, higher deutosternal flow rates (*PFRt*) are positively associated with more average denticles per transverse deutosternal ridge

For the study herein, there is only a mild relationship of the number of denticles with deutosternal groove width ($R^{2}$ through zero $=0.3129$). Some species showed no denticles on their span-wise deutosternal ridges: *Arctoseius cetratus*, *Philippinozercon makilingensis*, *Gaeolaelaps gillespiei*, *Megisthanus modestus*, *Megisthanus southcotti*, *Megisthanus simoneae*, *Diplothyrus lehtineni*, *Multidentorhodacarus matatlantica*, *Multidentorhodacarus saboorii*, *Multidentorhodacarus tocantiensis*, and *Multidentotrhodacarus squamosus*. The number of denticles per deutosternal ridge may be related to mite family. Phytoseiids appear to have low numbers as Walter and Proctor (2013) outlined already, macrochelids high for instance. Exactly why the latter occurs is not clear unless larger fluid flow might require more turbulence suppression (i.e., vortices in the lower semi-Wenzel state boundary layer Fig. 21). Overall $no.\ denticles=0.8209\, *\, wavelength\,(\upmu$m) $R^{2}=0.5821$. It would appear that larger ‘bulges’ e.g., in macrochelids might require more turbulence suppression? However, it is not clear to the review author exactly how vortex generators scale in practice. Vortex generators on wind turbines are orders of magnitude smaller than the turbine blade length, so small denticles in mesostigmatids are consilient. Figure 24 supports this drag suppression idea in general for those species with denticles as larger numbers of denticles per ridge are, for sure, associated positively with higher estimated deutosternal flow rates (*PFRt*). Alternatively if Querleisten denticles were designed to encourage ‘pinning’ of droplets (Theodrakis et al. 2021) on the cross-bars (Fig. 21 Middle), then larger Cassie state droplets might need more contact points? Flow over ribs can induce three different types of turbulence depending upon the depth of the flowing liquid, the height of the barriers and the gap between them (Shamloo and Pirzadeh 2015). More investigations on differential wettability, detailed simulations and biomimetic constructions are needed.

### Ridges as ‘mixers’

Could the deutosternal ridges control fluid mixing (Howell et al. 2005) instead? They effectively produce an up-down waviness to the microchannel, known to affect pressure flow and fluid velocity at certain frequencies (Kieger 2021). Increased mixing is drag enhancement. Chaotic eddies that homogenize fluids in turbulent flows are completely absent in a laminar flow. Rather, fluids follow smooth streamlines, which remain side-by-side even in meandering channels. There would be little mixing between distinct gnathosomal streamlines, so two fluids flowing in a standard microchannel will not mix with each other, except via a very slow diffusion at their common interface. Under laminar flow conditions in any gnathosomal micro-channel, rapid mixing is thus not easily achieved. So at the postulated low Reynolds number, low deutosternal flow rate assumed from low cheliceral protrusive/retractive or pharyngeal pumping power (or low $\varDelta p_{c}$), if more fluid mixing (say of prey fluid overspill with coxal fluids) than that caused by slow molecular diffusion responding to a concentration difference was trophically advantageous then extra mixing techniques would be needed in such a microchannel (Tan et al. 2020).

Passive mixing techniques which do not require an additional external mechanism to drive the mixing process are the alteration of the surface topology (e.g., grooves and rib patterns), changes in the channel geometry or relying upon the hydrodynamics of the flow as expected over the subcapitulum. Indeed, using small angled features, such as slots, oblique ridges, or chevrons, one can generate transverse flow motions that effectively reduce the diffusion length and increase the contact surface between the liquids. So, bas-relief, herringbone (Stroock et al. 2002) and chevron ornamentation in microchannels (and therefore on the subcapitulum venter around the deutosternum) facilitates chaotic mixing (Chen et al. 2011). Perhaps the relatively wide spaced deutosternal ridges (with the their positively raised denticles whose points might shed vortices) are to facilitate mixing of prey fluids and coxal secretions through inducing more off-axis, lateral or turbulent flow?

Denticles may function to increase drag by increasing turbulence (e.g., by shedding vortices at their tips) to facilitate fluid mixing in the essentially low pressure laminar flow in mite gnathosomas. Indeed, requirements for fluid mixing may therefore be related to the deutosternal footprint perhaps? So full integration of the subcapitulum with the channel as in phytoseiids would suggest little mixing, partial integration (and $L_{x}$ structures; Hirschmann 1962) more mixing. Are the tips of individual denticles exactly aligned anterior–posteriorly as one moves from ridge to ridge? Or, are they offset or even in random arrangements much like in the insect ectoparasitic otopheidomenid *Nabiseius palifer* (Joharchi et al. 2022b)? This will affect the friction coefficient (Sirovich and Karlsson 1997) and degree of mixing. Follow-up work is needed.

When fluid moving forwards or backwards encounters an angled groove, a transverse flow component is generated, as there is less resistance to the flow in the direction parallel to the groove. As a result, the fluid will start to follow helical streamlines, slowly rotating along the longitudinal axis of the channel. A 45$^{\circ }$ angle characterising the orientation of the ridges with respect to the channel (much as is found at the edges of the deutosternal ridges near the internal *malae* in *Neopodocinum longisetum* or near the circumcapitular groove in *Proarctacarus* spp.) is the most appropriate (thus acting like a sort of ‘rifling’). Is this what curved Querleisten are doing? Serrations trailing backwards in the direction of moving air over aerofoils stabilises air flows downstream reducing wake turbulence (Liu et al. 2016) and also improve wind turbine blade performance (Llorente and Ragni 2020). Deutosternal denticles facing anteriorly would facilitate flows therefore from ‘cell’ to ‘cell’ as fluids depart the circumcapitular groove and move towards the internal malae. Vortex generators (https://en.wikipedia.org/wiki/Vortex_generator that look exactly like the deutosternal denticles in shape and tiny relative form are widely used to stabilise flows around wind turbine blades by reducing flow separation (Zhao et al. 2022). Their symmetric raised form acting similarly in either rotational direction. Between span-wise ridges like in the deutosternum, the existence of vortices in the semi-Wenzel state ‘valleys’ could reduce fluid drag above them, functioning as an anti-friction bearing (Fu et al. 2017). Note however that rows of denticles can act also as blockages rather than vortex generators in reducing the intensity of stream-wise turbulence (Boomsma 2015).

Indeed, for a chevron (or herringbone) pattern, if two flowing fluids are present, they end up being inter-layered, which reduces the diffusion length. Chaotic advection of laminar flows does require peculiar geometries of channels (Tóth et al. 2015). So, could one regard the overall arrangement of dentate ridges (and periodic lateral extensions—see species marked * in Table 1) to be a widely spaced type of staggered micro-herringbone pattern designed at driving a *particular* sort of flow (and mixing; Williams et al. 2008) of fluids derived from different gnathosomal locations. Flows over irregular topologies can have surprising effects (Stam 2003). Micro-observational follow-up of particular species is needed.

Why would such species prioritise fluid mixing? As this mixing is deterministic, the mixing introduced by a given sequence of grooves or chevrons can be reversed using an inverse sequence (Howell et al. 2008; Hashemi et al. 2010). Periodic interconnected half-open capillary channels (including D-shaped chambers) control passive uni-directional fluid flows amongst super-hydrophilic skin scales of the snout of Texas horned lizards (Comanns et al. 2015). So, is it thus possible to read the sequence of likely activities as one follows any postulated path of fluids around a mesostigmatid mite’s chitinous cuticular gnathosomal structures? Is this what is happening when the shape of the deutosternal ridges differs anteriorly versus posteriorly (see above)? Contrary to this is that low amplitude two dimensional surface features are known to damp disturbances in laminar boundary layers (Bhatia et al. 2020) and thus decrease potential mixing. Could instead, coxal fluids be effectively injected as a puff of material into pre-existing circumcapitular reservoirs to encourage mixing? Muscles around the gland could aid this. Observations using dyes with fluorescence (Kwak et al. 2016) and interference laser-scanning confocal microscopy or synchrotron X-ray micro-radiography on mites might help considerably here in a follow-up study.

### Flow dynamic dependance

The deutosternal channel’s depth aspect ratio ($\lambda$) indicates the degree to which it functions as an open microchannel with simple pressure-driven fluid dynamics or whether filament-propagation transport is likely to be occurring. Elevated hydrostatic pressure can still affect microchannel flow by leading to local sagging of the interface between adjacent surface features (Hensel et al. 2016), like between the edges of the deutosternal groove at the micro-scale or the distance between adjacent transverse ridge asperities at the nano-scale. This sagging can induce a wetting state transition by contact of the fluid front with the bottom groove surface (i.e., between low height features; Erramilli and Genzer 2019) or by enforcing the propagation of the fluid front along the sidewalls (i.e., the inner flanks of the longitudinal secondary structure edges). All this is controlled by the height of the surface features. Determining the exact depth of the channel and denticles would require a separate SEM follow-up investigation than this preliminary review.

One possibility would be to assume that wider grooves have shallower depth, i.e., say $\lambda =\tfrac{1}{AGW}$ (Evert Lindquist *pers. comm.*). This would be equivalent to assuming that wide grooves always favour filament propagation fluid flow and narrow deutosternal grooves always behave in some sense like microchannel pipes. However instead, if one makes a few simple assumptions that

- the deutosternal ridge denticles cover the whole length of each ridge, and
- each denticle can be described as an equilateral triangle, and
- the denticles sit proud of the ventral chitinous surface with their tips just within the height of the channel (irrespective of whether they point anteriorly, posteriorly or ventrally),

then the minimum depth of the channel may be estimated from the data herein.

**Fig. 25:**
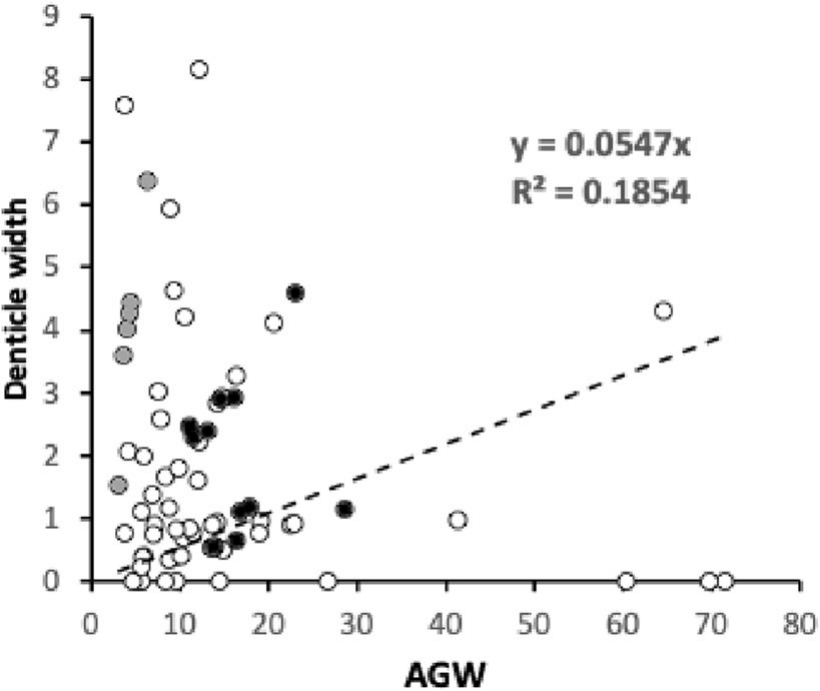
There is no universal deutosternal denticle size. All mites including *Uropoda abantica*, assuming equilateral shape of asperities. *Grey dots* Phytoseiids (top left). *Black dots* Macrochelids (middle and lower right)

Figure 25 shows that there is not a universal denticle width as calculated by $\tfrac{average\ groove\ width}{average\ number\ of\ denticles\ per\ ridge}$. There is again (see above) a hint of evidence that different designs may be present across mesostigmatids. Of course, this conclusion depends upon acarologists faithfully drawing the denticles from their specimens and not just adding suitable illustrative ‘wiggles’. A follow-up SEM study is needed to confirm in what way wider grooves have fatter denticles (as for instance denticle width is only mildly correlated with $BGW\ r=0.4341$ and with $BGL\ r=0.5012$). Such a study could also examine the geometry of the microstructures (Fu et al. 2017) and particularly if any triangular shape has straight or curved edges which have different effects on fluid drag (Hussain et al. 2017).

**Fig. 26:**
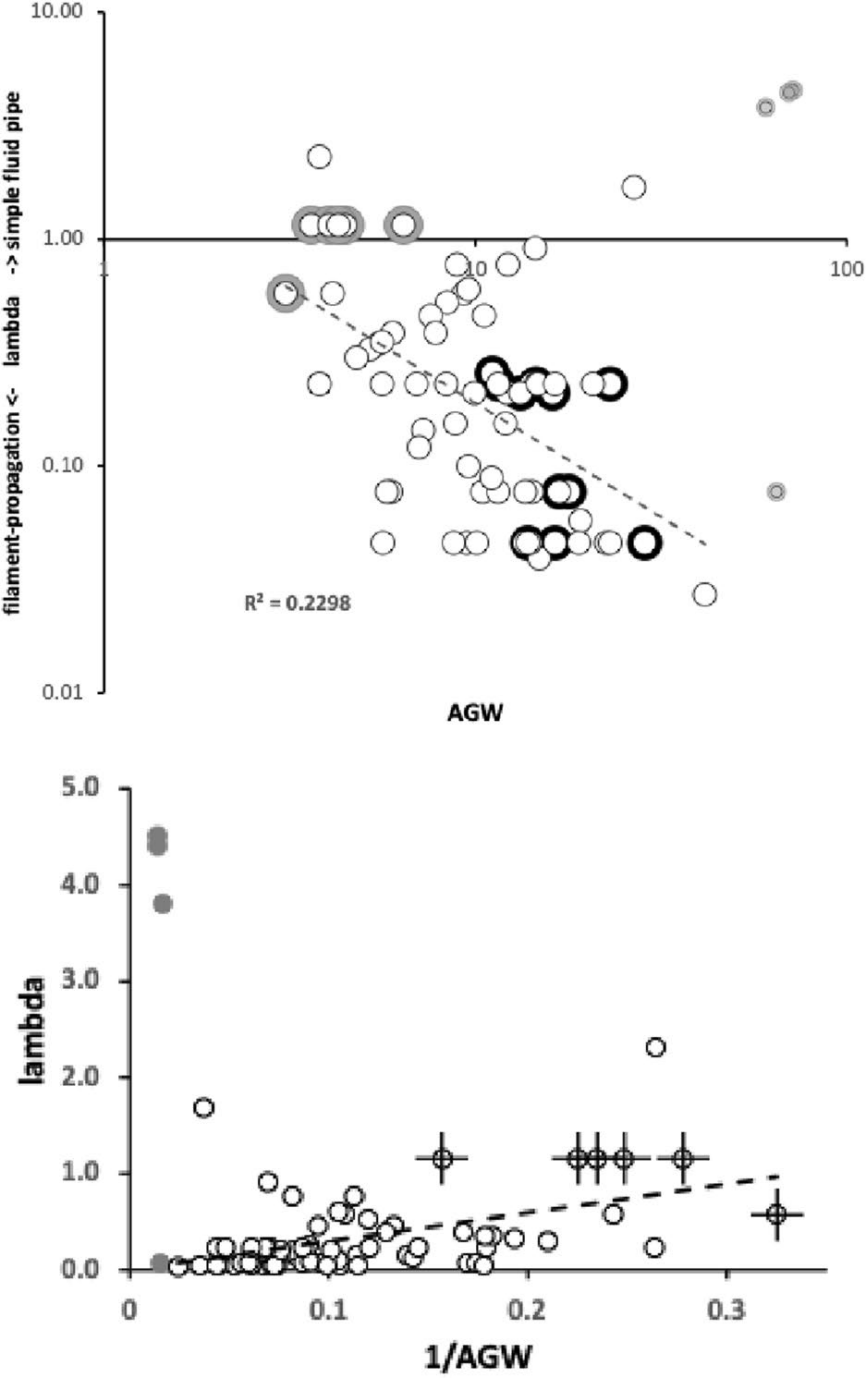
All mites including *Uropoda abantica*, *Upper* estimated microchannel dynamics versus groove width. *Small grey dots* Megisthanids. *Open large grey circles* Phytoseiids. *Open large black circles* Macrochelids. *Open circles* remaining species. *Lower* deutosternal groove depth aspect ratio ($\lambda$) prediction. *Small grey dots* Megisthanids. *Black crosses* Phytoseiids. *Open dots* remaining species

Looking at the species in this review, those with no ridge denticles were allocated a notional denticle width $=0.0547*AGW$ from an overall fitted regression equation. Then channel depth was estimated as $=1.15\,*\,denticle\ width$, i.e., with a $15\%$ excess justified by considering $1.15=\tfrac{1}{sin(60^{\circ })}$ (see third bullet point above). Ignoring the megisthanids on the *x*-axis on the right which represent zero denticle large body species where viscous creep of fluids may be occurring, Fig. 26 suggest that as mites get narrower deutosterna, the propensity for the deutosternum *microchannel* to act with tube-like simple fluid open pipe dynamics increases (see dashed regression line without megisthanids).

Also that phytoseiids and most of the rhodacarids show this type of channel design fluid dynamics. Excluding the zero denticle rhodacarids and laelapids has little affect upon this relationship. Larger mites like macrochelids show the opposite trend in deutosternal *microchannel* fluid dynamics design. As in general *AGW* approximately scales with *IL*, $\lambda$ also tends to rise in smaller mites and fall in large mites ($r=0.5027$), i.e., miniaturisation infers a change in fluid channel dynamics. Intriguingly, this echoes the simple assumption proposed by Evert Lindquist (*pers. comm.*) above despite $\tfrac{1}{AGW}$ being a poor predictor of $\hat{\lambda }$ overall $R^{2}=0.1642$. If megisthanids are excluded this prediction is much increased $r=0.7348$.

**Fig. 27:**
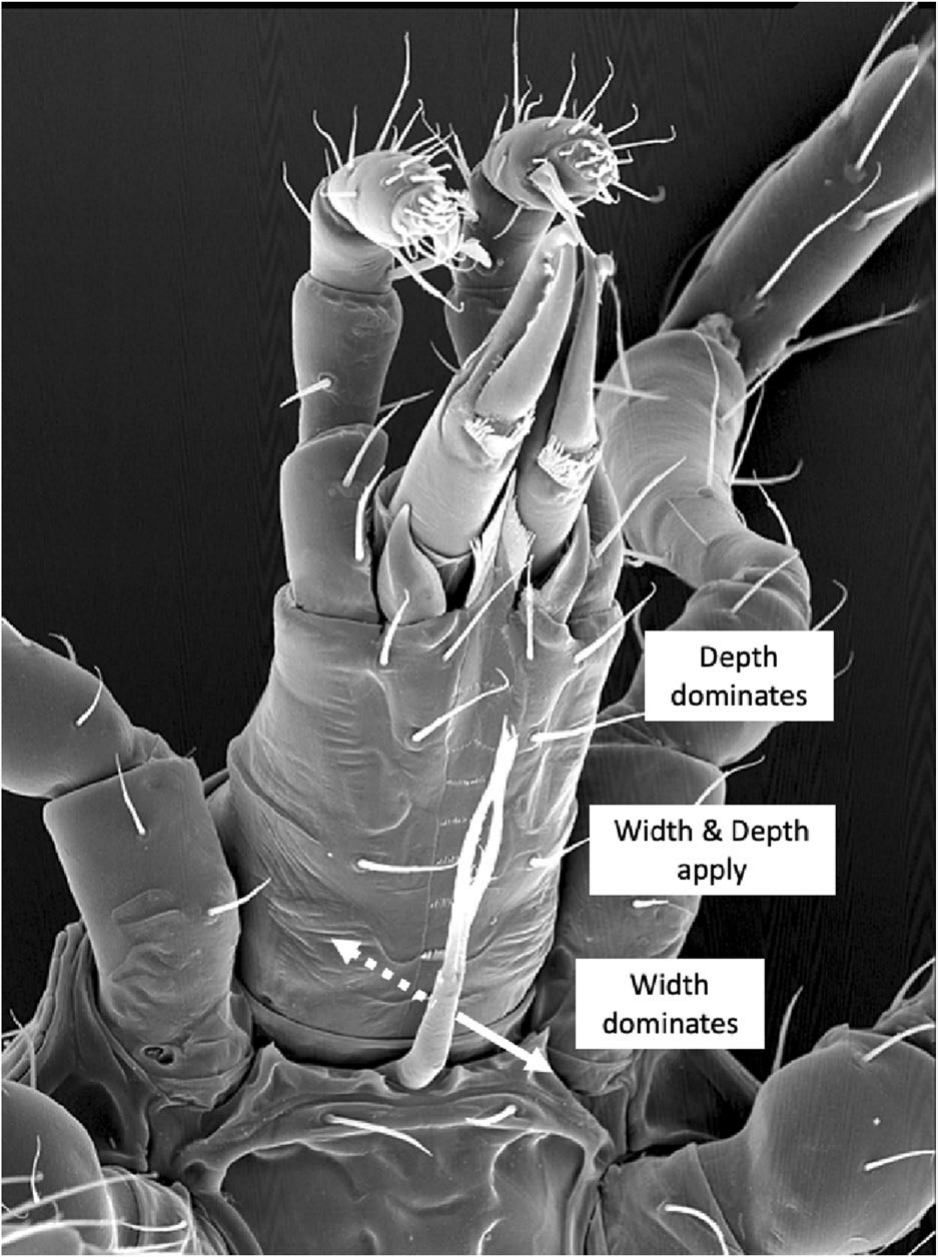
Different design criteria for flow dependency over different deutosternal regions according to if covered by: the tritosternum base (groove design $\Rightarrow$ pipe), the lacinae, or not at all (groove design $\Rightarrow$ microchannel, here outside of the fluid ‘ring’ described by Wernz 1972 and Wernz and Krantz 1976). The region covered by the lacinae can act like a pipe (tritosternum up against subcapitular venter, dashed arrow) or microchannel (tritosternum flexed away from the subcapitulum venter, solid arrow). The anterior groove region may comprise solely dye-sequestering nano-sized cuticular infoldings as their edges to function as nano-structures for edge crawling fluids. Corniculi again located as a cheliceral support mechanism rather than for impaling prey (as claimed in Evans et al. 1961). Annotated SEM of *Antennolaelaps* gnathosoma ex Photon Challenge: Last Chance (March 19, 2011) by Dave Walter at https://macromite.wordpress.com/page/4/ with permission

Figure 27 attempts to bring these ideas together. Different sections of the deutosternal groove area have subtly different fluid handling functions.

Sejids like Fig. 23 and *Epicroseius angeloides* (which is illustrated as ‘75’ in Hirschmann 1962) show similar posterior–anterior zoning. The relative scale of *PFRt* values are an indicative measure across species of fluid handling for that part of the subcapitulum covered by the tritosternal base, i.e., pipe width is dominated by *AGW*. Of course this needs adjusting by the relative length of the groove actually covered by the base for each species. Channel depth (and perhaps nano-scale infoldings) dominates the determination of fluid handling in central hypostomal regions beyond the tritosternal reach. The likely scale of this could be approximately judged say by

$$CFRt=\frac{(channel\ depth)^{4}}{(length\ of\ this\ section)},$$

where pipe flow dynamics is an estimate of the likely micro-channel flow. Of course, proper measurement is needed and validation via the micro-fabrication of biomimetics. Both channel depth and groove width may determine fluid flows in the regions covered by the lacinae. One way to imagine this, is that flow rates for this region are less than length adjusted *PFRt* but $>CFRt$. Perhaps using an average as the numerator in the flow calculation as an equivalent ‘pipe depth’ to give a measure denoted here as

$$MFRt=\frac{\left( \frac{AGW+channel\ depth}{2}\right) ^{4}}{(length\ of\ this\ section)}$$

would be a good place to start? Assuming as a first approximation that the relative subcapitular coverages do not differ between mite species (however, they most certainly does vary in practice) then $\tfrac{MRTt}{PFRt}$ is approximately $\propto \left( \tfrac{1}{2}\right) ^{4}$ as channel depth is $\ll AGW$. Furthermore, the relative $\tfrac{CFRt}{PFRt}\propto \lambda ^{4}$ across the reviewed species showing just how different a channel- versus a pipe-based design alters hydrodynamics (*figures not shown*).

The corollary of the deutosternal design result above is the conjecture that small mites (and phytoseiids) in general ought to have simpler tritosterna, whilst larger mites (including arctacarids) exhibiting a filament-propagation deutosternal channel design ought to have complicated tritosterna (with parasitids somewhere in between). A follow-up study could check this, with also a more accurate measurement of denticle sizes and channel depth (and relative coverage of the secondary structure). Large long conical denticles like the single central ones per Querleisten in *Asca elongata* (Abo-Shnaf et al. 2016), ought to function like puffer fish (Tian et al. 2022) or sailfish skin spines (Tian et al. 2021) in reducing fluid drag (as found for ‘cones’ in microchannels (Zhang et al. 2022). Ancillary subcapitular structures effectively reduce $\lambda$ favouring a filament propagation model of surface fluid transport even more. A wider tritosternal footprint might be expected to facilitate fluid movement over the ventral surface of the subcapitulum in these. Ameroseiids are possible examples here where deutosternal denticles are ‘outside’ of the deutosternum’s longitudinal edges (recall Evert Lindquist’s point above regarding the characteristic apomorphic single, wide, multi-denticulate, transverse ridge extending lateral of the deutosternum at the level of ca. the 6th row of deutosternal ridges). However, the tritosternum in *Ameroseius lidiae* although fimbriate at the edges of its base is not correspondingly dramatically wide (see Fig. 5; Khalili-Moghadam and Saboori 2014). How this all relates to the widespread feeding of many ameroseiids on fungal tissues (Mašán 2017), their strong apically furcated corniculi (Jerry Krantz *pers. comm.*) and unusual coarsely denticulated fixed cheliceral digits remains to be posed. Are fungal hyphae particularly water-rich? Do spores produce lots of fluid on consumption? Or, as Owen Seeman (*pers. comm.*) poses, could such mites be retrieving their food from watery habitats, like trying to eat spaghetti while avoiding the sauce? Mesostigmatids that feed on fungi or pollen usually have reduced number of teeth in the deutosternal gutter (Walter and Proctor 2013).

Any follow-up SEM study should also check that tritosterna in general over many mites are long enough for the whole structure to act in concert with the deutosternum to form an essentially tubular but open-sided pipe form for the large-scale transport of fluids. Confirmation of tritosterna matching the level of integration of the deutosternum over the whole subcapitulum is needed especially, as some photomicrographs of tritosterna (e.g., *Antennoseius heterochaetus*; Long et al. 2021) show them to be totally divorced from and well posterior of the circumcapitular groove. Mite preservation and preparation techniques will impact this judgement as artefacts can be generated due to pressure being applied to microscope sides. Is the lack of a deutosternal groove extending anteriorly into the hypostome or posteriorly to the circumcapitular groove as in *Leptogamasus chelatus* (Witaliński 2022) complemented by tritosternal features or compensated for? There is certainly much for a keen acarologist to do.

Finally, the denticles in most mesostigmatid species appear to point anteriorly but this direction of overhang also needs detailed confirmation with scanning electron microscopy.

## Discussion

In judging the suitability of a mesostigmatid to be a BCA, of course other biological parameters must come into play, such as capacity to be reared *en masse*, preference for the proposed climate where it might be used, etc., than just possessing an ideal morphology. However, small size matters in mesostigmatids as does exactly how big a fluid handling challenge is to be faced by the predator. These mites inhabit a ‘Goldilocks zone’ where focusing on fluids and the quantitation of volumes is a sensible first step in understanding mite oral hydrodynamics (as for instance desiccated mites withdraw their chelicerae back into their idiosoma as their effective inner space declines). Starting with a simple model then elaborating it in further finer and finer detail is a classic epistemological approach.

Some assumptions made herein could be questioned. For instance while dynamic viscosity (i.e., ‘stickiness’) should be generally similar for different prey fluids as all need extra-corporeal digestion, the pressure difference $\varDelta p$ may vary with perhaps bigger mites being able to generate bigger pharyngeal pumping differences and thus compensate for their *PFRt* in maintaining deutosternal flow rates *Q*. Note capillary pressure ($\varDelta {p}_{c}$) is expected to be fairly uniform across species. It is acknowledged that prey fluids themselves may be non-Newtonian fluids like blood, colloidal suspensions, polymer solutions, etc., so this review’s conclusions mainly pertain to water and dilute solutions being transported. Furthermore, this exposition also does not allow for any compressibility (i.e., ‘springiness’) in fluids being moved around. Particular topologies of stable flows of combined coxal fluid with prey material through ‘pipes’ could be posed, e.g., stratified, wavy stratified, smooth annular, wavy annular, throat annular, segmented (plug/slug), deformed plug, dispersed flow, etc. One could also criticise that pipe flow rates in assuming a cylindrical pipe does not allow for say very flattened tritosterna. Also there is the possibility that some mites might specialise in recycling fluids through the deutosternal/tritosternal assembly many times; however, for at least pergamasids Bowman (2014) does not describe multiple sequential coxal droplets being formed. Assaying coxal gland alkaline phosphatase activity over time in different species during feeding may be helpful here as it is indicative of fluid transporting epithelia.

Mesostigmatids clearly have the capability to handle prey fluids at varying capacities. Nature can inspire many smart materials for directional liquid transport (Cui et al. 2017). Self-motion (i.e., with no direct pumping) can often feature (Dai et al. 2020). This pilot functional review sought to discern what aspects of mesostigmatid subcapitular design might be related to the deutosternum acting as: a complete but open-sided pipe, versus a droplet transporting Cassie (and Wenzel) state microchannel mechanism, versus a fluid filament-propagation microchannel mechanism, and whether mixing might occur. It deploys laminar flow arguments to point to features of interest in future BCAs (detailed understanding of turbulent flow at this micro-scale is a different matter).

**Fig. 28:**
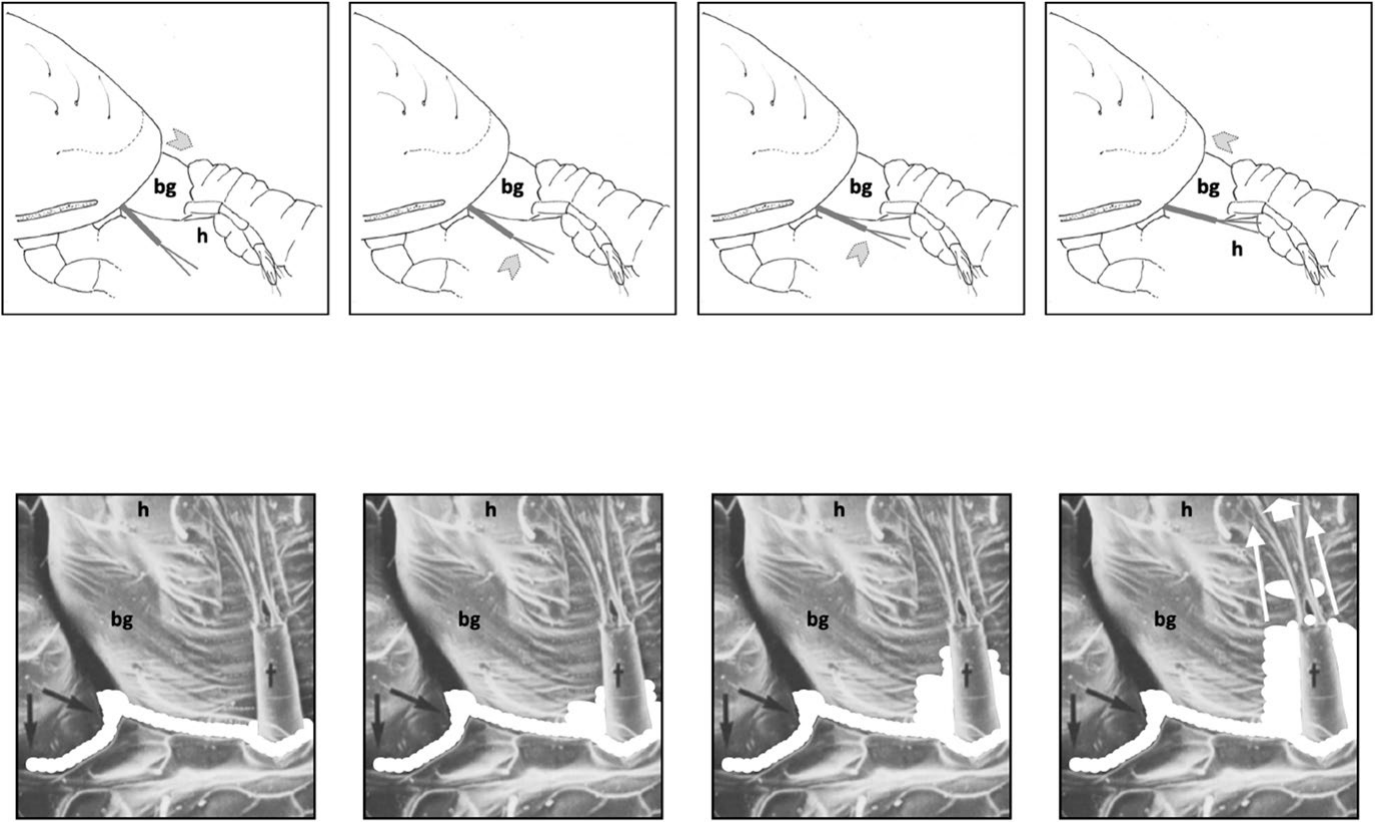
Summary of deutosternal recycling mechanism as prey fluids are progressively imbibed, based upon Wernz (1972) and Wernz and Krantz (1976). *Upper*, *left to right* relaxation of muscles and sternal adjustments means tritosternum is held away from *basis gnathosomatica* (*bg*) on an extended gnathosoma as chelicerae actively masticate prey (Wernz 1972) and the mite imbibes resultant extra-corporeal digestive products (deutosternal ‘switch’ off) $\rightarrow$ tritosternal muscles contract progressively raising the tritosternum towards deutosternal groove $\rightarrow$ until lacinae rest at first basally against hypostome (*h*) on now a retracted telescoped gnathosoma with paused extended chelicerae, lacinae finally touching the hypostomal fluid ‘ring’ and circumcapitular liquids majorly flow forward rapidly (deutosternal switch ‘on’) and are re-imbibed. Lateral view drawn and amended from colour photograph of a feeding soil mesostigmatid ($\copyright$ Andy Murray/chaosofdelight.org with permission). Leg 1 omitted for clarity. *Lower*, *left to right* matching location of ventral liquids shown in white. From being prey overspill plus coxal fluids beginning to debouch from glands under coxa I (black arrows) in circumcapitular groove and around tritosternal base only $\rightarrow$ progressively forming (Cassie state) droplets as meal is concentrated internally, then advancing and widening (Wenzel state) ‘droplets’ pinned on *basis gnathosomatica* $\rightarrow$ until microchannel imbibition starts with viscous flow edge crawling filaments (white arrows) along in-folded (nano)channel edges (Wernz and Krantz 1976) and the lacinae reaching into the hypostome $\rightarrow$ to form a whole micro-‘pipe’ of droplets across longitudinally ridged groove. Note only one semi-spherical bulge shown for illustration covering an inter-ridge cell through to the hypostome tip (white block arrow). Based upon Fig. 4 of Bowman (1984) $\copyright$ Ellis Horwood Limited with permission

A scheme for how these modalities may relate to each other is shown in Fig. 28. It is consilient with the scheme outlined in Figs. 4, 5, 6 and 7 in Wernz and Krantz (1976)—extension of both chelicerae making space in the idiosoma internally for the bolus of buffered fluid to be imbibed on gnathosomal retraction/flexure pushing fluid films out and about being pinned and de-pinned on various ridges. In other words, by the “..compression of the postcapitular channel and a momentary infolding of the tritosternal base, causing fluids to be forced into the...” deutosternal groove (Wernz and Krantz 1976). This, with the key relative length of the tritosternum, is then the required ‘switch’ to control the hydrodynamics. Oscillations of the tritosterna would advance or relax and deform the fluid bulges appropriately facilitating pharyngeal fluid pumping.

Polyaspids and trachytids have particularly wide tritosternal bases, and the laelapid *Neolaelaps spinosa* an overall wide tritosternum with broad lamellate lacinae (Wernz 1972), does this facilitate a larger initial Cassie state droplet? Does tritosternal muscle contraction cause the base to squeeze such droplets forward? Gnathosomal retraction and folding of the cuticle at the circumcapitular groove would ensure tritosternal lacinae can reach completely anteriorly (Wernz 1972) to the tip of the hypostome if required. Note that Flechtmann and McMurtry (1992a) describes the tritosternum as being inserted “in a soft, extensible cuticle” and maintains that in the phytoseiid *Euseius stipulatus* the tritosternum moves “back and forward with the subcapitulum as a single unit”. Meaning that it may not independently glide along the deutosternal groove as Wernz (1972) describes. This would be reasonable if the groove is acting as a continuous low volume microchannel ‘dribble’ not requiring an on/off switch. Flechtmann and McMurtry (1992a) also did not observe any grooming behaviour between phytoseiid feeding episodes which would also be consilient with such a deep rectangular fluid gutter. Indeed prey fluids may never overspill into the phytoseiid posterior gnathosomal area given the description of the klinorhynchid attack (and discard) pose of phytoseiids (especially for the pollen feeding species *E. stipulatus* and *Amblyseius similoides*) where the gnathosoma is bent almost at a right angle to the gnathosoma (Flechtmann and McMurtry 1992a) and material imbibed. This action could vacate parts of the internal volume of the propodosoma to allow easy continual fluid transport into the gut while fluids leak out and pool around the prey wound. The reorientation of the gnathosoma (and the chelicerae) afterwards being the pressure pump to gently force fluid out of the idiosoma via the coxal glands and so continue a narrow threaded low flow anteriorly up to the hypostome. Gleaning material off of pollen would similarly produce only small volumes for phytoseiids to handle.

In larger mites, dyeing experiments on tritosternectomised mites (Wernz 1972) confirmed halting of drops at the tip of the tritosternal base before microchannel imbibition. These more sclerotised mites may not need the longitudinal edges to deutosternal grooves as strengthening. There also is no need to pose any regular prey overspill channeling of liquids down the subcapitular sides in the parasitid and macrochelid species (stippled arrows in Fig. 5 of Wernz and Krantz 1976). Such would be impossible to switch on or off or to control their backwards or forwards direction in the absence of a morphological mechanism. Wernz (1972) only describes dye fluids doing this in abnormally dry specimens. Unusual overflow if it arises could be simply guided via the *whole* central laterally augmented deutosternum/tritosternum assembly grossly acting like an overall ‘wick’ intermittently as necessary, and the circumcapitular groove (and associated leg coxal structures) being the buffer for the initial flood before rapid imbibition diminishes it.

Testable corollaries of this scheme by which consuming prey digestive liquids is interspersed with re-imbibition of coxally derived solutes is that, free-living predatory mesostigmatid mites should hunt with both chelicerae relatively extended and their gnathosoma relatively retracted. Further that, during active mastication of live prey tissue alternatively by each chelicera the gnathosoma should be relatively extended (and may be airorhynchid in arrangement; Bowman 2020). Pharyngeal fluctuations should be seen throughout both phases. Wernz (1972) already describes a downward ventrally curving flexure to a klinorhynchid arrangement to facilitate removal of circumcapitular fluids (even in tritosternumectomised mites). One suspects that uropodoids with their usual klinorhynchid architecture may follow the phytoseiid style of fluid handling i.e., little risk of fluid overflow, integrated tritosterna on their subcapitulum, etc. If phytoseiids do not need to handle large amounts of prey fluid then their coxal glands may be similarly small and/or relatively inactive. A comparative cytochemical micro-anatomical study would be useful.

What might the initial classification of deuterosternal forms (Bourdeau-Gorirossi 1989) be trying to say functionally? Is there any evidence of a hierarchy of surface structures optimised for specific wetting conditions (Teisala and Butt 2019)? Phytoseiids may use alternative morphologies to prevent the risk of fluid overflows, such as the flange-like expansion on the abaxial face of the cheliceral fixed digit (aka ‘lobes’ Flechtmann and McMurtry 1992b) or cheliceral shapes (Flechtmann and McMurtry 1992a) themselves being sufficient to steer even the little overspill from dry prey being steered mouth-wards. They may not need macrochelid-like structures. Phytoseiid species with ‘spoon-like’ lobe structures on their cheliceral paraxial face also have a relatively wide deutosternal groove (Flechtmann and McMurtry 1992b). Could they deal with more watery volumes than the usually relatively narrow groove species?

For sure, potential slot-like architectures, also illustrated for *Trachytes pauperior* in Hirschmann (1962), need more investigation. Are clear longitudinal edges to deutosternal grooves associated with smallness? To what extent might they be nano-tubes or nano-channels? More investigation of uropodoid mites is needed. For instance, the form of the gnathosomal insertion and its relationship with coxae I in *Protodinychus* is unique among the gamasines in that each coxa has a distinct acetabulum with a paraxial connection to the camerostome (= cavity containing just the gnathosoma) through tough unsclerotised cuticle (Athias-Binche and Evans 1981). This partial separation is intermediate in form between the situation in holothyrids (in which these acetabula are entirely separated from the camerostome) and that in other mesostigmatids where coxae I lack distinct acetabula and are situated with the gnathosoma and tritosternum within a gnathopodal cavity. *Protodinychus puntatus* has the primitive characteristic structure allowing direct contact between the products of the coxal glands, opening ventrally on coxae I and the tritosternum. Moreover the deutosternal groove (of at least the deutonymph) has rows of large paired almost flabellate denticles themselves micro-denticulate (see Fig. 7 in Athias-Binche and Evans 1981). How might these work? Is the micro-denticulation working like the phytoseiid scale sub-denticles?

Other *Trachytes* spp. are illustrated with multiple deutosternal grooves (Mašán 2003). How do these work? *Trematurella elegans* shows a very long ridge-less deep walled gutter sunk in along the whole subcapitulum which appears to have within it a central slit flanked by longitudinal ridges (Błoszyk et al. 2018). Could this function like the anisotropic wettability of butterfly wings where longitudinal microstructures at multiple scales control fluid flow (Erramilli and Genzer 2019)? The elongate gnathosoma venter in *Oplitis turcica* has a central suture-like groove with various spicules around it (Bal and Özkan 2006). Could this work similarly? The deutosternum is very narrow with possible central spikes in *Nenteria bastanii* (Kazemi and Abolghasemi 2016), how might it function? Other uropodines appear to have feeble or no longitudinal edges to their deutosternum e.g., *Phymatodiscus insolitus* and *Rotundabaloghia (Circobaloghia) singaporica* (Kontschán and Géza 2016). How does this function hydrodynamically?

What is the situation in other gamasines? Larger ‘bruiser’ mite designs adapted for tearing into and overcoming active prey that can fight back (like large fly larvae) might not be worth investigating as BCAs. For sure more eviphidids may be worth examining for BCA potential (as they have their position of the most anterior row of deuterosternal teeth slightly in advance of seta $h_{3}$ too (Wernz 1972). A weakness of this review is that hypoaspids like *Stratiolaelaps miles* and *Stratiolaelaps scimitus* which are used commercially across the world for sciarid control (Diana Rueda-Ramírez and Eric Palevsky *pers. comm.*) were not included as a ‘positive control’. A smart acarologist could apply these review results to all the current commercially available mesostigmatids, plus field test biologically some deutosternal morphology inferred ‘functionally inappropriate’ mites from say a large follow-up study of parasitids (Hrúzová and Fend’a 2018) in order to validate or refute the results of this study and build a morphological statistical predictor. That predictor to consider (with appropriate functions and combinations like $\hat{tep^{*}}$): *IL* (body size for prey access), *CLI* (reach for grabbing prey), *MDL* (gape of bite), *VR* (velocity ratio, being indicative of the speed of chelal closure), *F*2 (chelal crunch force), plus those elements *AGW* (average groove width without lateral extensions), *GL* (groove length), *BGW* (*basis gnathosomatica* width), and *CHI* (cheliceral height) making up *PFRt*, circumcapitular volume (*A*), the volume displaced by a single chelicera on protrusion/retraction (*B*), and deuterosternal volume (*DV*). Extra terms like width of the distal cheliceral segment (*WDS* if available) and groove measures such as $\lambda$, denticle width, number of ridges, etc., could also be included. Taxonomists could routinely use this then to score mites for BCA potential in advance, or at least use their measurements to suggest how the mite’s oral hydrodynamics might work. For future investigations it would be useful for such taxonomists to also draw in detail the subcapitulum and tritosternum of specimens as accurately as possible even when of limited systematic distinction.

In the meantime, what is the situation with early primitive trigynaspid groups like cercomegistines where irregular rows of denticles even occur on the dorsum of the gnathotectum (Lindquist and Moraza 1993)? How does the tetartosternum (= presternal shields) interact with a duplex tritosternum and the highly developed cheliceral excrescences in the fluid feeding fedrizziids (Seeman 2007)? How do such mites push the excrescences out of the gnathosoma without them going all over the place (Owen Seeman *pers. comm.*)? Do micro-channels within them sequester fluids by capillarity to collect internal juices from prey carcasses and facilitate scavenging (Seeman 2000)? How then is the fluid released from these ‘mops’? Does a structure somewhere squeeze them dry? How do the central subcapitular structures in opilioacarids (‘sternapophyses’; Vázquez and Klompen 2002; Moraza et al. 2021) deal with fluids? What can be learnt from the use of slender finger-like tritosterna in whip spiders (Amblypygi; Dunlop and Alberti 2008)? There is much for acarologists to investigate building upon the comparisons in Wernz and Krantz (1976). Whatever is concluded any functional scheme must be able to explain: grooves with no Querleisten (e.g., *Spelaeorhynchus* sp. in Wernz 1972), as well as strange and sparse subcapitular designs like in the epicriid *Epicrius mollis* and the parantellulid *Diplopodophilus antennophoroides* illustrated in Hirschmann (1962), and even the peculiar ‘bead-let’ groove shape in *Epicriopsis berlesei* (Karg 1965). Any scheme must make sense with hypostomal variation over gender and stage and make sense with known food differences (Seeman 2000, 2012, 2022).

As Owen Seeman (*pers. comm.*) has pointed out, one possible critical test of the results of this review would be by deliberately looking at deutosternal function during ontogeny. Larvae are often non-feeding. Some groups, such as Antennophorina, go through a stunning metamorphosis in their chelicerae. This is most apparent in the Fedrizzioidea (compare Seeman 2000 to Seeman 2007), Parantennuloidea (see Womersley 1958 and Seeman 2012 on *Promegistus*), and Paramegistoidea (compare *Neomegistus* and *Derrickia*, which is thought to be the immature stage of a paramegistid). The Antennophoroidea, Megisthanoidea and Celaenopsoidea are impressive arthropods. Immatures of these mites, where studied, are free-living predators with rudimentary cheliceral excrescences. Then, on their hosts, they develop extraordinary excrescences and sometimes highly modified chelicerae. Those with the latter are probably tending to kleptoparasitism and parasitism; those that do not modify their chelicerae might have a multi-functional ‘swiss-army knife’ approach—excrescences for scavenging, chelicerae for picking up prey. Whatever the case, immatures are behaving like typical Mesostigmata while adults are doing different things—does the deutosternum change too? Differences between sexes is probably less common but perhaps interesting too. A recent description was of a mite with females having very different chelicerae to males (the female’s moveable digit looks like a tree-saw); the male’s like a typical diplogyniid-form with a scoop-like ‘spermatodactyl’—see *Terrogynium weatherwaxae* (Seeman 2022). Does one want all stages of a mesostigmatid to be designed as a BCA or just particular sized ones?

Critical comparison of actual ridge heights to shark skin features (Pu et al. 2016) is needed. At 151$\ \upmu$m (Bhatia et al. 2021), a sharkskin denticle (at the top) is around the same length as the overall mean deutosternal groove length ($GL=94\ \upmu$m) in this review. The latter however is half to a third the size of denticles on thresher shark tails (Popp et al. 2020). At $14\ \upmu$m, the overall deutosternal width (*AGW*) in this study is much narrower than the ($51\ \upmu$m) spacing (aka the ‘gully’) between adjacent ridges in shark denticles (Bhatia et al. 2021). But all face the same challenge of fluid flow past them. If the exterior lateral extensions shown by mites are included, this only increases the overall average $AGW+$ extensions to $21\ \upmu$m. However, the $AGW+$ extensions figure for arctacarids $=98\ \upmu$m and that for megisthanids $=67\ \upmu$m, do compare well to the shark gully design (and ridge spacing on thresher shark tails Popp et al. 2020). Other mite families have much narrower flow systems. Do smaller sharks have smaller denticles? Can existing learnings be read-across?

Interestingly, the sharkskin denticle width in Bhatia et al. (2021) at $125\ \upmu$m better compares to the overall mesostigmatid *basis gnathosomatica* width at $154\ \upmu$m. The latter also approximates the $145{-}233\ \upmu$m denticle width of thresher shark tails (Popp et al. 2020). The mite gnathosomal venter overall thus is approximating the upper planar form of a *single* gullied shark denticle (the middle deutosternal groove just marking the centre of the gully). This supports the conclusion of a similar gross function with respect to overall fluid flow over them. Regarding depth, the gully between the mid- and side-ridges of shark denticles suggest a value of around $11\ \upmu$m to $21\ \upmu$m (Bhatia et al. 2021), or a depth aspect ratio $\lambda \approx 0.2{-}0.4$ which compares favourably with deutosternal values for mites tending towards the phytoseiid design (Fig. 26 Upper). The ridge spacing and height in thresher shark tails is 23–45 and $1.1{-}5.9\ \upmu$m, respectively (Popp et al. 2020), indicating a depth aspect of $\approx 0.02{-}0.26$ again strengthening commonality of function but this time with the macrochelid type of mite design. Does the effective protrusion height (Dean and Bhushan 2010) of longitudinal flows match? Could investigating flow speed past different shark skin designs inform understanding of deutosternal relative flow speeds? What is the likely protrusion height of any cross flows? More characterisation is needed. Both denticles and their riblets are important in sharks (Lloyd et al. 2021). However, at a gross level, if one considers the whole *basis gnathosomatica* to be equivalent to one shark denticle width and *GL* to its length centrally through its ridge-defined gully, then perhaps the ranking of the $\frac{BGW}{GL}$ values for specially collected megacephalic mesostigmatids might be compared to the ranking of the denticle width/length ratio of other shark species in Table 3 of Popp et al. (2020) in a follow-up study? Perhaps then the relative swimming speeds of the sharks species might suggest a likely ranking of prey fluid handling speeds washing over the whole mesostigmatid primary deutosternum–tritosternum assembly structure for at least mega-cephalic mesostigmatids?

The fine detail of arthropod microstructures can yield surprising behaviours (Boevé et al. 2004; Hischen et al. 2017; Watson et al. 2017a, b). Patterns of hydrophobicity and hydrophilicity of surfaces can collect (Kim et al. 2019) and directionally transport water (Zhu et al. 2021). Indeed morphological adaptations (including C-shaped channels) to channel liquids in mouthparts are known in other arthropods (Lehnert et al. 2022), which are also self-cleaning (Kay 2022). Self cleaning relies upon lack of adhesion, not mechanical actions like gnathosomal movements causing the raking actions of tritosternal lacinae described by Wernz (1972).

Various studies have looked at mesostigmatids morphometrically (Buryn and Brandl 1992; Flechtmann and McMurtry 1992a, b; Adar et al. 2012; Liu et al. 2017 etc.) and conclusions drawn as to how structures might work. However, empirical models need a quantitative theoretical basis to be functionally believable (Bowman 2020). Any fluid handling models must also seamlessly apply to mesostigmatids in marine algae and beach wracks within periodically submerged intertidal zones (Gwiazdowicz and Teodorowicz 2017). Any gnathosomal shape changes must have a demonstrable biological impact to be relevant. This might be by experimental validation or biomimetic constructions but at least requires checking their numerical consilience with already gained observations (as in this pilot feasibility study). Finally, a unified mechanism needs proposing to explain the observation by Wernz (1972) of an “...additional smaller droplet occasionally formed anterior to hypostomal setae..” $h_{3}$.

**Fig. 29:**
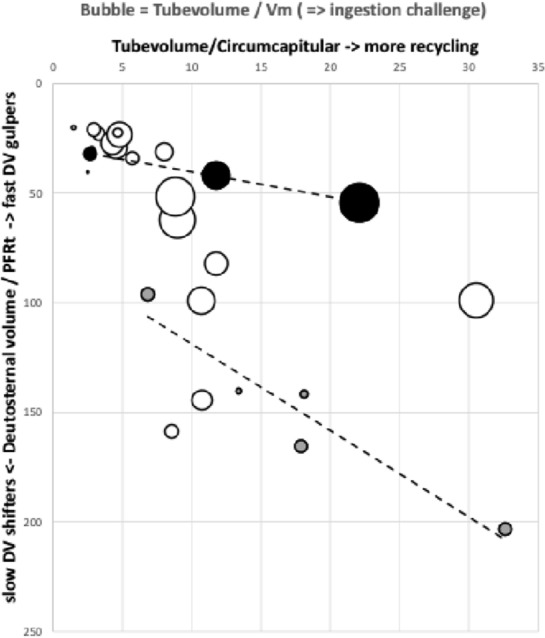
Summary based upon deutosternal groove acting as a pipe (not including *Uropoda abantica*), *Grey dots* Phytoseiids. *Black dots* Macrochelids and Parasitids. Bubble size is the relative prey load, i.e., the volume available for fluids from a single cheliceral retraction/protrusion compared to predator volume. The *y*-axis is the time to shift a deutosternal volume ‘gulp’ through the pipe. The *x*-axis indicates the number of times fluid passes through the circumcapitular groove from coxal secretion out of the haemocoel from the gut after ingestion. Low *x* values indicate a hard to saturate circumcapitular buffer

What does this mean regarding phytoseiids? They are a distinct functional group in the gamasines (Fig. 29).

**Fig. 30:**
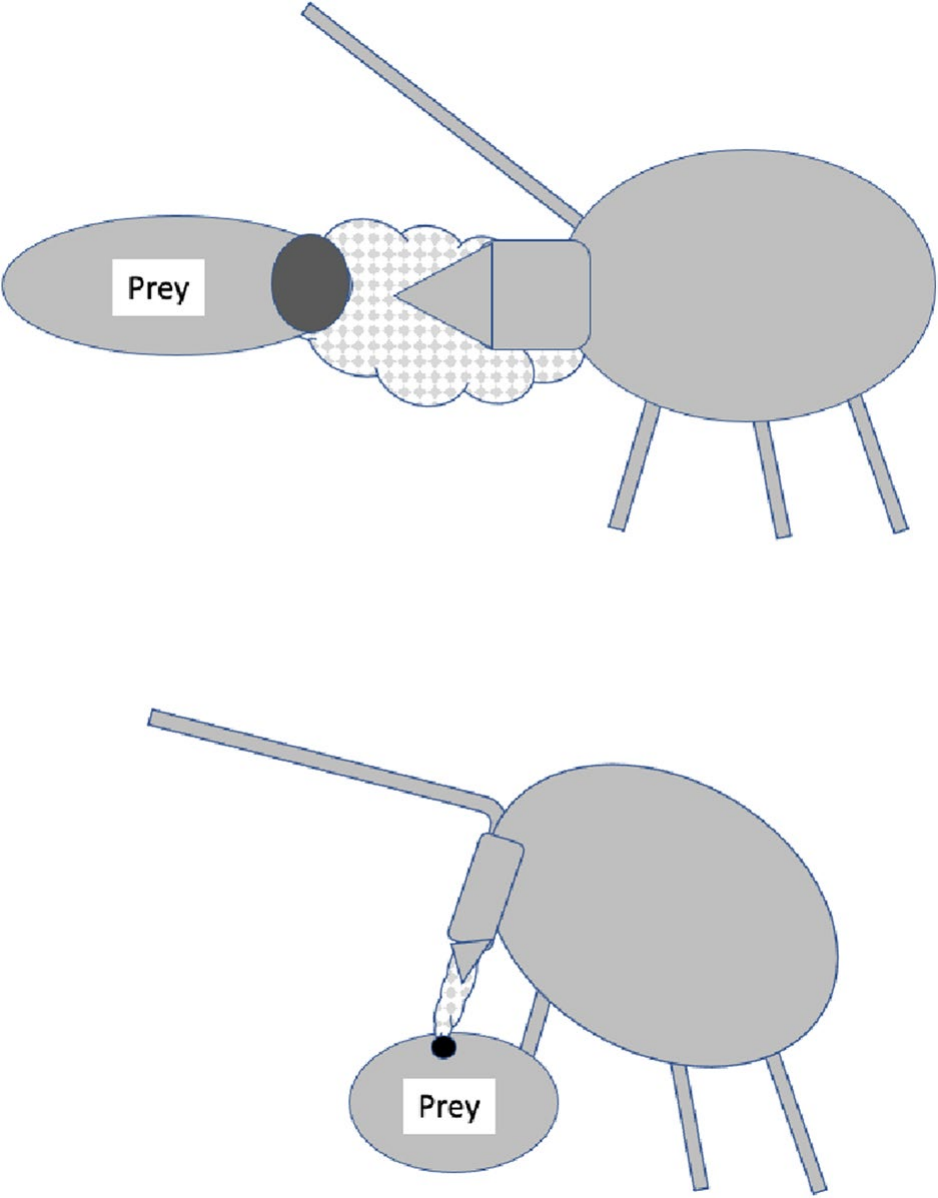
Large airorhynchid attack style mega-cephalic mesostigmatids (*Upper*) produce a large wound and crushingly feed on or slice-up high watery fluid prey ‘end-on’ requiring major fluid handling. Microcephalic klinorhynchid attack style (by design or by behaviour—see p. 158 in Flechtmann and McMurtry 1992a) predators (*Lower*) rely upon effectively tube-like straw drinking of liquidised prey via a small aperture cut wound (even in a leg). Here the relatively smaller dry prey require little major fluid handling. Both palps, chelicerae and legs on one side of the idiosoma omitted. Gap between the cheliceral/hypostomal tip to prey exaggerated simply for clarity of exposition. Prey could be eggs, larvae, tetranychids or pollen. Prey overspill can be obviated by very rapid imbibition into the gut. The prediction is that more potential BCA agents may belong to the lower feeding group than the upper

They appear to shift fluids comparatively slowly having a low ingestion challenge with most species showing little need for excessive recycling (so even on repeated feeding therefore). This is consilient with observations in Flechtmann et al. (1994). In this review only a few other mites had very similar fluid handling competencies, e.g., *Asca nelsoni* and *Ololaelaps formidabilis* for sure (and possibly *Philippinozercon makilingensis* and *Proarctacarus canadensis*). The contrast is linked to the phytoseiid style of prey attack (Fig. 30).

Obviously examining other candidate phytoseiids and rhodacarids as BCAs could be useful experimentally. Table 6 illustrates the results for a variety of candidate BCA phytoseiid mites extracted from recent literature with trophic parameters. It is not exhaustive of all possible free-living mesostigmatids. Flechtmann and McMurtry (1992b) report cheliceral lobes in *Neocypholaelaps* (Ameroseiidae), *Proctolaelaps* spp. (tribe Melicharini), *Antennoseius* (Ascidae) and *Lasioseius youcefi* (tribe Blattisociini). If this feature is a conduit relevant to phytoseiid success as a BCA, then predatory species designed like these too could be investigated further. Given that some flower mites (Paciorek et al. 1995; Velázquez and Ornelas 2010) which feed on pollen belong to the Melicharini tribe (Tschapka and Cunningham 2004), these could be worth examining too.

The fluid content of plant pests (like tetranychids, eriophyids, tenuipalpids, scale insects, thrips, etc.) needs investigating compared to say fly larvae. However, based upon the arguments outlined above, this pilot study suggests searching for a mite species designed to handle relatively dry prey as a new BCA might be usefully investigated, e.g., out of the species studied herein: ascids like *A. nelsoni*, *A. wisniewski*, or blattisocids like *Lasioseius orangrimbae*. These are gamasines with usually few denticles per ridge that are neither too big, yet have a decent reach (including *CLI*/*IL*) and hopefully are active searchers/pursuers of prey with the right reproductive efficiencies (though note that some lasioseiids are mycophages; Walter and Lindquist 1989). Slightly larger laelapids like *Ololaelaps tasmanicus* and *Gaeolaelaps iranicus* could also be tested as BCAs as one wants to maximise the BCA body volume to prey volume ratio if possible to facilitate repeat feeding (as in the commercial use of *Androlaelaps casalis* against juvenile *Dermanyssus* sp.). Later perhaps other *Gaeolaelaps* spp. (Nemati et al. 2018; Khalili-Moghadam et al. 2018) especially if elongate (like the small-pore nematophagous guild of soil mesostigmatids, e.g., *Protogamasellopsis zaheri*) might be examined that were not studied herein. Of note is that *Gaeolaelaps aculeifer* and *Gaeolaelaps gillespiei* are already used for thrips and dipteran control commercially (Diana Rueda-Ramírez and Eric Palevsky *pers. comm.*). Medium-sized small general predators like megisthanids, macrochelids, parasitids, arctacarids, and eviphidids are inferred not to be so highly effective against tetranychids rather being more adapted to handle larger more fluid rich prey. Consilient with this is the European commercial use of: *Macrocheles* species including *M. robustulus* against dipteran, thrips, and lepidopteran pests (Diana Rueda-Ramírez and Eric Palevsky *pers. comm.*; Azevedo et al. 2018).; and *Pergamasus quisquiliarum* against symphylans (Groth 1997).

Given the importance of prey liquefaction in phytoseiid feeding (Flechtmann and McMurtry 1992a), a comparative protease study between candidate BCA mesostigmatids could be useful too. An entry point for enzymic work on soil-inhabiting predatory mites is Bowman (2019). Recent work in parasitic dermanyssids in comparison can be found in Murata et al. (2021) and in Ariizumi et al. (2022).

The key point in this review is that the mesostigmatid deutosternum/tritosternum assembly appears to be designed (as a fluid dynamic ‘micromachine’; Teng et al. 2012) to fulfil several functions with a reversible mechanism for how it switches between these. Flows can be tuned and may be minor, slow and possibly continuous via a film of edge crawling fingers depending upon the relative arrangement of gnathosomal parts. Flows may need to be fast and of high volume intermittently in what amount to pipes. Special channels may effectively act like micro-pipes with central threads. Movement of fluids may thus entail encouraging complete or partial or no wetting. Conversely transport of fluids may be via the movement of discrete droplets or bulging maxima of a wave along semi-wetting paths. Transport can be pumped or by capillarity. For sure at this scale, a ridge shape that matches the likely meniscus shape (concave with respect to direction of movement e.g. anteriorly in *Pergamasus runciger*; Hirschmann 1962) will facilitate the capillary fluid transport in that direction from gnathosomal compartment to compartment essentially irrespective of physical slope. The famous 1955 artwork by M. C. Escher called ‘Convex and concave’ in the National Gallery of Canada (https://www.gallery.ca/collection/artwork/convex-and-concave) illustrates that many different transports may simultaneously occur in certain topologies. Mite surfaces can have this complexity. Accordingly, during mesostigmatid evolution to particular niches many simultaneous hydrodynamic optimisations and trade-offs will have occurred.

Mesostigmatid mites can show microchannel designs and micro-pipe designs with ridges extending laterally beyond the deutosternal groove limits in the region of the tritosternal base (Fig. 6). This may facilitate holding a large discrete fluid droplet here when the tritosternum is lowered and the lacinae are away from the groove. Evert Lindquist (*pers. comm.*) points out that determining the edge of the deutosternum using its longitudinal edges is perhaps OK for macrochelids generally, but in some gamasines a couple of the basal transverse rows of deutosternal denticles are posterior to those edges. Could these facilitate holding such a droplet here? Indeed could such a tritosternaly carried droplet be compressed against and make surface contact with the deutosternal channel changing it from a stable Cassie state to a Wenzel state and engendering the commencement of deutosternal pipe flow (i.e., control and facilitate intermittent high volume re-imbibition through the channel and associated lateral extensions when fluid excretion from the idiosoma is high)? Exactly how might the different deutosternal structures along its length functionally interact with each other? Different *Proctolaelaps* species (Melicharidae) have transverse ridges some straight, some curved, some with few or many large or small denticles (and some without denticles) and a groove with and without longitudinal edges as one moves anteriorly from the circumcapitular groove even within a species (Abo-Shnaf and de Moraes 2016). Are the prey, their volume and fluid handling that much different between these species to engender such within-genus evolution?

A variety of other questions arise. Could tritosternal oscillation be another driver of a periodic fluid wave of semi-sessile droplets? The tritosternum does have sophisticated musculature (Wernz 1972). Exactly what is the hypostomal ‘droplet’ described by Wernz (1972)? How do other subcapitular ridges interplay with any deutosternal mechanisms? These adornments can be quite extensive, e.g., in *Philippinozercon makilingensis*. As transverse ridges they can be offset out of line with the usual deutosternal cross ridges, e.g., *Dendroseius reductus*. Can evidence be found that they are a safety mechanism for pipe expansion to handle infrequent opportunistic extraordinary prey fluid volume overspills? Deutosternal denticles can be elongate in zerconids (Ujvári 2011), how might these function? They look like insect wing micro-scales illustrated by Jin et al. (2020). Are they equivalent to ‘pin-fins’ (Xie et al. 2021) known to cause complicated flow patterns in fluids moving past them in microchannels? Could droplets be held away from the subcapitular surface by the tessellated deutosternum in megisthanids much as regular polygonal cuticular structures are non-wetting in plants or insects? Could the tessellated pattern be the plesiomorphic state in mesostigmatids, or should it be a deutosternum like a central suture (at least in part as illustrated for the uropodoids *Trichuropoda (Oodinychus) ovalis* and *Trichouropoda dialveolata* by Hirschmann 1962)? Indeed is the tessellated pattern an example of part deutosternal longitudinal duplication that is also seen in megisthanids? What might holothyrids suggest? Measures of width and number of denticles on each deutosternal ridge, to inform or predict interpretation of their function (perhaps indicative of kinds of material ingested) for extinct/fossil kinds of mesostigmatid mites would be fascinating too (Evert Lindquist *pers. comm.*).

Beyond phylogenetic origin (Wernz 1972), why are some tritosterna simple and others very derived? Tritosterna in *Origmatrachys peruensis* (Uropodina) for example have four lacinae (Kontschán and Friedrich 2017). Going in the other direction of strangeness, why is fusion of lacinae so common? Seeman (2022) shows three genera in the trigynaspid family Schizogyniidae with differing degrees of fusion. How the various tritosternal sizes and shapes work hydrodynamically needs investigation in a follow-up study and integration into the scheme since it is already known that branched interfacial structures perform best for fluid de-pinning (Hensel et al. 2016). Wernz (1972) describes a variety of cross-sectional shapes to the lacinae themselves. Could the lacinae themselves be both a micro- and nano-scale tool for directional transport (see Hancock et al. 2012)? In some species the convex nature of the cross-bar ridges near the hypostomal tip might suggest that fluid flow backwards may be synergised, although the direction of the denticles on them themselves suggest that they are designed to facilitate flow anteriorly and encourage turbulent mixing when the lacinae are raised dorsally. Why is this so? How does an apically concave gutter transform to a convex surface along its length (see Wernz 1972)? How big is the internal diameter of any longitudinal nano-channel infoldings?

The part presence of ridges to the edge but not central to a groove facilitates the flow down the central axis of the channel (as in phytoseiids). Are there mites showing the opposite, that is central span-wise ridges *not* joining the (folded) deutosternal longitudinal edges? Could these then work like a *set* of interconnected capillary channels driving passive liquid transport as in Fig. 5 of Comanns et al. (2015)? Tritosterna are present in other arachnids (Dunlop 1996; Shultz 2007; Walter and Proctor 2013; de Miranda et al. 2022; Starck et al. 2022), and some arachnids have filtration mechanisms in their mouthparts (Dunlop 1994; Talarico et al. 2011). Can any relevant insights be gained from examining feeding and oral fluid handling in such non-acarines? Does their miniaturisation (Dunlop 2019) have similar morphological and hydrodynamic consequences as the trend from macrochelids to the phytoseiids?

At the size levels of subcapitular structures, fibrillate tritosternal lacinae must in some sense be pushing ‘thick water’ at an increased relative fluid viscosity like feather-winged insects (e.g., mymarids, thrips, some tiny beetles etc.) push ‘thick air’ at their scale of their wings. It is not clear how much different tritosterna can wiggle about to facilitate this (Wernz 1972 describes two musculatures driving some horizontal but mainly dorsal movement). However, no muscles are known which would allow its lacinae to wilfully flex and groom the groove other than passively as a raking mechanism on gnathosomal retraction (Wernz and Krantz 1976). A clogged groove might be expected in a mite species orally processing very heterogenous material. Are simple deutosternal/tritosternal assemblies associated with consuming homogeneous or heterogenous food? Further detailed ultrastructural studies of intra-gnathosomal musculature are needed. This review assumes that the tritosternal base can lift up and down and therefore the lacinae passively follow it, those fine hairs therefore must have something to do with grabbing and pushing the fluid mass up and down in some sort of squeezing way. The uropodine *Cilliba erlangensis* has a broad base, six lacinae tritosternum and seemingly no deutosternal groove (Babaeian et al. 2018). How does that function? Whatever is posed, precise hydrodynamics requires careful validation with biomimetic constructions in the laboratory.

So far the deutosternal assembly has been seen as a microfluidic liquid handling system and conclusions drawn from knowledge of man-made designed smooth-walled systems of different geometries (Hajialibabaei and Saghir 2022) with various disruptive structures like ribs, cavities, dimples, protrusions, secondary channels and other interrupts (Datta et al. 2019). Follow-up work is definitely needed to examine if the lipids and cuticular components vary along the mesostigmatid deutosternal groove or subcapitular regions and just how liquid repelling these might be. However, if the deutosternum was designed to hold (Erramilli and Genzer 2019), separate and sort cells or other suspended particulate material in that fluid, or needed to manipulate bubbles (using rectangular structures; Chindam et al. 2013) then a variety of passive continuous-flow separations could use forces induced by the channel topology itself (e.g., spiral, serpentine, expanded-contracted; Zhao et al. 2020) to manipulate such particles into differential equilibrium positions during their travel. These type of techniques cover hydrodynamic filtration, hydrophoresis and inertia-based continuous separations, as well as pinched flow fractionation and separation through deterministic lateral displacement. For entry points to this literature see Zhu et al. (2011) and Zhao et al. (2020). Could this be relevant to the cuticular tessellations on megisthanid subcapitula? Might their feeding fluids generated be very different in composition and thus need different handling?

However, it is not clear if, in fact, there are any major fractions of particles or cells in predatory mesostigmatid gnathosomal fluids which would need such sorting despite reports of debris associated with deutosternal grooves in general (Wernz 1972). Wernz and Krantz (1976) also mentions debris in tritosternectomised mites (then assigning denticles and lacinae as a grooming mechanism). Certainly the movement up and down of the tritosternum could deliver a dynamic geometry to the microchannel to achieve a known mechanism of cell-sorting (Gerhardt et al. 2011). Could particles perhaps arise from different sources in and around the cuticular structures of the ventral chitinous surfaces in general in species? Might this be the case in fungivorous mesostigmatids? However, deuterosternal microstructures visually do not decrease in size with subcapitular length (nor indeed with *IL*) as needed to increase van der Waals interactions (Erramilli and Genzer 2019) between such particles and the mite. Perhaps electrostatics come into play? Do cavernicolous palpigrades who use cheliceral adornments in collecting cyanobacteria from liquid (Smrž et al. 2013) have any oral structures like mesostigmatids? Is there any evidence of mucus in mite deutosternal grooves known to modulate fluid flow in fish (Tian et al. 2021)? This could be relevant in fungal and dry material feeding mesostigmatids. Alternatively, do high contact angle hydrophobic surfaces in mites encourage low adhesion of particles, as on insect wings (Hu et al. 2011) and leaves (Koch and Barthlott 2009; Barthlott et al. 2017) and are thus self-cleaning (Mondal et al. 2018)? To what extent could denticles be shown to be actually self-cleaning too? Indeed could superhydrophobicity (Sethi et al. 2019) ever actually be experimentally demonstrated in mesostigmatids?

Much detailed characterisation of mesostigmatid surface properties and fluid micro-samples in and around the gnathosoma is needed. There is much for keen acarologists to pursue!

## Conclusion

The Phytoseiidae, those most successful of arboreal predatory mites (Dave Walter *pers. comm.*), are an outlier functional group hydrodynamically. This review shows that the proposal (posed at the end of the “Introduction” section) is not true. For at least those aimed against tetranychids, candidate BCAs should not be chosen from meseostigmatid predators adapted to large volume prey fluid handling. Rather candidates for testing should be sought amongst micro- or mesocephalic ‘nibbling’ mesostigmatids of medium body size preferring relatively dry or low volume prey. Selection from these are by definition not ‘generalists’ and may be easier to license internationally.

**Table 1:** Details of female taxa examined arranged by family alphabetically then by increasing *AGW/GL* values within families

Taxon	References
ARCTACARIDAE	
Proarctacrus johnstoni*	Makarova (2003)
Proarctacarus canadensis*	Makarova (2003)
Proarctacrus oregonensis*	Makarova (2003)
ASCIDAE	
*Asca nelsoni*	Beard et al. (2011)
*Maxinia arctomontana*	Lindquist and Makarova (2012)
*Antenoseius bregatovae*	Joharchi et al. (2022a)
Arctoseius wisniewski*	Gwiazdowicz and Kamczyc (2009)
*Arctoseius koltschaki*	Makarova and Lindquist (2013)
*Proctogastrolaelaps subsolanus*	Joharchi et al. (2021)
Antennoseius perseus*	Beaulieu et al. (2008)
*Antennoseius pyrophilus*	Beaulieu et al. (2008)
*Arctoseius ambiguus*	Makarova and Huhta (2017)
*Arctoseius cetratus*	Keum et al. (2015)
Antennoseius perseus*	Beaulieu et al. (2008)
Arctoseius minutus*	Gwiazdowicz (2008)
*Arctoseius tschernovi*	Makarova (2000)
*Arctoseius miranalis*	Makarova (2000)
BLATTISOCIIDAE	
*Lasioseius orangrimbae*	Quintero-Gutiérrez et al. (2020a)
DIGAMESELLIDAE	
Dendroseius reductus*	Mašán (2020)
EVIPHIDIDAE	
*Alliphis necrophilus*	Christie (1983)
Alliphis halleri*	Halliday (2008)
Alliphis siculus*	Halliday (2008)
HETEROZERCONIDAE	
Philippinozercon makilingensis*	Gerdeman et al. (2018)
LAELAPIDAE	
*Ololaelaps formidabilis*	Beaulieu et al. (2019)
Gaeolaelaps jondishapouri*	Nemati and Kavianpour (2013), Kazemi et al. (2014)
*Ololaealaps tasmanicus*	Joharchi and Negm (2020)
*Gaeolaelaps iranicus*	Kavianpour et al. (2013)
Gaeolaelaps izajiensis*	Saeidi et al. (2016)
Cosmolaelaps longus*	Joharchi and Negm (2020)
*Cosmolaelaps siberiensis*	Joharchi et al. (2019)
*Gaeolaelaps aharangani*	Kazemi et al. (2014)
Gaeolaelaps gillespiei*	Beaulieu (2009)
*Gaeolaelaps mirzakhaniae*	Khalesi and Kazemi (2018)
MACROCHELIDAE	
*Macrocheles dispar*	Hartini and Takaku (2003)
Macrocheles sukabumiensis*	Hartini and Takaku (2003)
*Macrocheles pratum*	Knee (2017)
Macrocheles jabarensis*	Hartini and Takaku (2003)
*Macrocheles agilis*	Halliday (2000)
*Macrocheles kaju*	Knee (2017)
Neopodocinum longisetum*	Ács et al. (2016)
*Macrocheles willowae*	Knee (2017)
*Macrocheles jonggolensis*	Hartini and Takaku (2003)
Macrocheles kekensis*	Kontschán (2018)
*Macrocheles forceps*	Halliday (2000)
MEGISTHANIDAE	
*Megisthanus womersleyi*	Seeman (2019)
*Megisthanus modestus*	Seeman (2019)
*Megisthanus southcotti*	Seeman (2019)
*Megisthanus simoneae*	Seeman (2019)
MELICHARIDAE	
*Mucroseius insolitus*	Trach et al. (2019a)
Proctolaelaps sibiriensis*	Trach et al. (2019b)
NEOTHYRIDAE	
Diplothyrus lehtineni*	Vázquez et al. (2016)
PARASITIDAE	
Heteroparasitus mariae*	Witalińksi (2008)
*Thalassogamasus kurilensis*	Makarova (2019)
*Schizosthetus lyriformis*	Al-Atawi et al. (2002)
Thalassogamasus sidortschukae*	Makarova (2019)
*Occigamasus lindquisti*	Juvara-Bals (2019)
PARHOLASPIDAE	
Krantzolaspina angustatus*	Quintero-Gutiérrez et al. (2020b)
PHYTOSEIIDAE	
Amblyseius omaloensis*	Khaustov (2020)
Amblyseius silvaticus*	Khaustov (2020)
Amblyseius ampullosus*	Khaustov (2020)
Amblyseius myrtilli*	Khaustov (2020)
Proprioseiulus ceylonensis*	Khaustov et al. (2021)
Amblydromalus limonicus*	Ma et al. (2018)
RHODACARIDAE	
Binodacarus aceguensis*	Duarte et al. (2016)
Multidentorhodacarus matatlantica*	Castilho and de Moraes (2010)
*Multidentorhodacarus paulista*	Castilho and de Moraes (2010)
Binodacarus brasiliensis*	Castilho and de Moraes (2010)
*Multidentorhodacarus saboorii*	Castilho et al. (2012)
*Multidentorhodacarus tocantiensis*	Azevedo et al. (2019)
*Multidentotrhodacarus squamosus*	Azevedo et al. (2019)
*Rhodacarellus iraniensis*	Castilho et al. (2012)
UROPODIDAE	
*Uropoda abantica*	Bal and Özkan (2007)

*Species with ancillary lateral grooves, folds and other ridged/denticulated features outside of deutosternal groove contributing more than 20% of potential extra groove width

**Table 2:** Subcapitular measures taken in $\upmu$m (for an explanation of the column headings see Fig. 1)

Taxon	[1]	[2]	[3]	[4]	[5]	[6]	[7]	[8]
ARCTACARIDAE								
*Proarctacrus johnstoni*	106.1	197.9	166.4	14.8	269.7	66.3	121.9	241.6
*Proarctacarus canadensis*	135.8	235.7	195.9	19.2	342.5	69.9	136.0	307.5
*Proarctacrus oregonensis*	127.1	259.7	256.3	41.4	360.4	73.9	161.2	419.0
ASCIDAE								
*Asca nelsoni*	29.4	45.9	58.6	4.1	67.7	14.9	18.8	70.0
*Maxinia arctomontana*	44.2	79.9	76.5	6.0	123.5	25.0	30.0	74.5
*Anntenoseius bregatovae*	47.1	74.1	96.5	9.3	121.8	30.0	28.3	102.6
*Arctoseius wisniewski*	77.6	101.7	53.6	5.9	174.1	59.5	43.1	145.3
*Arctoseius koltschaki*	61.3	90.9	75.6	12.0	146.6	44.9	42.0	97.9
*Proctogastrolaelaps subsolanus*	37.5	46.3	44.3	7.2	78.9	17.7	19.8	66.7
*Antennoseius perseus (Phoretic)*	52.0	73.4	70.6	12.2	121.4	40.3	40.9	125.6
*Antennoseius pyrophilus (Phoretic)*	41.6	83.1	76.7	14.2	122.1	29.9	37.7	131.1
*Arctoseius ambiguus*	44.5	82.3	76.8	14.2	125.0	23.2	38.4	115.8
*Arctoseius cetratus*	53.8	58.2	50.8	9.6	86.6	16.9	29.1	92.0
*Antennoseius perseus*	52.6	77.0	71.7	13.6	125.7	34.9	36.2	128.0
*Arctoseius minutus*	18.8	48.3	44.4	9.4	67.1	11.3	21.9	63.4
*Arctoseius tschernovi*	66.7	106.5	80.9	19.0	169.1	48.3	62.0	158.1
*Arctoseius miranalis*	28.8	75.6	48.8	14.0	113.5	29.2	42.7	81.6
BLATTISOCIIDAE								
*Lasioseius orangrimbae*	35.9	58.9	45.4	8.8	94.3	25.4	31.8	100.6
DIGAMESELLIDAE								
*Dendroseius reductus*	31.4	53.4	53.0	8.7	82.1	19.0	30.6	81.8
EVIPHIDIDAE								
*Alliphis necrophilus*	45.6	73.2	69.4	10.6	103.5	25.5	43.1	81.0
*Alliphis halleri*	42.0	62.1	61.4	10.4	97.8	24.2	37.9	78.9
*Alliphis siculus*	42.1	52.6	62.5	11.5	93.9	22.8	34.2	80.8
HETEROZERCONIDAE								
*Philippinozercon makilingensis*	60.3	125.8	93.8	8.4	175.0	42.4	60.4	184.3
LAELAPIDAE								
*Ololaelaps formidabilis*	64.5	99.5	102.5	7.6	156.5	53.5	45.0	129.2
*Gaeolaelaps jondishapouri*	78.2	97.9	108.8	12.2	175.0	60.1	54.3	127.4
*Ololaealaps tasmanicus*			63.9	7.8			56.7	126.8
*Gaeolaelaps iranicus*	29.4	52.2	45.8	5.6	81.8	20.8	23.7	71.8
*Gaeolaelaps izajiensis*	47.1	67.4	67.4	8.3	112.6	29.6	32.0	109.9
*Cosmolaelaps longus*	62.0	92.4	81.3	11.0	144.6	39.1	48.2	
*Cosmolaelaps siberiensis*	37.8	55.5	50.5	7.0	91.6	18.7	26.9	83.4
*Gaeolaelaps aharangani*	59.3	82.4	79.3	13.5	142.4	44.3	40.7	121.9
*Gaeolaelaps gillespiei*	63.1	88.5	83.3	14.4	152.3	47.7	47.0	147.7
*Gaeolaelaps mirzakhaniae*	105.5	133.5	122.4	22.4	237.3	82.5	61.0	168.0
MACROCHELIDAE								
*Macrocheles dispar*	101.6		143.5	14.5	205.3	68.3	69.1	
*Macrocheles sukabumiensis*	113.7		151.3	16.3	233.2	68.1	76.7	
*Macrocheles pratum*	55.9	83.3	96.4	11.1	134.2	33.4	37.1	128.1
*Macrocheles jabarensis*	90.7		120.0	13.8	200.1	71.1	58.7	
*Macrocheles agilis*	105.2	124.8	149.5	17.9	220.3	73.2	62.6	140.5
*Macrocheles kaju*	62.9	111.3	119.8	16.1	169.3	38.3	42.4	134.0
*Neopodocinum longisetum*	124.3	104.3	182.5	28.6	235.7	58.6	104.2	233.3
*Macrocheles willowae*	60.8	69.6	81.4	13.2	130.9	43.3	43.8	128.2
*Macrocheles jonggolensis*	104.7		96.6	16.8		83.0	85.1	
*Macrocheles kekensis*	61.0	73.4	57.6	11.5	124.7	30.6	34.0	96.4
*Macrocheles forceps*	103.7		56.3	23.0		43.0	28.5	
MEGISTHANIDAE								
*Megisthanus womersleyi*	286.7	386.7	408.2	64.5	558.1	241.9	374.3	765.3
*Megisthanus modestus*	338.9	365.8	354.5	71.5	540.9	263.6	338.1	744.1
*Megisthanus southcotti*	355.3	356.9	262.7	60.3	509.9	222.4	334.4	656.7
*Megisthanus simoneae*	216.9	352.0	280.0	69.8	529.3	232.0	344.9	661.9
MELICHARIDAE								
*Mucroseius insolitus*	37.5	56.6	50.9	9.5	86.8	22.6	35.2	87.8
*Proctolaelaps sibiriensis*	34.1	46.1	45.0	8.9	79.7	19.2	19.8	71.6
NEOTHYRIDAE								
*Diplothyrus lehtineni*	163.7		184.2	26.7		75.3	253.5	
PARASITIDAE								
*Heteroparistus mariae*	62.7	117.9	131.1	14.7	161.6	42.7	65.3	125.5
*Thalassogamasus kurilensis*	64.6	110.8	82.3	9.9	162.7	38.6	65.4	118.2
*Schizosthetus lyriformis*	39.0	58.4	54.2	6.9	89.1	23.5	35.2	82.7
*Thalassogamasus sidortschukae*	89.5	168.1	119.6	16.4	239.4	57.5	106.3	204.2
*Occigamasus lindquisti*	75.6	158.3	115.7	20.6	199.8	48.2	83.7	178.5
PARHOLASPIDAE								
*Krantzolaspina angustatus*	102.5	96.7	74.2	10.1	202.5	93.3	56.8	122.1
PHYTOSEIIDAE								
*Amblyseius omaloensis*	40.8	51.2	49.6	3.1	89.0	20.8	20.0	72.8
*Amblyseius silvaticus*	53.2	64.5	64.5	4.4	115.3	28.6	22.3	90.2
*Amblyseius ampullosus*	41.4	48.1	48.4	3.6	87.9	19.0	17.2	76.8
*Amblyseius myrtilli*	44.2	53.6	53.9	4.0	98.3	25.9	21.7	90.1
*Proprioseiulus ceylonensis*	37.0	48.5	47.2	4.3	82.7	26.5	22.7	81.8
*Amblydromalus limonicus*	52.5		66.2	6.4		39.0	31.2	108.3
RHODACARIDAE								
*Binodacarus aceguensis*	38.1	58.0	52.3	3.8	82.4	27.8	38.7	77.1
*Multidentorhodacarus matatlantica*	37.3	63.9	62.0	5.5	101.5	29.4	39.5	75.5
*Multidentorhodacarus paulista*	53.0	50.7	52.2	5.8	90.0	33.3	36.5	76.5
*Binodacarus brasiliensis*	27.2	34.6	33.3	3.8	55.1	19.7	19.3	51.8
*Multidentorhodacarus saboorii*	48.8	50.9	40.9	5.2	86.1	28.5	32.1	67.7
*Multidentorhodacarus tocantiensis*	30.2		42.2	5.6		15.1	27.4	68.9
*Multidentotrhodacarus squamosus*	28.8		34.0	4.8		14.7	24.1	68.5
*Rhodacarellus iraniensis*	39.0	41.8	35.7	5.6	67.5	21.2	27.7	68.0
UROPODIDAE								
*Uropoda abantica*	34.7	54.7	26.2	23.0	72.4	23.6	25.9	54.4

[2] = *BGL*. [3] = *GL* (with no extensions). [4] = *AGW* (with no lateral extensions). [8] = *BGW*

**Table 3:** Cheliceral measures taken (for an explanation of the column headings see Glossary of abbreviations in the Materials and Methods section)

Taxon	IL	DSL	BSL	Reach (CLI)	Gape (MDL)	VR	CSL	CHI
ARCTACARIDAE								
*P. johnstoni*	563.9	346.7	113.4	460.1	204.2	0.141	255.9	130.5
*P. canadensis*	598.0	375.5	134.9	510.4	205.9	0.152	304.5	112.1
*P. oregonensis*	714.3				235.6	0.177		142.0
ASCIDAE								
*A. nelsoni*	140.1	65.6	33.6	99.2	23.3	0.298	75.9	22.3
*M. arctomontana*	188.8				42.5	0.210		24.3
*A. bregatovae*	255.8				42.3	0.271		34.1
*A. wisniewski*	293.2				48.0	0.306		35.8
*A. koltschaki*	288.3	141.0	70.6	211.6	52.3	0.378	159.4	50.9
*P. subsolanus*	192.1	68.6	31.3	99.9	29.2	0.373	70.7	21.6
*A. perseus (Phoretic)*	290.9				42.3	0.288		40.8
*A. pyrophilus (Phoretic)*					37.7	0.289		33.7
*A. ambiguus*	283.3	112.2	51.1	163.3	48.0	0.278	115.3	47.8
*A. cetratus*								
*A. perseus*					49.3	0.321		43.6
*A. minutus*	171.2							
*A. tschernovi*	296.2				52.5	0.203		48.1
*A. miranalis*					55.0	0.236		34.4
BLATTISOCIIDAE								
*L. orangrimbae*	183.9	85.6	38.9	124.5	36.7	0.328	87.8	29.8
DIGAMESELLIDAE								
*D. reductus*	168.1	88.5	40.8	129.3	37.3	0.221	92.0	32.5
EVIPHIDIDAE								
*A. necrophilus*	243.8	133.4	65.3	198.8	51.3	0.240	147.5	35.4
*A. halleri*	180.2				40.0	0.295		30.4
*A. siculus*	227.8				41.2	0.229		24.2
HETEROZERCONIDAE								
*P. makilingensis*	267.0	187.7	60.3	248.0	112.0	0.155	136.0	51.7
LAELAPIDAE								
*O. formidabilis*	246.0	164.1	73.7	237.8	71.5	0.233	166.3	40.1
*G. jondishapouri*	261.3	180.1	69.0	249.1	72.4	0.251	176.7	57.5
*O. tasmanicus*	312.5				69.8	0.272		44.9
*G. iranicus*	195.1				37.7	0.327		24.9
*G. izajiensis*	187.8	124.5	47.5	172.0	64.8	0.281	107.2	43.4
*C. longus*	317.0				61.0	0.307		45.5
*C. siberiensis*	232.7	90.0	48.3	138.2	29.3	0.261	109.0	19.0
*G. aharangani*	249.0	143.7	66.9	210.6	59.6	0.244	151.0	46.7
*G. gillespiei*	312.7				68.6	0.264		39.4
*G. mirzakhaniae*	393.7	232.9	97.4	330.4	110.4	0.186	219.9	76.9
MACROCHELIDAE								
*M. dispar*	328.4	206.0	99.6	305.6	80.9	0.357	224.7	60.8
*M. sukabumiensis*	387.9	207.6	95.4	302.9	87.7	0.241	215.3	89.3
*M. pratum*	241.7				44.4	0.475		35.1
*M. jabarensis*	354.7	178.2	83.2	261.4	73.6	0.379	187.8	52.9
*M. agilis*					63.7	0.339		49.6
*M. kaju*	351.1				60.0	0.293		37.5
*N. longisetum*	536.9	224.7	74.6	299.3	130.9	0.264	168.4	90.4
*M. willowae*	226.7				50.7	0.377		47.1
*M. jonggolensis*	380.6	198.9	87.6	286.5	88.7	0.364	197.7	95.2
*M. kekensis*	250.9				51.6	0.462		37.1
*M. forceps*					91.0	0.229		42.2
MEGISTHANIDAE								
*Me. womersleyi*	1255.1					0.259		
*M. modestus*								
*M. southcotti*	1087.3							
*M. simoneae*	1215.5							
MELICHARIDAE								
*M. insolitus*	244.7	87.2	44.0	131.1	31.9	0.328	99.3	37.0
*P. sibiriensis*	181.5	54.7	29.4	84.1	17.8	0.444	66.3	21.9
NEOTHYRIDAE								
*D. lehtineni*					110.5	0.274		88.3
PARASITIDAE								
*H. mariae*					77.5	0.295		61.7
*T. kurilensis*	291.6	190.2	69.9	260.1	102.3	0.161	157.7	68.9
*S. lyriformis*	568.6							
*T. sidortschukae*	447.8	288.1	94.3	382.5	169.5		212.9	90.2
*O. lindquisti*					124.9	0.263		61.2
PARHOLASPIDAE								
*K. angustatus*	365.8				100.8	0.213		46.6
PHYTOSEIIDAE								
*A. omaloensis*	189.9	94.2	44.5	138.7	38.3	0.338	100.4	28.0
*A. silvaticus*	213.7	84.1	34.8	118.9	40.5	0.425	78.4	36.0
*A. ampullosus*	204.9	77.4	38.8	116.2	28.6	0.436	87.6	27.4
*A. myrtilli*	234.0	81.3	40.8	122.1	29.9	0.348	92.1	27.8
*P. ceylonensis*	142.7	61.5	30.3	91.8	23.4	0.325	68.4	23.1
*A. limonicus*								
RHODACARIDAE								
*B. aceguensis*	177.5				47.6	0.265		40.1
*M. matatlantica*	159.2				57.0			40.0
*M. paulista*	149.5				36.1			24.4
*B. brasiliensis*	101.3				32.6			27.1
*M. saboorii*	161.2				56.9	0.150		42.5
*M. tocantiensis*	128.4				55.3			29.2
*M. squamosus*	159.1				66.8			30.7
*R. iraniensis*	152.1				36.5	0.347		30.5
UROPODIDAE								
*U. abantica*	397.0	119.0	40.5	159.6	26.4	0.364	133.2	18.2

Lengths and heights in $\upmu$m. Velocity ratio (*VR*) is unit-less. $V_{m}=\tfrac{\pi }{2}.\tfrac{IL^{3}}{10^{6}}$

**Table 4:** Other morphological measures taken (for an explanation of the column headings see Glossary of abbreviations in the Materials and Methods section)

Taxon	F1	F2	AGW+	No. ridges	No. denticles	Number denticles$\ddag$	Cephalic status
ARCTACARIDAE							
*P. johnstoni*	17017.9	2405.1	76.2	11	5–54	29.5	Mega
*P. canadensis*	12555.7	1903.7	32.9	10	4–36	20	Mega
*P. oregonensis*	20175.6	3562.6	185.4	11	9–76	42.5	Mega
ASCIDAE							
*A. nelsoni*	495.4	147.7	4.9	9	1–3	2	Micro
*M. arctomontana*	592.4	124.7	6.0	9	0–6	3	Micro
*A. bregatovae*	1162.9	314.7	9.3	9	0–4	2	Micro
*A. wisniewski*	1280.0	392.0	9.4	9	Many	15	Micro
*A. koltschaki*	2590.9	980.0	12.0	9	0–15	7.5	Meso
*P. subsolanus*	468.0	174.7	7.2	9	0–16	8	Micro
*A. perseus (Phoretic)*	1662.7	478.3	20.1	8	3–8	5.5	Meso
*A. pyrophilus (Phoretic)*	1138.9	329.3	14.6	9	Some	5	Mega
*A. ambiguus*	2285.5	634.6	14.2	9	Many	15	Meso
*A. cetratus*			9.6	5			
*A. perseus*	1897.1	609.5	22.6	8	Many	15	Mega
*A. minutus*			11.5	8	lots	25	
*A. tschernovi*	2312.6	470.5	19.0	8	Lots	25	Meso
*A. miranalis*	1186.0	280.1	14.0	7	Lots	25	Mega
BLATTISOCIIDAE							
*L. orangrimbae*	885.8	290.4	9.1	8	0–Many	7.5	Micro
DIGAMESELLIDAE							
*D. reductus*	1055.6	233.0	21.4	7	Lots	25	Meso
EVIPHIDIDAE							
*A. necrophilus*	1253.3	300.7	10.6	9	0–5	2.5	Micro
*A. halleri*	922.2	271.8	30.5	7	Many	15	Micro
*A. siculus*	583.4	133.6	24.2	6	Many	15	Micro
HETEROZERCONIDAE							
*P. makilingensis*	2674.2	414.3	57.1	7			Mega
LAELAPIDAE							
*O. formidabilis*	1611.1	375.0	7.6	8	Few	2.5	Meso
*G. jondishapouri*	3307.8	831.4	16.9	9	0–3	1.5	Mega
*O. tasmanicus*	2014.9	548.2	7.8	5	3	3	Meso
*G. iranicus*	618.6	202.1	6.5	6	Some	5	Micro
*G. izajiensis*	1881.7	528.6	12.5	8	Some	5	Meso
*C. longus*	2069.0	635.6	19.4	7	8–18	13	Meso
*C. siberiensis*	361.0	94.2	8.1	6	7–12	9.5	Micro
*G. aharangani*	2181.1	533.1	14.7	9	Lots	25	Meso
*G. gillespiei*	1554.3	409.8	21.6	7			Micro
*G. mirzakhaniae*	5919.5	1103.8	24.5	8	Lots	25	Mega
MACROCHELIDAE							
*M. dispar*	3698.7	1318.9	16.3	7	Some	5	Mega
*M. sukabumiensis*	7981.7	1923.3	22.3	6	Lots	25	Mega
*M. pratum*	1234.6	586.7	11.1	7	1–8	4.5	Micro
*M. jabarensis*	2796.6	1058.9	17.6	6	Lots	25	Meso
*M. agilis*	2458.0	834.3	17.9	6	Many	15	Mega
*M. kaju*	1408.5	412.7	16.1	7	1–10	5.5	Micro
*N. longisetum*	8174.1	2159.6	42.3	9	Lots	25	Mega
*M. willowae*	2213.9	834.7	13.2	7	1–10	5.5	Meso
*M. jonggolensis*	9069.7	3301.4	16.8	6	Many	15	Mega
*M. kekensis*	1379.3	637.6	13.8	6	Some	5	Micro
*M. forceps*	1781.7	407.3	23.0	5	Some	5	Mega
MEGISTHANIDAE							
*Me. womersleyi*			64.5	5	Many	15	
*M. modestus*			71.5	7			
*M. southcotti*			60.3	4			
*M. simoneae*			69.8	5			
MELICHARIDAE							
*M. insolitus*	1370.7	449.9	11.2	9	4–19	11.5	Meso
*P. sibiriensis*	481.4	213.9	11.9	9	0–3	1.5	Micro
NEOTHYRIDAE							
*D. lehtineni*	7801.4	112.7	65.8	2			Mega
PARASITIDAE							
*H. mariae*	3801.9	1119.8	26.7	14	Some	5	Mega
*T. kurilensis*	4746.9	766.0	9.9	11	4–7	5.5	Mega
*S. lyriformis*			6.9	9	Some	5	
*T. sidortschukae*	8135.3		23.9	13	0–10	5	Mega
*O. lindquisti*	3741.1	985.8	20.6	11	Some	5	Mega
PARHOLASPIDAE							
*K. angustatus*	2173.6		24.6	6	Lots	25	Meso
PHYTOSEIIDAE							
*A. omaloensis*	784.4	265.1	12.1	8	2	2	Micro
*A. silvaticus*	1297.5	551.7	11.0	9	0–2	1	Meso
*A. ampullosus*	750.4	326.8	12.4	9	0–2	1	Micro
*A. myrtilli*	775.5	269.7	6.5	9	0–2	1	Micro
*P. ceylonensis*	535.6	174.2	15.4	10	0–2	1	Micro
*A. limonicus*			11.7	6	0–1	1	
RHODACARIDAE							
*B. aceguensis*	1607.4	425.8	6.6	9	Some	5	Meso
*M. matatlantica*	1603.2		12.9	9			Meso
*M. paulista*	596.3		6.9	8	Many	15	Micro
*B. brasiliensis*	736.0		5.2	8	0–1	0.5	Meso
*M. saboorii*	1808.1	271.4	5.2	8			Mega
*M. tocantiensis*	853.9		5.6	8			Meso
*M. squamosus*	943.0		4.8	8			Meso
*R. iraniensis*	930.1		5.6	7	Lots	25	Meso
UROPODIDAE							
*U. abantica*	333.0	121.1	15.3	5	Lots	25	Micro

Forces in $\upmu$m$^{2}$ (see Bowman 2020). $AGW+$ includes lateral extensions and is in $\upmu$m. Ridges are “Querleisten" Hirschmann (1962). $\ddag$Deutosternal denticles after heuristic mapping

**Table 5:** Other volume-based measures taken

Taxon	[A]	[B]	DV	*PFRt*	$tep^{*}/10^{6}$	Wavelength	$\lambda$	Bite-size/$10^{6}$
ARCTACARIDAE								
P. johnstoni*	0.1667	5.0922	0.0287	290	6.9	16.6	0.04	421,992
P. canadensis*	0.3544	4.1674	0.0564	687	13.7	21.8	0.06	517,383
P. oregonensis*	2.2576		0.3452	11,479	133.9	25.6	0.03	727,852
ASCIDAE								
*A. nelsoni*	0.0037	0.0320	0.0008	5	7.6	7.3	0.58	3078
*M. arctomontana*	0.0084		0.0021	17	10.5	9.6	0.38	8060
*A. bregatovae*	0.0277		0.0065	76	19.4	12.1	0.58	11,358
A. wisniewski*	0.0159		0.0015	23	3.8	6.7	0.08	29,910
*A. koltschaki*	0.0445	0.3566	0.0086	277	49.2	9.4	0.15	32,751
*P. subsolanus*	0.0109	0.0304	0.0018	61	36.4	5.5	0.14	4016
A. perseus (Phoretic)*	0.0585		0.0082	311	53.6	10.1	0.21	21,667
*A. pyrophilus (Phoretic)*	0.0825		0.0121	523		9.6	0.23	13,249
*A. ambiguus*	0.0733	0.2427	0.0122	528	98.8	9.6	0.08	20,449
*A. cetratus*	0.0264		0.0036	164		12.7	0.60	
A. perseus*	0.0744		0.0104	478		10.2	0.08	20,768
A. minutus*	0.0178		0.0031	179	152.0	6.3	0.05	
*A. tschernovi*	0.1785		0.0228	1595	260.9	11.6	0.05	49,703
*A. miranalis*	0.0502		0.0075	784		8.1	0.05	10,593
BLATTISOCIIDAE								
*L. orangrimbae*	0.0245	0.0717	0.0028	132	90.3	6.5	0.15	8502
DIGAMESELLIDAE								
D. reductus*	0.0195	0.0887	0.0032	109	97.2	8.8	0.05	7792
EVIPHIDIDAE								
*A. necrophilus*	0.0284	0.1620	0.0061	179	52.5	8.7	0.46	17,419
A. halleri*	0.0269		0.0052	192	139.5	10.2	0.08	12,107
A. siculus*	0.0335		0.0065	278	100.0	12.5	0.08	8707
HETEROZERCONIDAE								
P. makilingensis*	0.0404	0.4313	0.0051	52	11.6	15.6	0.53	47,145
LAELAPIDAE								
*O. formidabilis*	0.0232	0.2492	0.0046	32	9.1	14.6	0.46	29,143
G. jondishapouri*	0.0599	0.5358	0.0128	206	49.0	13.6	0.77	61,014
*O. tasmanicus*	0.0241		0.0030	57	8.0	16.0	0.38	
*G. iranicus*	0.0071		0.0011	21	12.3	9.2	0.23	4327
G. izajiensis*	0.0238	0.2104	0.0037	71	45.5	9.6	0.23	16,356
C. longus*			0.0078	182	24.3	13.6	0.09	34,026
*C. siberiensis*	0.0130	0.0325	0.0020	49	16.5	10.1	0.12	4826
*G. aharangani*	0.0701	0.2987	0.0114	423	116.4	9.9	0.05	28,193
G. gillespiei*	0.0966		0.0136	520	72.3	13.9	0.91	29,247
*G. mirzakhaniae*	0.2660	1.2717	0.0485	2075	144.6	17.5	0.05	123,830
MACROCHELIDAE								
*M. dispar*		0.7349	0.0238	312	37.4	23.9	0.23	106,838
M. sukabumiensis*		1.5724	0.0317	470	34.2	30.3	0.05	194,810
*M. pratum*	0.0494		0.0093	157	47.2	16.1	0.26	18,195
M. jabarensis*		0.4754	0.0179	302	28.8	24.0	0.05	70,394
*M. agilis*	0.1408		0.0374	680		29.9	0.08	81,628
*M. kaju*	0.1090		0.0243	559	54.9	20.0	0.21	25,021
N. longisetum*	0.5982	1.5909	0.1169	3650	100.3	22.8	0.05	292,711
*M. willowae*	0.0698		0.0111	369	134.6	13.6	0.21	31,344
*M. jonggolensis*		1.6896	0.0214	822	63.4	19.3	0.08	212,173
M. kekensis*	0.0399		0.0060	301	81.2	11.5	0.23	19,218
*M. forceps*			0.0234	4958		14.1	0.23	31,207
MEGISTHANIDAE								
*Me. womersleyi*	10.0134		1.3352	42,494	91.4	102.0	0.08	
*M. modestus*	11.9607		1.4246	73,843		59.1	4.52	
*M. southcotti*	7.4977		0.7500	50,275	166.3	87.6	3.81	
*M. simoneae*	10.1209		1.0703	84,615	200.4	70.0	4.41	
MELICHARIDAE								
*M. insolitus*	0.0251	0.1169	0.0036	162	47.1	6.4	0.10	12,235
P. sibiriensis*	0.0178	0.0263	0.0028	140	99.3	5.6	0.77	3700
NEOTHYRIDAE								
D. lehtineni*			0.1033	2768		184.2	1.69	916,342
PARASITIDAE								
H. mariae*	0.0852		0.0222			10.1	0.23	63,037
*T. kurilensis*	0.0363	0.8028	0.0063	116	20.0	8.2	0.21	72,748
*S. lyriformis*	0.0124		0.0020	42	1.0	6.8	0.23	
T. sidortschukae*	0.1718	2.0234	0.0252	600	28.4	10.0	0.23	214,526
*O. lindquisti*	0.2383		0.0386	1562		11.6	0.23	96,766
PARHOLASPIDAE								
K. angustatus*	0.0389		0.0059	139	12.0	14.8	0.05	67,831
PHYTOSEIIDAE								
A. omaloensis*	0.0022	0.0708	0.0004	2	1.1	7.1	0.58	5725
A. silvaticus*	0.0056	0.1003	0.0010	6	2.6	8.1	1.15	10,705
A. ampullosus*	0.0031	0.0567	0.0005	3	1.7	6.0	1.15	4868
A. myrtilli*	0.0046	0.0616	0.0007	5	1.6	6.7	1.15	6689
P. ceylonensis*	0.0047	0.0320	0.0007	7	10.2	5.2	1.15	4864
A. limonicus*	0.0138		0.0021	25		13.2	1.15	
RHODACARIDAE								
B. aceguensis*	0.0035		0.0006	4	3.0	6.5	0.23	14,776
M. matatlantica*	0.0071		0.0015	15	15.4	7.8	0.35	14,738
*M. paulista*	0.0080		0.0014	21	26.7	7.5	0.08	11,799
B. brasiliensis*	0.0023		0.0004	6	25.5	4.8	2.31	3556
*M. saboorii*	0.0057		0.0009	17	17.7	5.8	0.33	16,657
*M. tocantiensis*	0.0068		0.0010	23	46.9	6.0	0.35	6045
*M. squamosus*	0.0049		0.0006	15	16.0	4.9	0.30	5347
*R. iraniensis*	0.0068		0.0009	28	33.8	6.0	0.05	8233
UROPODIDAE								
*U. abantica*	0.0900	0.0346	0.0108	10,595	720.2	6.6	0.05	4097

[A] = Circumcapitular groove volume ($\upmu$m$^{3}$) estimated by $\pi \tfrac{BGW.(AGW^{2})}{10^{6}}$. [B] = Volume available from a single cheliceral protrusion/retraction (‘Tubevolume’, $\upmu$m$^{3}$) estimated by $\pi \tfrac{0.828}{4}.\tfrac{Reach.(CHI^{2})}{10^{6}}$. DV = Deutosternal groove volume ($\upmu$m$^{3}$) estimated by $\tfrac{\pi }{4}.(\tfrac{GL.(AGW^{2})}{10^{6}}$. $tep^{*}$ = Trophic efficiency parameter estimate (using tetranychid $V_{p}$ from Table 6 and $\tfrac{V_{m}}{10^{6}}$). Wavelength = average inter-Querleisten spacing in $\upmu$m. For an explanation of the other column headings see Glossary of abbreviations in the Materials and Methods section

**Table 6:** Details of some potential phytoseiid mesostigmatids as tetranychid BCAs

Species	*IL* ($\upmu$m)	$V_{m}$ ($\upmu {\text {m}}^{3}$/$10^{6}$)	*VR*	Reach *CLI* ($\upmu {\text {m}}^{3}$)	$\frac{CLI}{IL}$	MDL ($\upmu$m)	*F*2	Groove width *AGW*($\upmu$m)	Groove length *GL* ($\upmu$m)	No. ridges	Wavelength ($\upmu$m)	No. denticles
*Amblyseius largoensis*			0.472									
*Amblyseius limonicus*								4–6		7		
*Amblyseius okagensis*	395.3	97.03	0.385	127.9	0.324	33.2	470.6					
*Amblyseius similoides*								4–6		7		
*Amblyseius swirski*			0.485									
*Euseius hibisci*								7–9		7		
*Euseius ovalis*			0.362									
*Euseius scutalis*			0.349					7–9		7		
*Euseius stipulatus*								7–9		7		
*Euseius tularensis*								7–9		7		
*Euseius utilis*$\dag$			0.443	90.3			454.3		42.9	7	6.1	2
*Galendromus annectens*								4–6		7		
*Galendromus helveolus*								4–6		7		
*Galendromus occidentalis*								4–6		7		1–2
*Galendromus piersi*								4–6		7		
*Iphiseius degenerans*			0.418					7–9		7		
*Kampimodromus aberrans*			0.387									
*Neioseiulus californicus*			0.550					4–6		7		
*Neioseiulus cucumeris*			0.442									
*Phytoseiulus fragariae*								4–6		7		2–5
*Phytoseiulus longipes*			0.397					4–6		10–12		1-5
*Phytoseiulus macropilis*								4–6		7–8		2–3
*Phytoseiulus persimilis*			0.436					4–6		8		2–3
*Phytoseiulus plumifer*			0.376									
*Typhlodromus exhilaratus*			0.375									
*Typhlodromus pyri*			0.408									
*Typhlodromus rickeri*								4–6		7		
*Typhlodromus setubali*	313.4	48.35	0.450	107.9	0.344	24.6	407.7					
Specialist$\ddag$			0.454	91.4			347.4		50.1	7.67	6.5	2.67
Generalist$\ddag$			0.460	113.2			556.9		53.0	7	7.6	2

$\dag$Deemed pollen feeder by Liu et al. (2017), $\ddag$For species included see Liu et al. (2017). For a single typical tetranychid of $IL=162\ \upmu$m, $\hat{V_{p}}\approx 6.68\ \upmu {\text {m}}^{3}/10^{6}$. Data abstracted from: Flechtmann and McMurtry (1992b), Flechtmann et al. (1994), Liu et al. (2017), and Bowman (2020)
